# Bond-length distributions for ions bonded to oxygen: alkali and alkaline-earth metals

**DOI:** 10.1107/S2052520616008507

**Published:** 2016-08-01

**Authors:** Olivier Charles Gagné, Frank Christopher Hawthorne

**Affiliations:** a Geological Sciences, University of Manitoba, 125 Dysart Road, Winnipeg, Manitoba R3T 2N2, Canada

**Keywords:** bond lengths, coordination number, alkali metals, alkaline-earth metals

## Abstract

Bond-length distributions have been examined for 55 configurations of alkali-metal ions and 29 configurations of alkaline-earth-metal ions bonded to oxygen, for 4859 coordination polyhedra and 38 594 bond distances (alkali metals), and for 3038 coordination polyhedra and 24 487 bond distances (alkaline-earth metals). Bond lengths generally show a positively skewed Gaussian distribution that originates from the variation in Born repulsion and Coulomb attraction as a function of interatomic distance. The skewness and kurtosis of these distributions generally decrease with increasing coordination number of the central cation, a result of decreasing Born repulsion with increasing coordination number. We confirm the following minimum coordination numbers: ^[3]^Li^+^, ^[3]^Na^+^, ^[4]^K^+^, ^[4]^Rb^+^, ^[6]^Cs^+^, ^[3]^Be^2+^, ^[4]^Mg^2+^, ^[6]^Ca^2+^, ^[6]^Sr^2+^ and ^[6]^Ba^2+^, but note that some reported examples are the result of extensive dynamic and/or positional short-range disorder and are not ordered arrangements. Some distributions of bond lengths are distinctly multi-modal. This is commonly due to the occurrence of large numbers of structure refinements of a particular structure type in which a particular cation is always present, leading to an over-representation of a specific range of bond lengths. Outliers in the distributions of mean bond lengths are often associated with anomalous values of atomic displacement of the constituent cations and/or anions. For a sample of ^[6]^Na^+^, the ratio *U*_eq(Na)_/*U*_eq(bonded anions)_ is partially correlated with 〈^[6]^Na^+^—O^2−^〉 (*R* ^2^ = 0.57), suggesting that the mean bond length is correlated with vibrational/displacement characteristics of the constituent ions for a fixed coordination number. Mean bond lengths also show a weak correlation with bond-length distortion from the mean value in general, although some coordination numbers show the widest variation in mean bond length for zero distortion, *e.g.* Li^+^ in [4]- and [6]-coordination, Na^+^ in [4]- and [6]-coordination. For alkali-metal and alkaline-earth-metal ions, there is a positive correlation between cation coordination number and the grand mean incident bond-valence sum at the central cation, the values varying from 0.84 v.u. for ^[5]^K^+^ to 1.06 v.u. for ^[8]^Li^+^, and from 1.76 v.u. for ^[7]^Ba^2+^ to 2.10 v.u. for ^[12]^Sr^2+^. Bond-valence arguments suggest coordination numbers higher than [12] for K^+^, Rb^+^, Cs^+^ and Ba^2+^.

## Abstract 2

Bond-length distributions have been examined for 55 configurations of alkali-metal ions and 29 configurations of alkaline-earth-metal ions, for 4859 coordination polyhedra and 38 594 bond distances (alkali metals), and for 3038 coordination polyhedra and 24 487 bond distances (alkaline-earth metals).

## 1. Introduction   

Many crystal structures have been refined in the past 100 years, and a large amount of information concerning inter­atomic distances in the solid state is available. There are many studies of bond-length distributions for specific pairs of ions, notably for cations bonded to oxygen [*e.g.* Baur, 1971 ▸ (Si); Burns *et al.*, 1997 ▸ (U); Hawthorne *et al.*, 2000 ▸ (S); Schindler *et al.*, 2000 ▸ (V); Hawthorne & Huminicki, 2002 ▸ (Be); Huminicki & Hawthorne, 2002 ▸ (P); Mills & Christy, 2013 ▸ (Te); Majzlan *et al.*, 2014 ▸ (As)]. However, many of these studies have focused on subsets of the available information, both with regard to the number of ions and coordination numbers, and to the amount of data available for each example. We have examined the distribution of bond lengths for 135 ions bonded to oxygen in 462 configurations using 180 331 bond lengths extracted from 9367 refined crystal structures; these data involve most ions of the periodic table and all coordination numbers in which they occur. Here we report the bond-length distributions for 10 ions, the common alkali-metal ions (Li^+^, Na^+^, K^+^, Rb^+^ and Cs^+^) and alkaline-earth-metal ions (Be^2+^, Mg^2+^, Ca^2+^, Sr^2+^ and Ba^2+^) in all observed coordination numbers where bonded to O^2−^ for a total of 63 081 bond lengths in 7897 polyhedra from 4258 refined crystal structures. An advantage of working with a large number of ion pairs and a large amount of data is that it allows examination of subtle differences between the shapes of various distributions (*e.g.* bond-length distributions, mean-bond-length distributions) for various configurations of ions, which reflect differences in their bonding behaviour. These differences typically arise from either structural and/or electronic effects, and are well known for extreme examples such as [6]-coordinated Cu^2+^ and [6]-, [7]- and [8]-coordinated U^6+^; however, more subtle deviations from unimodality could be expected for the bond-length distributions of other ion configurations that are involved in related electronic or structural effects. Our motivation for this work is twofold: (1) The factors that affect bond distances are of continuing interest to all who work on crystal structures and their properties, and a comprehensive analysis of all the data should lead to increased understanding of those factors. Here we give a preliminary examination of the alkali-metal ions and alkaline-earth-metal ions in all observed coordination numbers where bonded to O^2−^, and make our complete dataset available for future more detailed work. (2) A comprehensive knowledge of the observed variation in bond lengths is critically important in assessing the validity of computational results on possible atomic arrangements (*e.g.* Richardson, 2013 ▸) and identifying unusual stereochemical features in newly solved or refined crystal structures.

## 2. Definitions   

In the interest of clarity, we define certain terms that we use in the following text. We make no claims of generality; these are merely working definitions.

*Chemical bond*: There is no rigorous definition of a chemical bond that is useful in the context of the present work, which deals with some hundreds of thousands of observed inter­atomic distances. The decision on whether or not a specific interatomic distance corresponds to a chemical bond is made in terms of the local environment of the constituent atoms, *e.g.* is the distance consistent with a specific coordination number of the central ion, and is the valence-sum rule (Brown, 2002 ▸) reasonably well satisfied for the constituent ions? These are the criteria that are generally used for the listing of bond lengths in crystal-structure papers.

*Coordination number*: the number of counterions bonded to an ion.

*Coordination polyhedron*: The arrangement of counterions around an ion.

*Ion configuration*: A unique arrangement of ion type and coordination number.

**Figure 1:**
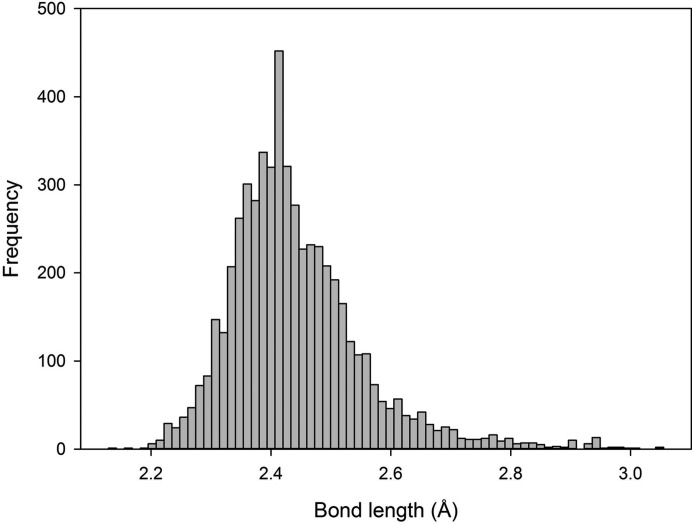
A typical distribution of bond lengths, shown for ^[6]^Na^+^ bonded to O^2−^ (*n* = 5532).

*Typical distribution*: A distribution that is smooth and positively skewed, as for ^[6]^Na^+^ (Fig. 1 ▸).

## 3. Coordination polyhedra   

### 3.1. Coordination polyhedra with the same coordination number   

Most coordination numbers may show more than one coordination polyhedron. For example, [4]-coordination may be tetrahedral or square planar, [5]-coordination may be trigonal bipyramidal or square pyramidal, [6]-coordination may be octahedral or trigonal prismatic. However, within the bond-valence model (Brown, 2002 ▸), differences in angular arrangement of counterions have no effect on the valence sums, and hence we do not differentiate between different spatial configurations of coordination polyhedra with the same coordination number.

### 3.2. The longest bond   

The determination of coordination number depends on how one defines a chemical bond (see ‘definitions’ section). It is fortunate that for many (if not most) crystal structures, there is a general consensus as to the coordination numbers observed. Thus rutile has Ti^4+^ in [6]-coordination and O^2−^ in [3]-coordination, and quartz has Si^4+^ in [4]-coordination and O^2−^ in [2]-coordination. For cations making a small number of bonds to their counterions, the determination of the coordination polyhedron is usually straightforward, and the coordination number can be assumed with confidence (*e.g.* for Mg^2+^, Fe^2+^, Si^4+^). However, making a decision on what is the longest bond for cations making a large number of bonds to their counterions is often less straightforward.

**Figure 2:**
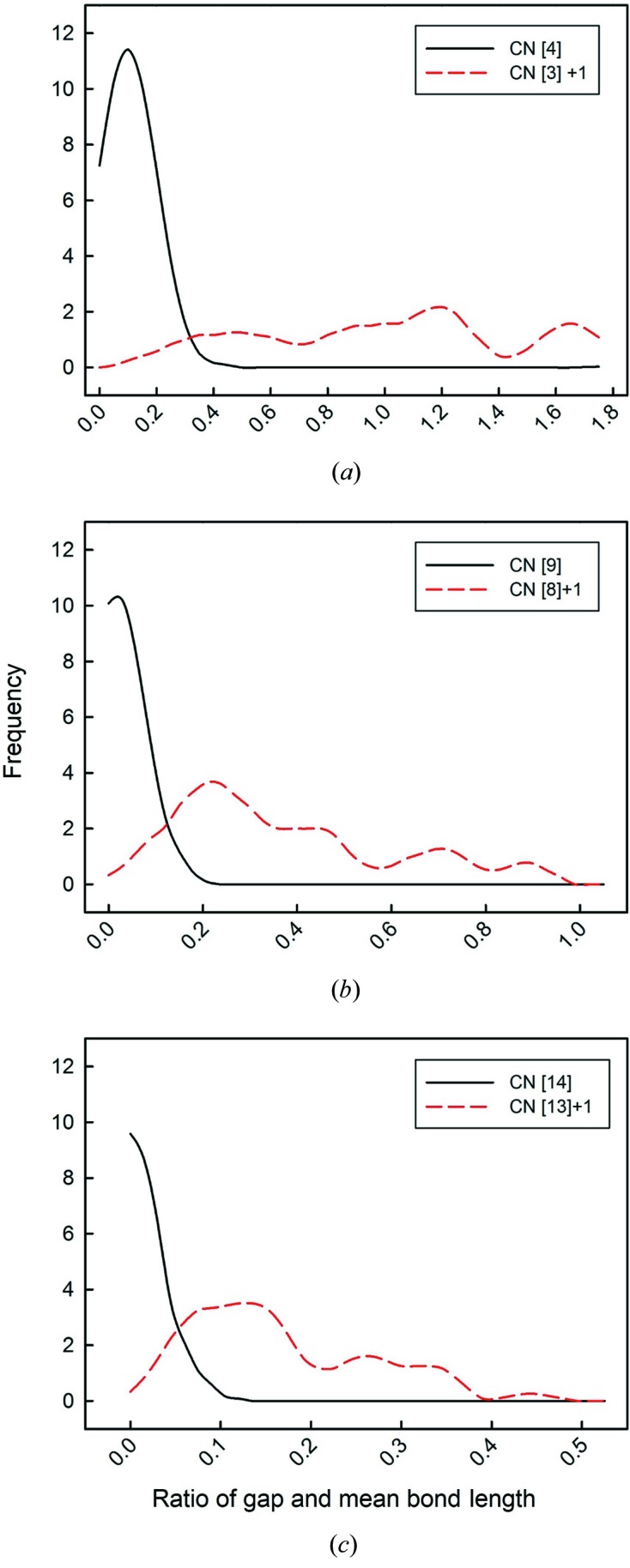
Ratio of the gap between the (*a*) third- and fourth-, (*b*) eighth- and ninth-, (*c*) thirteenth and fourteenth-shortest interatomic distance and the mean bond length of the (*a*) three, (*b*) eight, (*c*) thirteen shortest interatomic distances for coordination numbers (*a*) [3] and [4], (*b*) [8] and [9] and (*c*) [13] and [14]. Ions used are (*a*) Li^+^, Be^2+^, B^3+^, Na^+^ and S^6+^; (*b*) Na^+^, Ca^2+^, Y^3+^, Te^4+^, La^3+^; (*c*) K^+^, Rb^+^, Cs^+^, Ba^2+^. The lack of overlap between the two distributions of Fig. 2 ▸(*a*) suggests that the fourth shortest distance for ions described as [3] is non-bonding. In Figs. 2 ▸(*b*) and (*c*), the increasing overlap between the two distributions shows that the determination of the exact coordination number for larger coordination polyhedra is more ambiguous. Sample sizes are (*a*) *n*_[3] + 1_ = 41 and *n*_[4]_ = 58 coordination polyhedra; (*b*) *n*_[8] + 1_ = 50 and *n*_[9]_ = 50 coordination polyhedra; (*c*) *n*_[13] + 1_ = 50 and *n*_[14]_ = 47 coordination polyhedra.

We examined the bond-length distributions for [3] + 1 (the latter being the fourth-shortest distance) and [4]-coordinated Li^+^, Be^2+^, B^3+^, Na^+^ and S^6+^ to obtain a sense of the gap between the three shortest distances and the fourth distance for the coordination numbers [3] and [4]. Fig. 2 ▸(*a*) shows the ratio of the gap between the third- and fourth-shortest distances, and the mean bond length for the three shortest distances: (*l*_4_ − *l*_3_)/[(*l*_1_ + *l*_2_ + *l*_3_)/3] as a kernel density plot.^1^ For the coordination polyhedra we defined as [4], we observe a regular distribution with a mean value of 0.104 and minimum and maximum values of 0.000 and 0.333, respectively. For [3] + 1, the mean ratio is 1.056 and the minimum and maximum values are 0.198 and 2.521, respectively. The distributions of the data are strikingly different, and for [3]- and [4]-coordinated Li^+^, Be^2+^, B^3+^, Na^+^ and S^6+^ suggest that the fourth-longest interatomic distance can be considered as bonded if (*l*_4_ − *l*_3_)/[(*l*_1_ + *l*_2_ + *l*_3_)/3] < 0.333 (Fig. 2 ▸ *a*). We give analogous data for coordination numbers [8] + 1 and [9] in Fig. 2 ▸(*b*) and [13] + 1 and [14] in Fig. 2 ▸(*c*); in Fig. 2 ▸(*b*), we see a slight increase in the overlap between the distributions for [8] + 1 and [9], a trend that continues for [13] + 1 and [14] in Fig. 2 ▸(*c*). Fig. 2 ▸ shows that, for higher coordination numbers, the determination of the longest bond is somewhat more ambiguous, but still fairly reliable in most cases.

### 3.3. Large coordination numbers   

What large coordination numbers can we encounter in crystal structures? In terms of oxygen-based structures, Shannon (1976 ▸) lists ionic radii for coordination numbers up to [12], except for Rb^+^ which he lists up to [14]. In different types of crystals, higher coordination numbers are common, particularly in Frank–Kasper phases. For example, A15 intermetallic alloys of the form *A*_3_ *B* consist of [12]-coordinated *B* atoms and [14]-coordinated *A* atoms, and Laves *AB*_2_ intermetallic phases involve [12]- and [16]-coordinated atoms. In terms of oxygen-based structures, it is probable that only the alkali-metal and alkaline-earth-metal ions may have coordination numbers exceeding [12], and we will pay special attention to structures in which such coordination numbers may be possible. For these cations, attempting to determine the coordination number from a list of nearest-neighbour anions is usually ambiguous. Some cations form ill-defined coordination polyhedra and require special attention. To this effect, we describe the procedure whereby we assign the coordination polyhedron of a cation below (§4.2).

## 4. Methods   

The DVD-ROM version of the ICSD with *FindIt*, Version 2010-2, was used for data collection for all ions bonded to oxygen. The collection of bond-length data was done on the basis of coordination polyhedra for all cations of the periodic table. A set of structures containing each ion pair of interest was accumulated for each cation. In these structure sets, only the coordination polyhedra of the cation of interest were evaluated. The bonds in each coordination polyhedron were calculated and individually examined to ensure that only suitable entries were included. Valid coordination polyhedra were not discarded due to problems elsewhere in the structure that have no effect on the coordination polyhedron of interest.

### 4.1. Selection criteria   

The following criteria were used to select structure refinements:

(1) publication date ≥ 1975;

(2) *R*_1_ ≤ 0.06;

(3) the site of interest is fully occupied by the cation;

(4) all bonds involve ions at fully occupied sites;

(5) the cation and anion sites of interest show no positional disorder;

(6) crystallographic data were measured at ambient conditions;

(7) no data from powder, electron or synchrotron diffraction were included.

### 4.2. Determination of coordination polyhedra   

The following guidelines were used to decide on the coordination polyhedron (and thus coordination number) of each cation treated. For most cations, the first few points were sufficient for a clear determination of the coordination number:

(1) The cation is bonded only to O^2−^.

(2) In general, we assumed that all cation–anion bonds are shorter than the shortest cation–cation distance for the coordination number of interest.^2^

(3) The ordered list of distances was examined for a hiatus in the increasing distances.

(4) We examined the effect of different cation-coordination numbers on the anion coordination.

(5) We compared the bond lengths with and without potential bonds to the data already gathered for the cation to see if the behaviour resembled that of one coordination number more than another.

(6) After ∼ 10% of the structures had been processed for a specific ion pair, we examined the files for different coordination numbers of the same cation for potential trends and inconsistencies.

(7) We examined the chemical formula for the presence of unrefined H atoms. This was mostly relevant in locating weak bonds between the cation of interest and the O atom of an (H_2_O) group. Where H atoms were not located in the refinement, and such bonds seemed plausible, the data were discarded.

(8) We plotted the structure to obtain a visual sense of any ambiguity.

(9) Very sparingly, we used bond-valence curves to determine whether the inclusion or omission of bonds gave better bond-valence sums. However, little weight was given to this method because (1) it is circular, and (2) some published bond-valence parameters were doubtful at the time of this evaluation.

We plotted the bond-length frequency distributions for all ion configurations to identify obvious outliers that originated from (1) gross errors in the refinement, (2) errors in the ICSD entry, and/or (3) collection errors on our part. Arbitrary (often dynamic) limits were set for the lower and upper tails of the distribution of each ion configuration, whereby every entry with at least one bond below the former or above the latter was either (1) verified and confirmed in the original publication, or (2) discarded as an error. In particular, minerals may show considerable chemical zoning within individual grains and also significant grain-to-grain compositional differences, depending on the details of paragenesis. Where this is the case, there may be significant differences between the actual composition of the crystal and that assumed for the crystal used to collect the X-ray intensity data. Thus, errors regarding site occupancy may occur (unless the composition of that grain is analyzed by electron- and ion-microprobe), leading to apparently anomalous bond lengths. For entries with abundant data (*e.g.* Si^4+^ with over 10 500 bond distances), the entries were verified in terms of increasing bond lengths for the lower cut-off, and decreasing bond lengths for the upper cut-off, until a reliable series of entries were confirmed with those bond lengths. For configurations with very little data (∼ 3 or less coordination polyhedra), all entries were verified.

Taking into account the large amount of crystal structures examined, it was generally safer to discard a doubtful entry than it was useful to have its bond-length information included with reliable data, except where there was a paucity of bond lengths for the cation and coordination number(s) of interest.

Gagné & Hawthorne (2015 ▸) found that the agreement with the valence-sum rule of Brown (2002 ▸) is better when including the longer bonds in higher coordination polyhedra. As we shall see later, this is the case for the alkali and alkaline-earth metals, and also for configurations involving lone-pair-stereoactive cations (manuscript in preparation). Thus, long bonds obtained from the collection procedure described above were included.

This procedure resulted in 55 configurations involving 4859 coordination polyhedra and 38 594 individual bond lengths for the alkali metals, and 29 configurations involving 3038 coordination polyhedra and 24 487 individual bond lengths for the alkaline-earth metals. We make our complete dataset available for future more detailed work.

## 5. Shape of the bond-length distributions   

Bond-length distributions commonly resemble a positively skewed Gaussian distribution. The shape originates from the variation in Born repulsion and Coulomb attraction as a function of interatomic distance. Two useful statistical measures used to describe the shape of these distribution are skewness and kurtosis. Skewness is a measure of the asymmetry of the distribution about its mean, and can be positive (as in Fig. 1 ▸ for ^[6]^Na^+^) or negative. Kurtosis is a measure of the distribution of data between the peak and the tails of the distribution: a high kurtosis indicates that the distribution has a sharper maximum and larger tails, and a low kurtosis indicates that the distribution has a rounder maximum and smaller tails. Thus, important data that we derive from the bond-length distributions determined here are *mean bond length*, *skewness* and *kurtosis*. Deviations from this typical shape are frequent, and can be the result of structural and/or electronic effects that result in emergent bond-length constraints. Hence, we can gain insight into the reasons underlying the bonding behaviour of atoms from a visual inspection of their bond-length distributions, *e.g.* the familiar (4 + 2) bimodal distribution of bond lengths for octahedrally coordinated Cu^2+^ from the Jahn–Teller effect (Jahn & Teller, 1937 ▸), associated with the degenerate electronic ground state of a *d* ^9^ metal in a holosymmetric octahedral field.

## 6. Effect of sampling   

### 6.1. Sample size   

**Figure 3:**
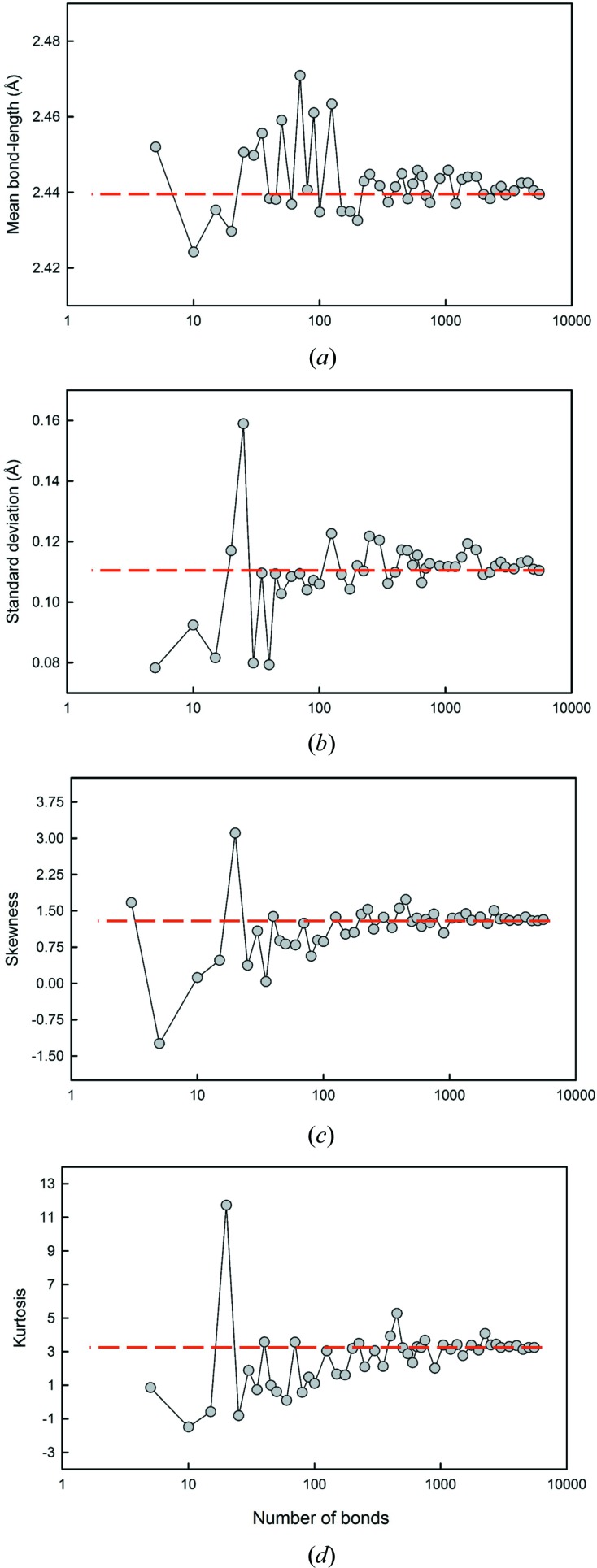
The effect of sample size on (*a*) mean bond length, (*b*) standard deviation of the mean bond length, (*c*) skewness and (*d*) kurtosis. The dashed line shows the value for the parent distribution.

A critical issue involved in the calculation of mean bond length, skewness and kurtosis is whether the sample size (number of bond lengths) is sufficiently large to ensure a representative distribution. We examined this issue using the data of Fig. 1 ▸, calculating the mean bond length, skewness and kurtosis for many different sample sizes and examining the values as a function of sample size compared with the values for the parent distribution (mean bond length = 2.441 with a standard deviation of 0.112 Å; skewness = 1.32, kurtosis = 3.25). The results are shown in Fig. 3 ▸. In the range 5500–1000 bonds, the values of mean bond length (Fig. 3 ▸ *a*) and its standard deviation (Fig. 3 ▸ *b*), skewness (Fig. 3 ▸ *c*) and kurtosis (Fig. 3 ▸ *d*) varied between 2.437–2.444 and 0.109–0.119 Å, 1.23–1.51 and 2.75–4.08, respectively; in the range 1000–100, the values of mean bond length and its standard deviation, skewness and kurtosis varied between 2.433–2.446 and 0.104–0.122, 1.02–1.73 and 1.60–5.28, respectively; below 100, the values of skewness and kurtosis varied from 2.424–2.471 and 0.078–0.159 Å, from −1.24 to 3.11 and from −1.49 to 11.72, respectively. Similar results were obtained (scaled by a factor of 6) by using polyhedra rather than individual bond lengths. As a result of these large variations, we do not list skewness and kurtosis values for sample sizes of less than 100 bonds in this work, and we note that the values for sample sizes up to 1000 bonds may be associated with non-negligible error. We do list mean bond lengths as this is important information for ongoing work on these materials, but we emphasize that the values listed may be adversely affected by small sample size.

### 6.2. The effect of outliers   

Values of skewness and kurtosis are very sensitive to the presence of outliers. For example, our dataset for ^[7]^Cs^+^ (Fig. S1*aq* of the supporting information) contains two coordination polyhedra with bonds longer than 3.75 Å, while the longest bond for the eight other polyhedra varies in the range 3.3–3.5 Å. Calculating the skewness and kurtosis with and without the two polyhedra with bonds longer than 3.75 Å, we obtain values of skewness and kurtosis of 1.51 and 3.04 (with), and 0.62 and −0.31 (without), respectively. Careful evaluation of the structure and of the probable longest bond (see §3.2) has greatly reduced the adverse effect of outliers on skewness and kurtosis in our analyses, but one must be careful of this issue as a single errant data point for a confirmed structure can change the values considerably.

### 6.3. Non-random sampling   

**Figure 4:**
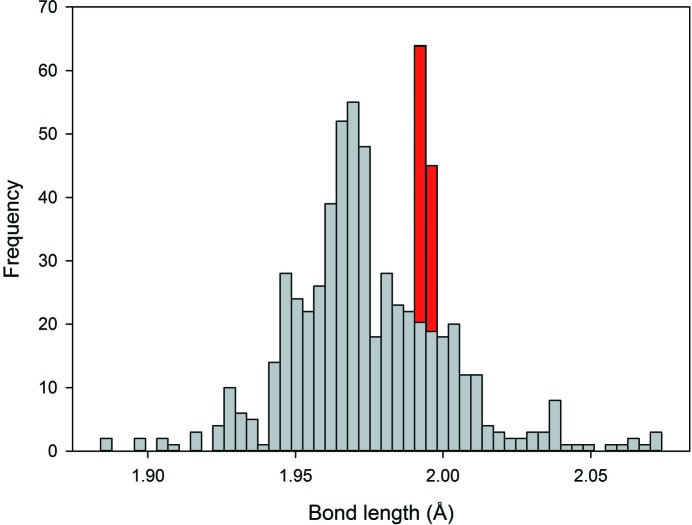
Bond-length distribution for ^[6]^Cr^3+^ bonded to O^2−^ (*n* = 624). A spike of data is observed at 1.99 Å due to extensive work done on the spinel structure.

Another issue that can affect skewness and kurtosis is the occurrence of spikes in the distribution of bond lengths due to extensive work on specific structure types. A prominent example is shown in Fig. 4 ▸: the bond-length distribution for ^[6]^Cr^3+^. There is a fairly typical distribution except for a prominent spike at 1.99 Å where ∼ 70 distances lie above the trend of the general distribution. Examination of the data shows that these distances originate from Lenaz *et al.* (2004 ▸); these authors refined structures from the solid solution (Mg,Fe^2+^)Cr_2_^3+^O_4_ with the spinel structure, and these contributed 11 structures in which the Cr^3+^—O^2−^ distances are symmetry-constrained to be identical in each structure, providing 66 distances of ∼ 1.99 Å and accounting for the spike in the distribution of Fig. 4 ▸.

Thus, §§6.1–6.3 [Sec sec6.2] [Sec sec6.3] emphasize that the numerical values for skewness and kurtosis must be interpreted with care. While the presence of trends in skewness and kurtosis gives us structural information, the absence of such trends may be due to sampling issues.

## 7. Results for the alkali metals   

**Figure 5:**
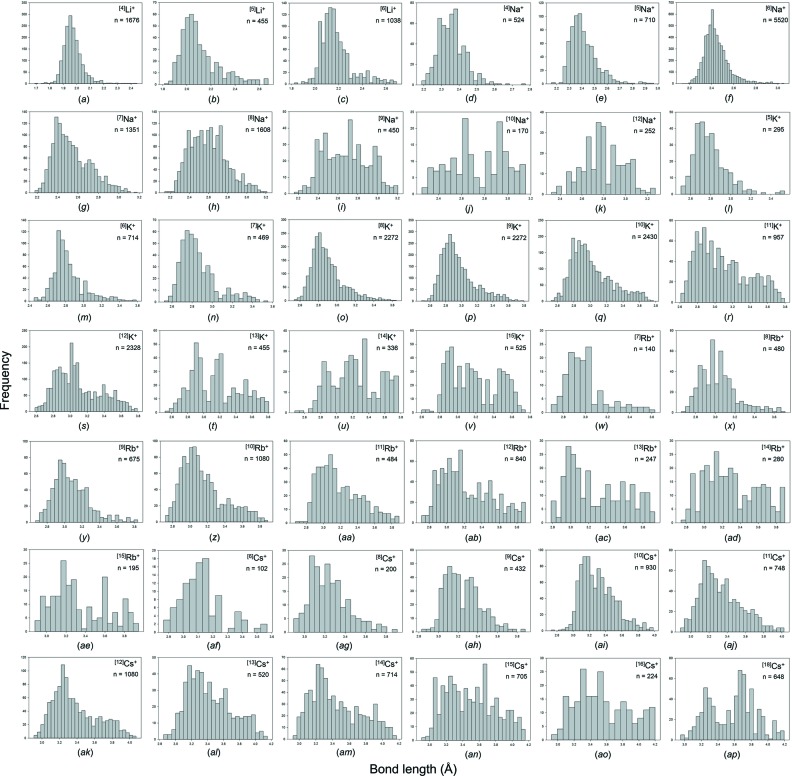
Bond-length distributions for the configurations of the alkali-metal ions bonded to O^2−^ with a sample size of 100+ bonds: (*a*) ^[4]^Li^+^, (*b*) ^[5]^Li^+^, (*c*) ^[6]^Li^+^, (*d*) ^[4]^Na^+^, (*e*) ^[5]^Na^+^, (*f*) ^[6]^Na^+^, (*g*) ^[7]^Na^+^, (*h*) ^[8]^Na^+^, (*i*) ^[9]^Na^+^, (*j*) ^[10]^Na^+^, (*k*) ^[12]^Na^+^, (*l*) ^[5]^K^+^, (*m*) ^[6]^K^+^, (*n*) ^[7]^K^+^, (*o*) ^[8]^K^+^, (*p*) ^[9]^K^+^, (*q*) ^[10]^K^+^, (*r*) ^[11]^K^+^, (*s*) ^[12]^K^+^, (*t*) ^[13]^K^+^, (*u*) ^[14]^K^+^, (*v*) ^[15]^K^+^, (*w*) ^[7]^Rb^+^, (*x*) ^[8]^Rb^+^, (*y*) ^[9]^Rb^+^, (*z*) ^[10]^Rb^+^, (*aa*) ^[11]^Rb^+^, (*ab*) ^[12]^Rb^+^, (*ac*) ^[13]^Rb^+^, (*ad*) ^[14]^Rb^+^, (*ae*) ^[15]^Rb^+^, (*af*) ^[6]^Cs^+^, (*ag*) ^[8]^Cs^+^, (*ah*) ^[9]^Cs^+^, (*ai*) ^[10]^Cs^+^, (*aj*) ^[11]^Cs^+^, (*ak*) ^[12]^Cs^+^, (*al*) ^[13]^Cs^+^, (*am*) ^[14]^Cs^+^, (*an*) ^[15]^Cs^+^, (*ao*) ^[16]^Cs^+^, (*ap*) ^[18]^Cs^+^.

Our collection and filtering criteria resulted in a combined sample size of 38 594 bonds and 4859 coordination polyhedra. Table 1 ▸ gives the 55 observed configurations, the mean bond length and standard deviation, the minimum and maximum bond length (and range), the skewness and kurtosis (where justified by sample size), and the number of bonds and coordination polyhedra for the five common alkali-metal ions. Fig. S1 gives all the bond-length distributions for the alkali metals; those with adequate sample sizes (as discussed above) are shown in Fig. 5 ▸. An important issue is the reliability of the data at the limits of its distribution, *i.e.* at the lowest and highest observed coordination numbers for each ion, and below we examine the data at the lower and upper limits of these distributions.

### 7.1. Observed coordination numbers   

**Figure 6:**
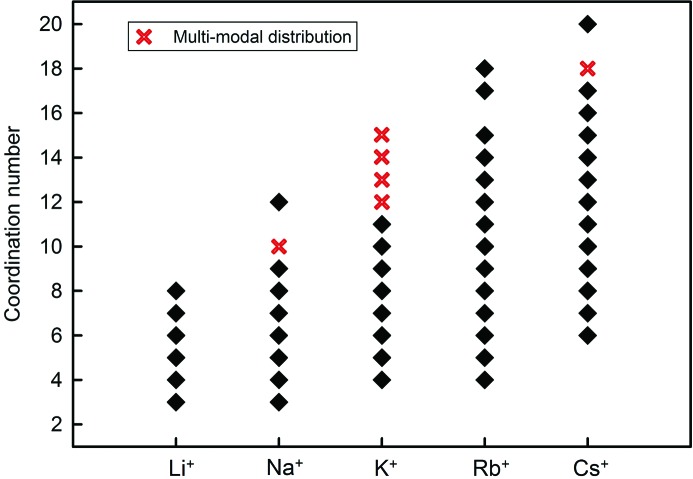
Observed coordination numbers for the alkali-metal ions. Multi-modal distributions are identified.

Fig. 6 ▸ shows the variation of the coordination numbers for the alkali-metal ions. The minimum coordination number increases from [3] for Li^+^ and Na^+^ to [4] for K^+^ and Rb^+^ to [6] for Cs^+^, and there is a corresponding increase in the maximum coordination number from [8] to [20]. The number of coordination numbers also increases along the series, from 6 for Li^+^ to 15 for Rb^+^ and Cs^+^.

#### 7.1.1. Li^+^   

Li^+^ has six coordination numbers from [3] to [8], with a strong preference for coordination number [4] (*n* = 419 coordination polyhedra). ^[3]^Li^+^ is observed in only four coordination polyhedra in four structures: Li_2_Yb_5_O_4_(BO_3_)_3_ (Jubera *et al.*, 2001 ▸); KLiO (Sabrowsky, Mertens & Thimm, 1985 ▸); RbLiO (Sabrowsky & Vogt, 1987 ▸); LiBa(B_9_O_15_) (Pushcharovskii *et al.*, 2002 ▸). The observed mean bond lengths are 1.958, 1.891, 1.915 and 1.888 Å, respectively, and the incident bond-valence sums (using the parameters of Gagné & Hawthorne, 2015 ▸) are 0.744, 0.825, 0.797 and 0.829 v.u., respectively. The displacement parameters for ^[3]^Li^+^ in Li_2_Yb_5_O_4_(BO_3_)_3_ are an order of magnitude larger than those for Yb^3+^ and twice that of ^[4]^Li^+^. Similarly, in LiBa(B_9_O_15_), *U*_eq_ for ^[3]^Li^+^ is ∼ 8 times *U*_eq_ for both the ^[3]^B^3+^ and ^[4]^B^3+^ cations (and the O^2−^ anions). These results suggest significant (dynamic or static) positional disorder. However, in KLiO, *U*_eq_ for ^[3]^Li^+^ is similar to the *U*_eq_ values for K^+^ and O^2−^, and there is no reason to question the [3]-coordination of Li^+^ in this structure.

^[7]^Li^+^ was found in only two coordination polyhedra (from two different structures) and ^[8]^Li^+^ was found in only one coordination polyhedron. ^[8]^Li^+^ was reported in Rb_6_LiPr_11_Cl_16_[SeO_3_]_12_ (Lipp & Schleid, 2006 ▸) with eight symmetrically equivalent bonds of length 2.513 Å giving an incident bond-valence sum of 0.835 v.u. with the parameters of Gagné & Hawthorne (2015 ▸). The *U*_eq_ value is five times that of its nearest-neighbour anions, all of which are equivalent, and twice that of the Rb^+^ cations in the structure. ^[7]^Li^+^ has been reported in LiGd_6_O_5_(BO_3_)_3_ (Chaminade *et al.*, 1999 ▸) and Li_2_(Mg,Cu)Cu_2_[Si_2_O_6_]_2_ (Horiuchi *et al.*, 1997 ▸), with mean bond lengths of 2.337 and 2.325 Å and incident bond-valence sums of 1.021 and 1.067 v.u., respectively. Moreover, there are no significant hiati in the list of increasing bond distances for each structure. In LiGd_6_O_5_(BO_3_)_3_, *U*_eq_ for Li^+^ is three–four times the values of the other atoms. In Li_2_(Mg,Cu)Cu_2_[Si_2_O_6_]_2_, *U*_eq_ for Li^+^ is five times the values of the other metal atoms and three times the values of the anions; moreover, the displacements for Li^+^ are very anisotropic. There is no alternative to Li^+^ in [7]- and [8]-coordination in these structures, but it is apparent from the very large *U*_eq_ values that Li^+^ is ‘rattling around’ in overly large holes in these structures (the cation is significantly displaced from the centre of the polyhedron). Although one might argue that the resultant observed mean bond lengths are thus anomalous, *U*_eq_ values are generally correlated with coordination number and it is difficult to refute the suggestion that what we observe for [7]- and [8]-coordinated Li^+^ is just a (non-linear) extrapolation of behaviour at lower coordination numbers. This issue is discussed further in §10.3.

#### 7.1.2. Na^+^   

Na^+^ is observed in nine coordinations, from [3] to [12] excluding [11]. ^[6]^Na^+^ is by far the most common coordination (*n* = 920), followed by ^[8]^Na^+^ (*n* = 201) and ^[7]^Na^+^ (*n* = 193). ^[3]^Na^+^ (*n* = 7) has a very small spread in bond lengths, from 2.214 to 2.367 Å, but this is a common feature we observe for all [3]-coordinated cations. The incident bond-valence sums are very low (0.70 v.u. on average); for example, in Na_6_CoO_3_ (Möller, 1998 ▸), the 〈^[3]^Na^+^—O^2−^〉 distance is 2.255 Å (the shortest of the eight coordination polyhedra), and the incident bond-valence sum is 0.804 v.u. In this structure, the coordination of ^[3]^Na^+^ is triangular with a 〈O^2−^—Na^+^—O^2−^〉 angle of 117.2° and a slight pyramidal character, and the next-nearest O^2−^ anion is at 3.57 Å, far beyond any significant bond-valence interaction. Thus the occurrence of Na^+^ in [3]-coordination seems established. For [12] coordination, the central cation and the coordinating anions generally show similar values of *U*_eq_ with no anomalous disorder.

#### 7.1.3. K^+^   

K^+^ is observed in 12 different coordinations, from [4] to [15], with a preference for coordination numbers 9 (*n* = 308), 8 (*n* = 284) and 10 (*n* = 243). There are 24 coordination polyhedra for ^[4]^K and the central cation and the coordinating anions generally show similar values of *U*_eq_ with no anomalous disorder. The grand mean incident bond-valence for ^[4]^K is 0.77 v.u. with a range of 0.53–0.92 v.u.

#### 7.1.4. Rb^+^   

Rb^+^ is observed in 14 coordinations from [4] to [18], excluding [16]. It favourably adopts coordination number 10 (*n* = 108), followed by coordination numbers 9 (*n* = 75) and 8 (*n* = 60). Despite little data, ^[4]^Rb^+^ (*n* = 12) shows a typical distribution and the constituent atoms are well behaved in most of the structure refinements. The grand mean incident bond valence for ^[4]^Rb^+^ is 0.57 v.u. with a range of 0.49–0.65 v.u. The following coordination numbers [15] (*n* = 13), [17] (*n* = 1) and [18] (*n* = 5) have mean incident bond-valence sums of 0.947, 1.002 and 0.912 v.u., respectively. The *U*_eq_ values tend to be very high for the central cations in these structures. For example, in Rb_5_VONb_14_O_38_ (Haddad & Jouini, 1997 ▸), *U*_eq_ of ^[18]^Rb^+^ is 5–7 times those of the coordinating anions; in Rb_10_Ta_29.2_O_78_ (Fallon & Gatehouse, 1980 ▸), *U*_eq_ of ^[18]^Rb^+^ is 15–20 times those of the coordinating anions; in Rb_2_V_3_P_4_O_17_ (Lii *et al.*, 1990 ▸), *U*_eq_ of ^[17]^Rb^+^ is ∼ 3 times those of the coordinating anions.

#### 7.1.5. Cs^+^   

Cs^+^ is observed in 14 coordinations, from [6] to [20] excluding [19], with a preference for coordination numbers 10 (*n* = 94) and 12 (*n* = 90). ^[6]^Cs^+^ (*n* = 18) and ^[7]^Cs^+^ (*n* = 10) show regular distributions despite limited data, and the central cations and the coordinating anions generally show similar values of *U*_eq_ with no anomalous disorder. For ^[20]^Cs^+^, there are distances 3.210 ×8 and 4.064 ×12 Å with incident bond-valence sums of 0.886 v.u. for [8] and 1.029 v.u. for [20], and the *U*_eq_ value of the central Cs^+^ is ∼ 5 times those of the coordinating anions.

### 7.2. Grand mean bond length as a function of coordination number   

**Figure 7:**
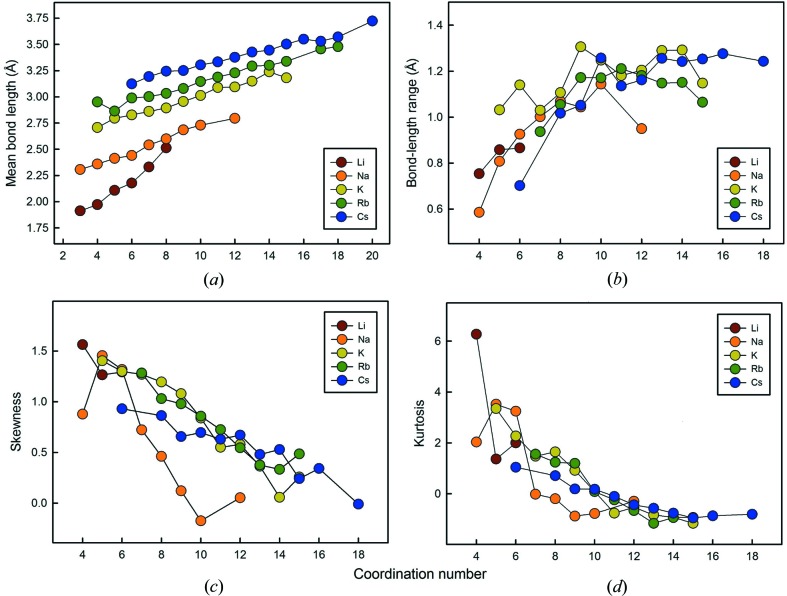
Values of (*a*) grand mean bond length, (*b*) bond-length range, (*c*) skewness and (*d*) kurtosis for the different coordination numbers of the alkali-metal ions

Fig. 7 ▸(*a*) shows the variation in mean bond length as a function of coordination number, and is given for all configurations regardless of sample size. The correlation is positive and is surprisingly regular. Minor anomalies (*e.g.* ^[5]^Rb^+^) can be attributed to a small sample size. We note that the slope of the variation for each ion in Fig. 7 ▸(*a*) decreases slightly with the increasing size of the cations.

### 7.3. Range in bond length as a function of coordination number   

Fig. 7 ▸(*b*) shows the variation in the range of bond lengths as a function of coordination number. As the ranges are strongly dependent on sample size, we omitted data for configurations of less than 100 bonds. Although this criterion is sufficient to remove major outliers, the value for ^[12]^Na^+^ is ∼ 0.22 Å, smaller than expected from the general trend of Fig. 7 ▸(*b*); the reason for this difference is not clear. There is a strong non-linear trend in Fig. 7 ▸(*b*); the range in bond lengths is positively correlated with coordination number, and the range increases more rapidly at lower coordination numbers, and flattens out at higher coordination numbers.

### 7.4. Skewness and kurtosis as a function of coordination number   

Figs. 7 ▸(*c*) and (*d*) show skewness and kurtosis as a function of coordination number, respectively, for the five alkali-metal ions. As skewness and kurtosis are highly influenced by the amount of data used in their calculation, the graphs only show values for configurations with 100 bonds or more, as discussed above. Fig. 7 ▸(*c*) shows a more-or-less linear decrease in skewness with increasing coordination number. Na^+^ shows a lower skewness for coordination [4] than would be expected from the trend, for reasons that are not clear, and the rate of decrease in kurtosis with increasing coordination number is greater than that for the other alkali-metal ions which show a surprisingly consistent trend (considering the sensitivity of skewness to sample size and outliers). Fig. 7 ▸(*d*) shows a well developed trend of non-linear decrease in kurtosis as a function of coordination number for all the alkali-metal ions. The trend for Na^+^ is again somewhat less consistent than for the other ions, and the shape is similar to that exhibited for skewness (Fig. 7 ▸ *c*).

## 8. Results for the alkaline-earth metals   

**Figure 8:**
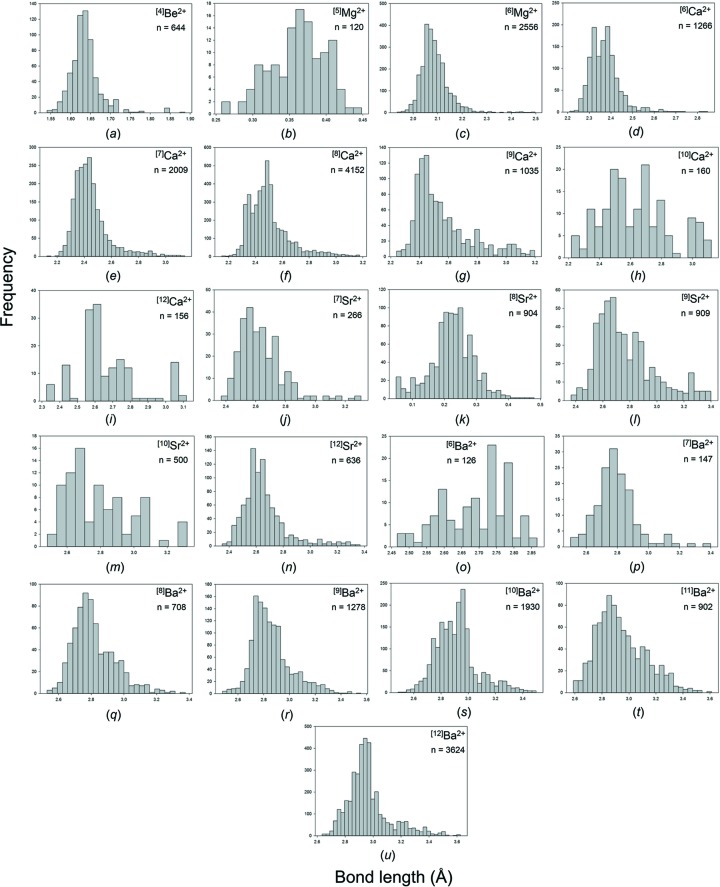
Bond-length distributions for the configurations of the alkaline-earth-metal ions bonded to O^2−^ with a sample size of 100+ bonds: (*a*) ^[4]^Be^2+^, (*b*) ^[5]^Mg^2+^, (*c*) ^[6]^Mg^2+^, (*d*) ^[6]^Ca^2+^, (*e*) ^[7]^Ca^2+^, (*f*) ^[8]^Ca^2+^, (*g*) ^[9]^Ca^2+^, (*h*) ^[10]^Ca^2+^, (*i*) ^[12]^Ca^2+^, (*j*) ^[7]^Sr^2+^, (*k*) ^[8]^Sr^2+^, (*l*) ^[9]^Sr^2+^, (*m*) ^[10]^Sr^2+^, (*n*) ^[12]^Sr^2+^, (*o*) ^[6]^Ba^2+^, (*p*) ^[7]^Ba^2+^, (*q*) ^[8]^Ba^2+^, (*r*) ^[9]^Ba^2+^, (*s*) ^[10]^Ba^2+^, (*t*) ^[11]^Ba^2+^, (*u*) ^[12]^Ba^2+^.

Our collection and filtering criteria resulted in a combined sample size of 24 487 bonds and 3038 coordination polyhedra. Table 2 ▸ gives the 29 observed configurations, the mean bond length and standard deviation, the minimum and maximum bond length (and range), the skewness and kurtosis (where justified by sample size), and the number of bonds and coordination polyhedra for the five common alkaline-earth-metal ions. Fig. S2 gives all the bond-length distributions for the alkaline-earth metals; those with adequate sample sizes (as discussed above) are shown in Fig. 8 ▸. These ions are found in slightly more than half the number of configurations observed for the alkali metals (55), primarily because these ions are not observed in coordinations higher than [12], with the exception of Ba^2+^ (observed as [13] and [14]).

### 8.1. Observed coordination numbers   

**Figure 9:**
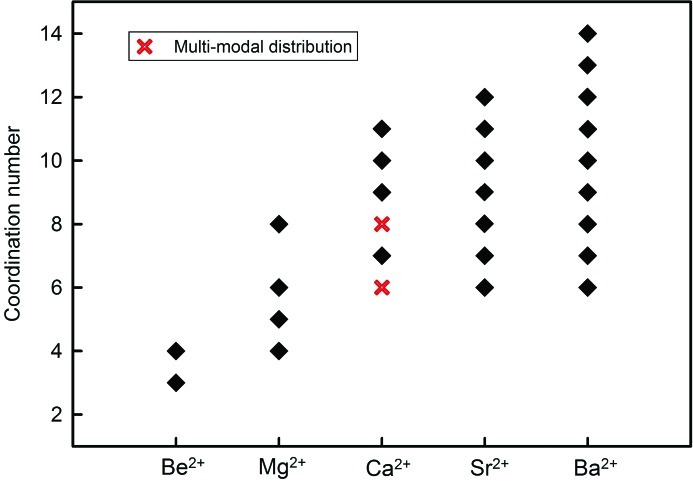
Observed coordination numbers for the alkaline-earth-metal ions. Multi-modal distributions are identified.

Fig. 9 ▸ shows the variation of the coordination numbers for the alkaline-earth-metal ions. The minimum and maximum coordination numbers increase with size, [3] and [4] for Be^2+^, [4] and [8] for Mg^2+^, [6] and [12] for Ca^2+^ and Sr^2+^, and [6] and [14] for Ba^2+^. The number of coordination numbers also increases along the series, from 2 for Be^2+^ to 4 for Mg^2+^, 7 for Ca^2+^, 8 for Sr^2+^ and 9 for Ba^2+^.

#### 8.1.1. Be^2+^   

Be^2+^ occurs in two coordination numbers, with a clear preference for coordination number [4] (*n* = 161) compared with its 3-coordinated form ^[3]^Be^2+^ (*n* = 8). However, the coordination of [3] seems well established for Be. For example, Leoni *et al.* (2005 ▸) report the structure of Ba_3_[Be_5_O_8_] with a Be atom [3]-coordinated at distances of 1.545 (9) and 1.561 (5) ×2 Å with well behaved *U*_eq_ values for both the central cation and the coordinating anions, and an incident bond-valence sum of 1.96 v.u.

#### 8.1.2. Mg^2+^   

Mg^2+^ occurs in four different coordination numbers from [4] to [8], excluding [7]. ^[6]^Mg^2+^ is by far the most common coordination with *n* = 426 coordination polyhedra. ^[4]^Mg^2+^ (*n* = 12) has an apparent bimodal distribution, which is clearly an artifact of a small number of bond lengths that show little spread. However, the coordination [4] must be considered as well established for Mg^2+^ as structure refinements show well behaved cations and anions. The grand mean incident bond valence for ^[8]^Mg is 1.93 v.u. with a range of 1.78–2.20 v.u. The structures containing ^[8]^Mg^2+^ look well refined, although *U*_eq_ values are usually somewhat higher than those of the anions, suggesting significant displacement. This effect was also suggested by Shannon & Rossman (1992 ▸) with regard to deviations of measured and calculated dielectric constants for ^[8]^Mg^2+^.

#### 8.1.3. Ca^2+^   

Ca^2+^ occurs in seven different coordinations from [6] to [12] with a preference for coordination number [8] (*n* = 519). [6]-coordinated Ca^2+^ is well established (*n* = 211). The lowest observed 〈^[6]^Ca^2+^—O〉 distance is 2.254 (4) Å in Ba_3_CaRu_2_O_9_ (Wilkens & Müller-Buschbaum, 1991 ▸); this seems very short compared with the grand 〈^[6]^Ca^2+^—O〉 distance of 2.371 (69) Å (Table 2 ▸), but there is no reason to reject this value based on the structure refinement. There are few examples of the higher coordination-numbers: ^[10]^Ca (*n* = 16), ^[11]^Ca (*n* = 7) and ^[12]^Ca (*n* = 13). The grand mean incident bond valence for ^[12]^Ca is 2.03 v.u. with a range of 1.43–2.50 v.u. The lowest values are for Ca_3_Zn_4_Ti_16_O_38_ (Gatehouse & Grey, 1983 ▸) and CaMg_2_Al_16_O_27_ (Iyi *et al.*, 1995 ▸). Ca_3_Zn_4_Ti_16_O_38_ has Ca—O distances 2.762 (5) ×6 and 2.792 (5) ×6 Å with a mean value of 2.777 Å and a displacement parameter that is 3–10 times those of the other atoms in the structure. CaMg_2_Al_16_O_27_ has Ca—O distances 2.773 (8) ×6 and 2.799 (21) ×6 Å with a mean value of 2.786 Å and a displacement parameter that is 2–6 times those of the other atoms in the structure. Thus the low incident bond-valence sums are associated with central cations that show very large displacements. The highest values are for CaCu_3_Ge_4_O_12_ (Ozaki *et al.*, 1977 ▸) and CaCu_3_Ge_4_O_12_ (Chenevas *et al.*, 1975 ▸). CaCu_3_Ge_4_O_12_ has Ca—O distances of 2.549 (4) ×12 Å and a displacement parameter that is similar to those of the other atoms in the structure. CaCu_3_Ge_4_O_12_ has Ca—O distances of 2.562 (3) ×12 Å and a displacement parameter that is similar to those of the other atoms in the structure. Thus the high incident bond-valence sums are associated with central cations that show displacements similar to those of the other atoms in the structure.

#### 8.1.4. Sr^2+^   

Sr^2+^ occurs in 7 different coordinations from [6] to [12] with a preference for coordination numbers [8] (*n* = 113) and [9] (*n* = 101). An unusual coordination for Sr occurs in the crystal structure of β-Sr_10_Ga_6_O_19_ (Kahlenburg, 2002 ▸), where one of 11 crystallographically distinct Sr atoms has bond lengths of 2.425 (8) ×2, 2.471 (9) ×2 and 3.350 (9) ×2 Å; is Sr^2+^ [4]- or [6]-coordinated? For [4]-coordination, the four anions do not form a tetrahedron and the Sr^2+^ cation lies between the two closest anions with an O^2−^—Sr^2+^—O^2−^ angle of 175.4°. For [6]-coordination, the Sr^2+^ cation lies almost in the plane of four of the anions that form a face of the polyhedron. The sums of the incident bond valences are ∼ 1.5 v.u. for both [4]- and [6]-coordination. The two next-nearest anions are 3.519 Å away from the central Sr^2+^, but there are two Ga^3+^ atoms at 3.410 Å. Thus, the coordination in this particular case is uncertain, and the coordination polyhedron was omitted from the data for Sr^2+^.

#### 8.1.5. Ba^2+^   

Ba^2+^ occurs in nine different coordinations from [6] to [14] with a clear preference for coordination number [12] (*n* = 302). There are few examples of coordination numbers > [12]: ^[13]^Ba^2+^ (*n* = 6) and ^[14]^Ba^2+^ (*n* = 1). For ^[13]^Ba^2+^, the sums of the incident bond valences are in the range 1.85–2.37 v.u., and for ^[14]^Ba^2+^ the sum of the incident bond valences is 1.98 v.u.

### 8.2. Grand mean bond length as a function of coordination number   

**Figure 10:**
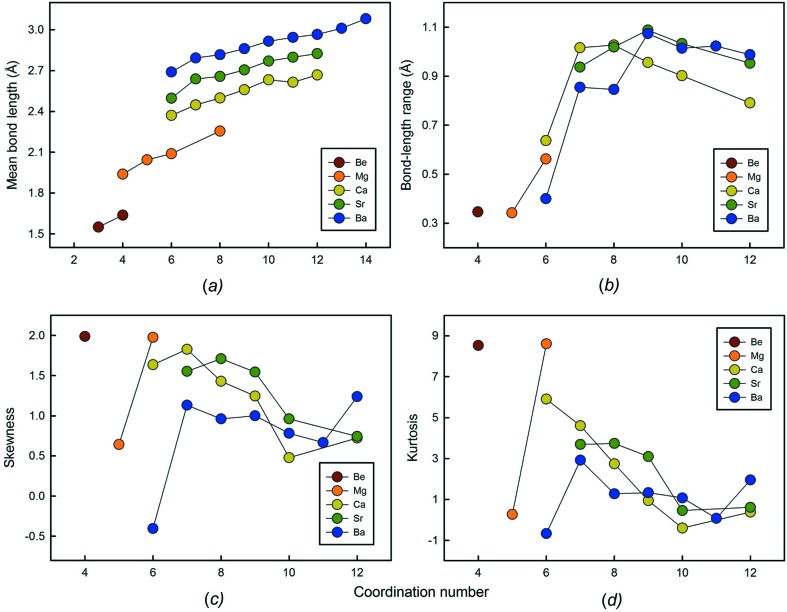
Values of (*a*) grand mean bond length, (*b*) bond-length range, (*c*) skewness and (*d*) kurtosis for the different coordination numbers of the alkaline-earth-metal ions.

Fig. 10 ▸(*a*) shows the variation in mean bond length as a function of coordination number, and is given for all configurations regardless of sample size. The correlation is positive and very regular. The slope of the variation for each ion of Fig. 10 ▸(*a*) decreases slightly with the increasing size of the cations, although less than is the case for the alkali-metal ions.

### 8.3. Range in bond length as a function of coordination number   

Fig. 10 ▸(*b*) shows the variation in the range of bond lengths as a function of coordination number, omitting data for configurations of less than 100 bonds. There is a strong non-linear trend in the data; the range increases more rapidly at lower coordination numbers, [4]–[7], but levels out at ∼ [8] (∼ 0.95 ± 0.1 Å) and is fairly constant thereafter, aside from a decrease for the higher coordination of Ca^2+^ (probably due to few data). The initial increase in range for smaller coordination numbers is steeper for the alkaline-earth metals than for the alkali metals (Fig. 7 ▸ *b*) with mean slopes of 0.37 for Mg^2+^, Ca^2+^ and Ba^2+^ up to [7], compared with ∼ 0.15 for Na^+^ and Cs^+^ up to [8].

### 8.4. Skewness and kurtosis as a function of coordination number   

Figs. 10(*c*) and (*d*) summarize the variation of skewness and kurtosis as a function of coordination number for the common alkaline-earth-metal ions, respectively. There is a more-or-less linear decrease in skewness with increasing coordination number. Abnormally low values of skewness (and kurtosis) are obtained for the bond-length distributions of ^[5]^Mg^2+^ and ^[6]^Ba^2+^, but are likely due to insufficient data. The slopes of these graphs are generally steeper for the alkaline-earth metals than for the alkali metals, meaning that the progressive ‘flattening’ of the distributions described for the alkali metals is less developed for the alkaline-earth metals.

There is a systematic decrease in kurtosis with coordination number for the alkaline-earth-metal ions (Fig. 10 ▸ *d*), with the same anomalies as for skewness (Fig. 10 ▸ *c*). The resemblance of the trends for skewness and kurtosis is striking, as is the case for the alkali-metal ions (Figs. 7 ▸ *c* and *d*). The values of skewness and kurtosis arising from the bond-length distributions of each family are strongly correlated (alkali metals: *R* ^2^ = 0.75; alkaline-earth metals: *R* ^2^ = 0.74).

## 9. General discussion of bond-length distributions   

### 9.1. Skewness   

As noted in §5, bond-length distributions are expected to resemble a positively skewed Gaussian distribution, and this shape originates from the variation in Born repulsion and Coulomb attraction as a function of interatomic distance. In particular, as the coordination number of a cation increases, the mean bond-length increases and the slope of the Born repulsion curve decreases as the mean cation–anion distances increase. This makes the potential energy curve more symmetrical about the mean bond length and hence the skewness of the distribution of bond lengths should decrease with increasing coordination number. Inspection of Figs. 7 ▸(*c*) and 10 ▸(*c*) shows that this is generally the case for the alkali metals and the alkaline-earth metals.

### 9.2. Kurtosis   

**Figure 11:**
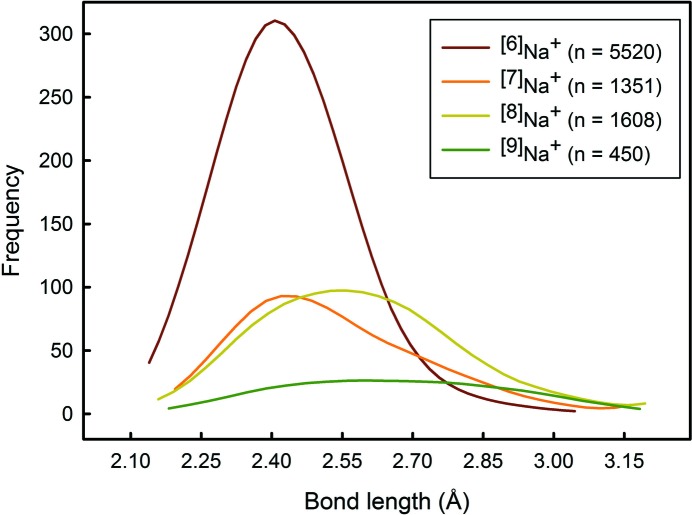
Kernel-density estimation of the bond-length distributions of the coordination numbers [6] to [9] for Na^+^. The plot shows that changes in skewness and kurtosis values are primarily due to a change in the shape of the maximum of the bond-length distribution.

As noted above, kurtosis is a measure of the distribution of data between the peak and the tails of the distribution: a high kurtosis indicates that the distribution has a sharper maximum and larger tails, and a low kurtosis indicates that the distribution has a rounder maximum and smaller tails. Fig. 11 ▸ shows a kernel-density estimation of the bond-length distributions of coordination numbers [6] to [9] for Na^+^. This example shows decreases in both kurtosis and skewness from coordination [6] to [9]: kurtosis values are 1.317, 0.723, 0.461 and 0.121, and skewness values 3.246, −0.020, −0.197 and −0.879, respectively. Fig. 11 ▸ shows that the major contributor to kurtosis is the shape of the maximum of the distribution, rather than the length of the tail, as the tails and the minimum and maximum bond lengths are fairly similar for all coordination numbers, but the flattening of the distribution is very notable with increasing coordination number. Figs. 7 ▸(*d*) and 10 ▸(*d*) show that kurtosis decreases with increasing coordination number for the alkali metals and the alkaline-earth metals; note that the main deviations from this trend are the same as those for the skewness plots, reinforcing the suggestion that small sample size may be the cause of these deviations.

For low coordination numbers, a change in bond valence corresponds to a relatively small change in bond length because of the steepness of the bond-valence—bond-length curve at short bond lengths. For high coordination numbers, a change in bond valence corresponds to a large change in bond length because of the shallowness of the bond-valence—bond-length curve at long bond lengths. Thus ions with low coordination numbers show a lower range in bond lengths, whereas ions with high coordination numbers show a much greater range in bond lengths (Figs. 7 ▸ *b* and 10 ▸ *b*), leading to much flatter distributions (*i.e.* with lower kurtosis) at higher coordination numbers.

### 9.3. Multi-modal distributions   

**Figure 12:**
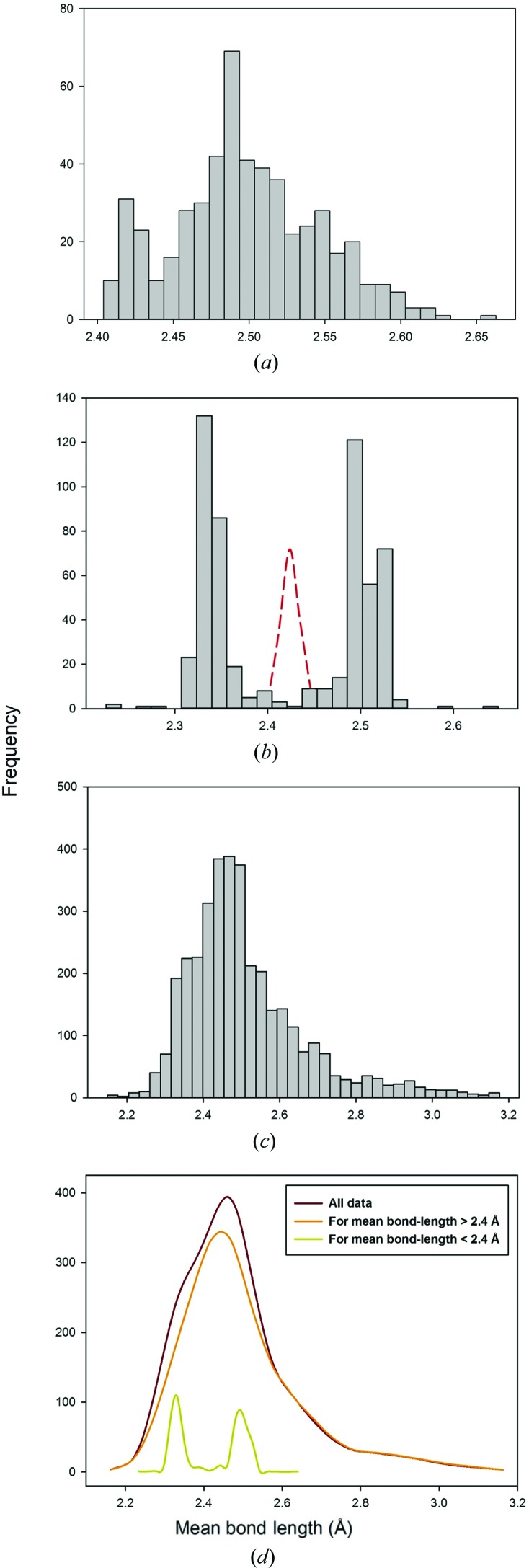
Mean bond-length distribution of ^[8]^Ca^2+^ bonded to O^2−^ (*a*). Bond-length distribution for ^[8]^Ca^2+^ bonded to O^2−^ are shown for configurations with a mean bond length (*b*) less or equal to 2.44 Å (superimposed with its mean bond-length kernel-density estimation; *n* = 568), and (*c*) greater than 2.44 Å (*n* = 3584). The effect of the former on the aggregate bond-length distribution is shown *via* kernel-density estimation (*d*).

Several cations show multi-modal distributions of their bond lengths. The most prominent bimodal distribution is for ^[8]^Ca^2+^ and shows in both the distribution of individual bond lengths (Fig. 8 ▸ *f*) and mean bond lengths (Fig. 12*a* ▸). For the distribution of individual bond lengths, there is an intense maximum at 2.49 Å and a less intense maximum at 2.35 Å; for the distribution of mean bond lengths, there is an intense maximum at 2.49 Å and a less-intense maximum at 2.42 Å. Inspection of the data with 〈^[8]^Ca^2+^—O^2−^〉 less than 2.44 Å (79 polyhedra) shows that there are 33 garnet structures and 27 vesuvianite structures in this range. The distribution of individual ^[8]^Ca^2+^—O^2−^ distances from polyhedra with a mean bond length of 2.44 Å or less are shown in Fig. 12 ▸(*b*), and the distribution of individual ^[8]^Ca^2+^—O^2−^ distances from polyhedra with a mean bond length greater than 2.44 Å are shown in Fig. 12 ▸(*c*). Fig. 12 ▸(*b*) also shows the kernel density estimation of the mean bond-length distribution of its constituent polyhedra. Removal of the data of Fig. 12 ▸(*b*) from the overall distribution of ^[8]^Ca^2+^—O^2−^ distances gives a single-mode distribution (Fig. 12 ▸ *c*). The kernel density estimations for all three distributions are shown in Fig. 12 ▸(*d*), and indicate how the bimodal distribution of Fig. 8 ▸(*f*) arises. In the garnet and vesuvianite structures, the bond lengths are split into two equal populations, four larger than the mean and four smaller than the mean. As shown in Fig. 12 ▸(*d*), the population of larger distances merges with the overall distribution of bond lengths and does not materially alter its overall shape, whereas the population of smaller distances lies toward the lower edge of the overall distribution and gives rise to a shoulder on that distribution.

Multi-modal distributions are observed for the following configurations: ^[10]^Na^+^, ^[12]^K^+^, ^[13]^K^+^, ^[14]^K^+^, ^[15]^K^+^, ^[18]^Cs^+^, ^[6]^Ca^2+^ and ^[8]^Ca^2+^. Other configurations may show deviations from unimodal behaviour, although this is often unclear, possibly due to too few data and/or significant overlap: ^[4]^Na^+^, ^[15]^Rb^+^, ^[9]^Cs^+^, ^[10]^Cs^+^, ^[12]^Cs^+^, ^[10]^Ba^2+^. What the example of ^[8]^Ca^2+^ makes clear is that one must examine the effects of non-random sampling (by large numbers of data on a specific structure type) before ascribing such an effect to any crystal-chemical mechanism.

### 9.4. Bond valences   

**Figure 13:**
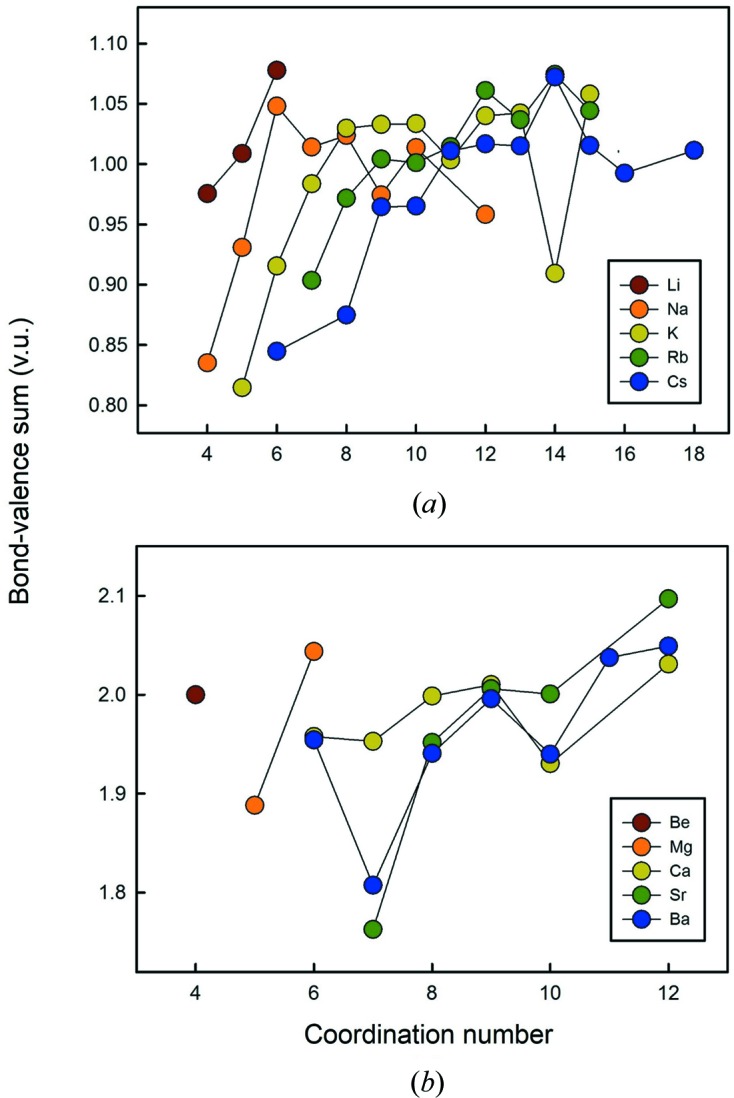
Bond-valence sum as a function of coordination number for (*a*) the alkali-metal and (*b*) alkaline-earth-metal ions using the parameters of Gagné & Hawthorne (2015 ▸).

Figs. 13 ▸(*a*) and (*b*) show the mean bond-valence sum as a function of coordination number, using the parameters of Gagné & Hawthorne (2015 ▸) for those alkali-metal and alkaline-earth metal configurations of 100 bonds or more. There is a general increase in mean bond-valence sum with increasing coordination number. This increase is much larger with smaller coordination numbers and tends to level off at larger (> [9]) coordination numbers. In the bond-valence model, the incident bond-valence sum for any ion is (approximately) equal to the ion valence, and there should be no correlation between incident bond-valence sum and coordination number of the ion. As indicated in Fig. 13 ▸, this is not the case. Moreover, this correlation is much more exaggerated when using the bond-valence parameters of other authors, as Gagné & Hawthorne (2015 ▸) added a coordination-based optimization factor in their method of derivation of bond-valence parameters. However, they were not able to eliminate the correlation between mean bond-valence sum and coordination number for all ions. Correlations of the type shown in Fig. 13 ▸ have been noted in the past, but have always been attributed to a limitation in the form of the bond-valence equation used. In particular, it has been suggested (*e.g.* Wander *et al.*, 2015 ▸) that the exponential equation of Brown & Altermatt (1985 ▸) has too shallow a slope at short bond distances and too steep a slope at long bond distances, resulting in short (long) bonds seeming weaker (stronger) than they are. However, this effect is not limited to the equation of Brown & Altermatt (1985 ▸). Gagné & Hawthorne (2015 ▸) tested a series of two- and three-parameter equations, and found that any equation that gives a good fit to the data suffers from the same ‘curvature problem’ at short and long bond distances. Although the origin of this problem is not clear, this shows that the problem may not lay in the parameterization of the relation, but possibly in a breakdown of the model itself in structures with unusual coordinations. This issue requires further investigating.

### 9.5. Ions with coordination numbers possibly exceeding [12]   

Gagné & Hawthorne (2015 ▸) give bond-valence parameters for four ions to which they assign coordinations higher than [12]: K^+^, Rb^+^, Cs^+^ and Ba^2+^. Table 3 ▸ lists their values of the RMSD from the valence-sum rule obtained from their dataset for each ion and calculated using the equation of Brown & Altermatt (1985 ▸)
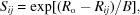
where *R_ij_* is the bond length between ions *i* and *j*, *S_ij_* is the corresponding bond valence, and *R*_o_ and *B* are the bond-valence parameters. Here, we derive new bond-valence parameters using a hard cut-off of 12 bonds for those configurations we observe in coordination numbers greater than [12]. These are also listed in Table 3 ▸ with their associated RMSD values. There are minor changes in the *R*_o_ parameter for K^+^ and Ba^2+^ (2.047 to 1.985, and 2.223 to 2.208 Å, respectively), but there are major differences for Rb^+^ and Cs^+^ (1.993 to 1.780 and 2.305 to 1.966 Å, respectively). In all cases, the *B* parameter increases to offset the decrease in *R*_o_: for K^+^, 0.398 to 0.425 Å; for Rb^+^ 0.478 to 0.577 Å; for Cs^+^ 0.411 to 0.561 Å; and for Ba^2+^ 0.406 to 0.417 Å. It is no surprise that *R*_o_ decreases as some of the longer distances are left out, as Gagné & Hawthorne (2015 ▸) showed that *R*_o_ is correlated with the mean bond length of an ion (as well as other physical observables such as ionization energy). To compensate for the decrease in *R*_o_, the *B* parameter adjusts to higher values.

We may assess whether or not interatomic distances greater than those used for [12] coordination are valid in the following ways: (1) verify if the mean bond lengths and bond-valence parameters still follow established trends (*e.g.* with ionization energy) without the longer bonds; (2) verify the valence-sum rule for the bond-valence parameters obtained with and without the cut-off indirectly *via* the anion bond-valence sums for a set of structures containing these ions; (3) run computational simulations as to whether electron density is observed between the ions at longer distances. Option (3) has no experimental verification and is not considered here.

(1) We plotted mean bond length as a function of *R*_o_ for the five alkali-metal ions for the values including and excluding bonds with a hard cut-off of [12]. Including the bonds, *R* ^2^ = 0.94, whereas excluding these bonds, *R* ^2^ drops to 0.79. Plotting *R*_o_/(mean bond length) against ionization energy (Gagné & Hawthorne, 2015 ▸), *R* ^2^ (inclusive) = 0.35, whereas *R* ^2^ (exclusive) = 0.01.

(2) We compared the bond-valence sums of the anions for 19 structures containing K^+^, Rb^+^, Cs^+^ and Ba^2+^, which we originally described in coordinations greater than [12], for the following two cases: (1) using the parameters of Gagné & Hawthorne (2015 ▸) and no cut-off, and (2) using new bond-valence parameters (above) that were derived with a cut-off of coordination [12], and doing the evaluation on the 19 structures using a cut-off of [12]. We find that both sets of parameters, used in the way they were derived, give exactly the same result for the anion bond-valence sums (RMSD = 0.128 v.u., or 6.4% from the nominal oxidation state).

Following (1) and (2) we conclude that the notion of counting bonds up to a maximum of 12 seems unjustified. We can find no strong argument against the occurrence of higher coordination numbers.

## 10. Mean bond-length distributions   

**Figure 14:**
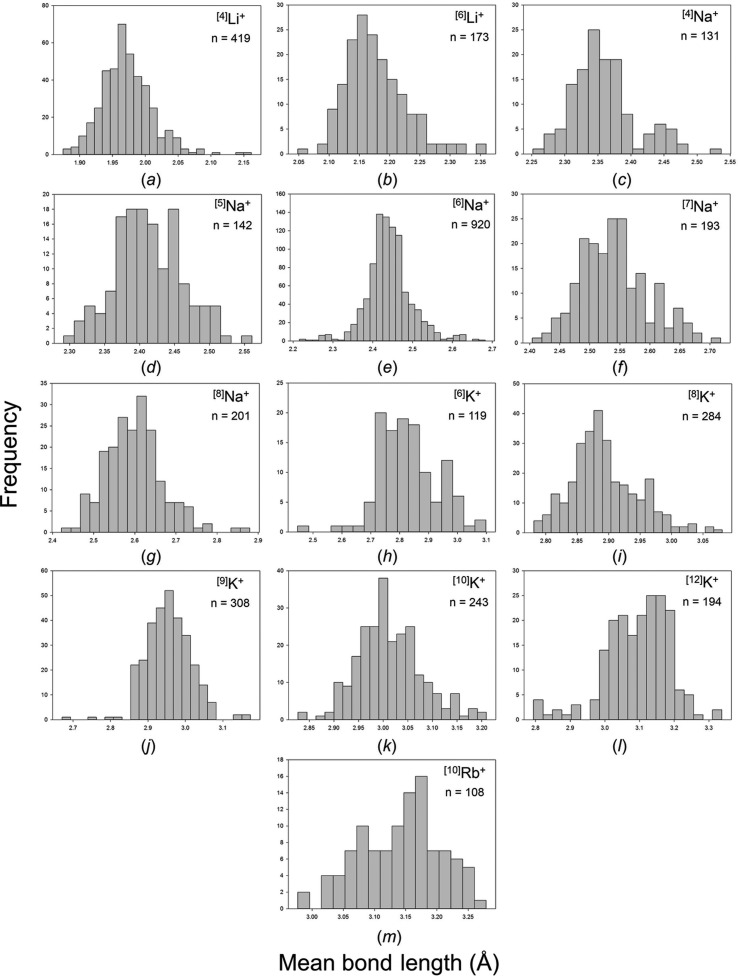
Mean bond-length distributions for the configurations of the alkali-metal ions bonded to O^2−^ with a sample size of 100+ coordination polyhedra: (*a*) ^[4]^Li^+^, (*b*) ^[6]^Li^+^, (*c*) ^[4]^Na^+^, (*d*) ^[5]^Na^+^, (*e*) ^[6]^Na^+^, (*f*) ^[7]^Na^+^, (*g*) ^[8]^Na^+^, (*h*) ^[6]^K^+^, (*i*) ^[8]^K^+^, (*j*) ^[9]^K^+^, (*k*) ^[10]^K^+^, (*l*) ^[12]^K^+^, (*m*) ^[10]^Rb^+^.

Figs. S3 and S4 give all the mean bond-length distributions for the alkali-metal and alkaline-earth-metal ions; those with adequate sample sizes (below) are shown in Figs. 14 ▸ and 15 ▸. Tables 4 ▸ and 5 ▸ give the grand mean bond length and standard deviation, the minimum and maximum mean bond length (and range), the skewness and kurtosis of each distribution (where justified by sample size) and the number of coordination polyhedra and coordination numbers for all configurations for the alkali and alkaline-earth metals. A minimum sample size was determined in the same way as above for ^[6]^Na^+^, less than which the values of skewness and kurtosis have little significance; this threshold was set to 100 coordination polyhedra and is relatively high due to the wide range of mean bond lengths observed for these families.

**Figure 15:**
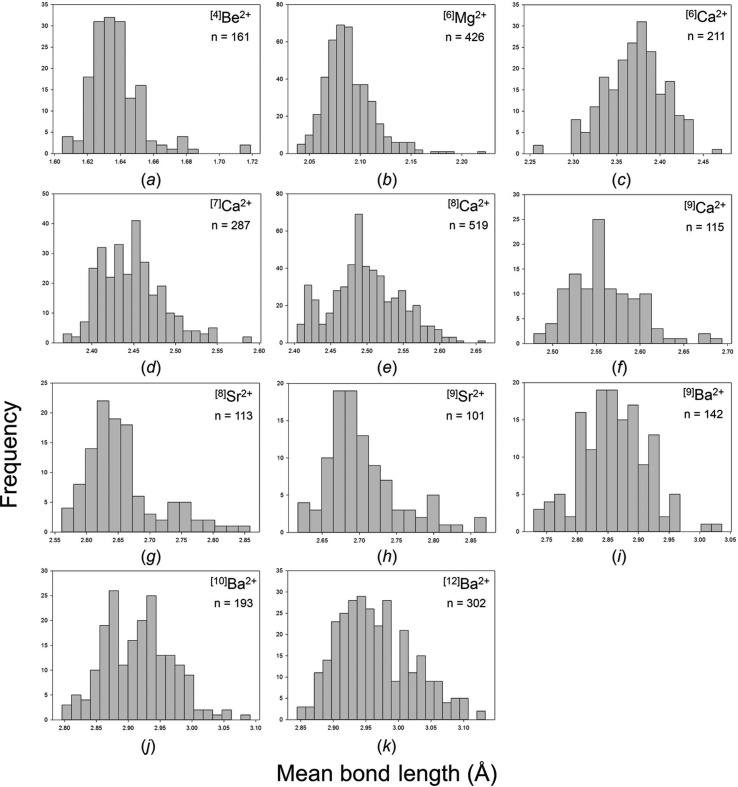
Mean bond-length distributions for the configurations of the alkaline-earth-metal ions bonded to O^2−^ with a sample size of 100+ coordination polyhedra: (*a*) ^[4]^Be^2+^, (*b*) ^[6]^Mg^2+^, (*c*) ^[6]^Ca^2+^, (*d*) ^[7]^Ca^2+^, (*e*) ^[8]^Ca^2+^, (*f*) ^[9]^Ca^2+^, (*g*) ^[8]^Sr^2+^, (*h*) ^[9]^Sr^2+^, (*i*) ^[9]^Ba^2+^, (*j*) ^[10]^Ba^2+^, (*k*) ^[12]^Ba^2+^.

The average range of mean bond lengths for the 55 configurations of the alkali metals is 0.308 Å, and is 0.217 Å for the 29 configurations of the alkaline-earth metals. The largest range is observed for ^[6]^K^+^, from 2.447 to 3.099 Å. The distribution for ^[4]^Be^2+^ (Fig. 15 ▸ *a*) shows a feature that is of importance in examining bond-length distributions: there is a notable outlier with two 〈^[4]^Be^2+^—O〉 distances at ∼ 1.717 Å. These values are for sørensenite, ideally Na_4_Sn^4+^[Be_2_Si_6_O_18_](H_2_O)_2_ (Metcalf-Johansen & Hazell, 1976 ▸), which was assumed to have the ideal composition in the structure study. However, inspection of the chemical analyses listed by Semenov *et al.* (1965 ▸) shows that the formulae depart significantly from the ideal stoichiometry used to interpret the structure results. In many pegmatite minerals, Be^2+^ is commonly partly substituted by Li^+^ or Al^3+^, both of which are larger than Be^2+^ (Shannon, 1976 ▸) and this may be what has happened here. Of course, this is speculation, but emphasizes the importance of electron- and ion-microprobe analysis of the specific crystal used to collect X-ray intensity data for structure analysis of minerals.

### 10.1. The effect of distortion   

#### 10.1.1. The distortion theorem   

**Figure 16:**
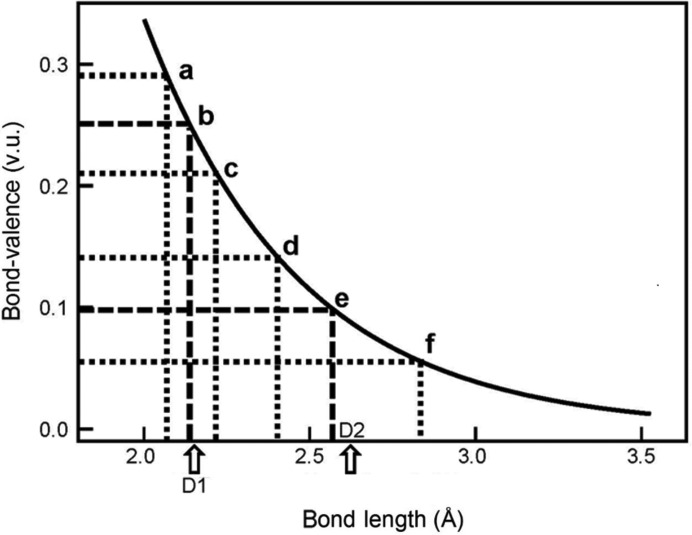
Bond-valence bond-length curve for Na^+^. The exponential shape leads to the distortion theorem of the bond-valence model.

The distortion theorem states that for any ion, lengthening some bonds and shortening others, keeping the bond-valence sum the same, will always increase the mean bond length due to the exponential nature of the relation (Brown, 2002 ▸). Here we use the following definition of bond-length distortion from the mean value in a polyhedron
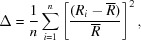
where 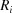 is the length of bond *i*, 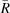 is the mean bond length and the summation is taken over the *n* bonds of the polyhedron. This expression was designated the quadratic elongation by Robinson *et al.* (1971 ▸) and distortion by Brown & Shannon (1973 ▸), and was used by many previous authors as a measure of distortion as it is the standard deviation of the mean bond length in a specific polyhedron, *i.e.* the measure of dispersion of the individual bond lengths. The distortion is shown graphically in Fig. 16 ▸ with the bond-valence curve for Na^+^. For [4]-coordinated Na^+^, the mean bond valence is 0.25 v.u., marked by the dashed line intersecting the bond-valence curve at point *b* (Fig. 16 ▸). If half the bonds shorten and half the bonds lengthen to *a* and *c*, respectively, on the bond-valence curve, the mean bond length (marked by D1 in Fig. 16 ▸) increases slightly over the mean bond length for four equal bonds. For [10]-coordinated Na, the mean bond valence is 0.10 v.u., marked by the dashed line intersecting the bond-valence curve at point *e* (Fig. 16 ▸). If half the bonds shorten and half the bonds lengthen to *d* and *f*, respectively, on the bond-valence curve, the mean bond length (marked by D2 in Fig. 16 ▸) increases considerably more over the mean bond length for ten equal bonds.

**Figure 17:**
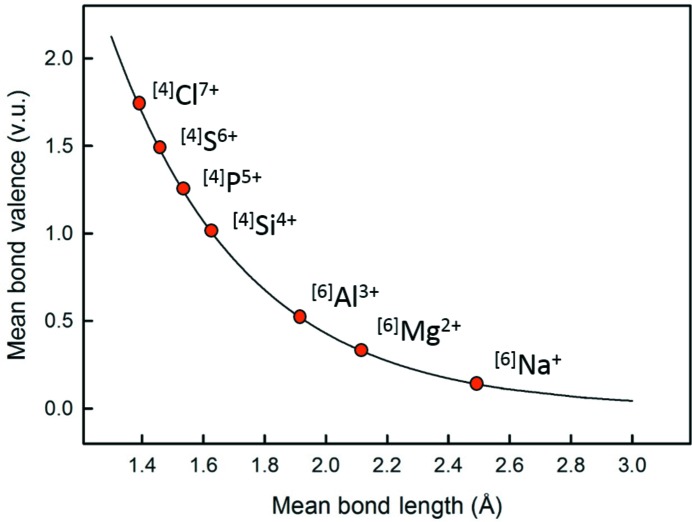
Bond-valence curve for the Na isoelectronic series, and ideal mean bond-valence and associated mean bond-length for the most common coordination number of each ion of the series.

**Figure 18:**
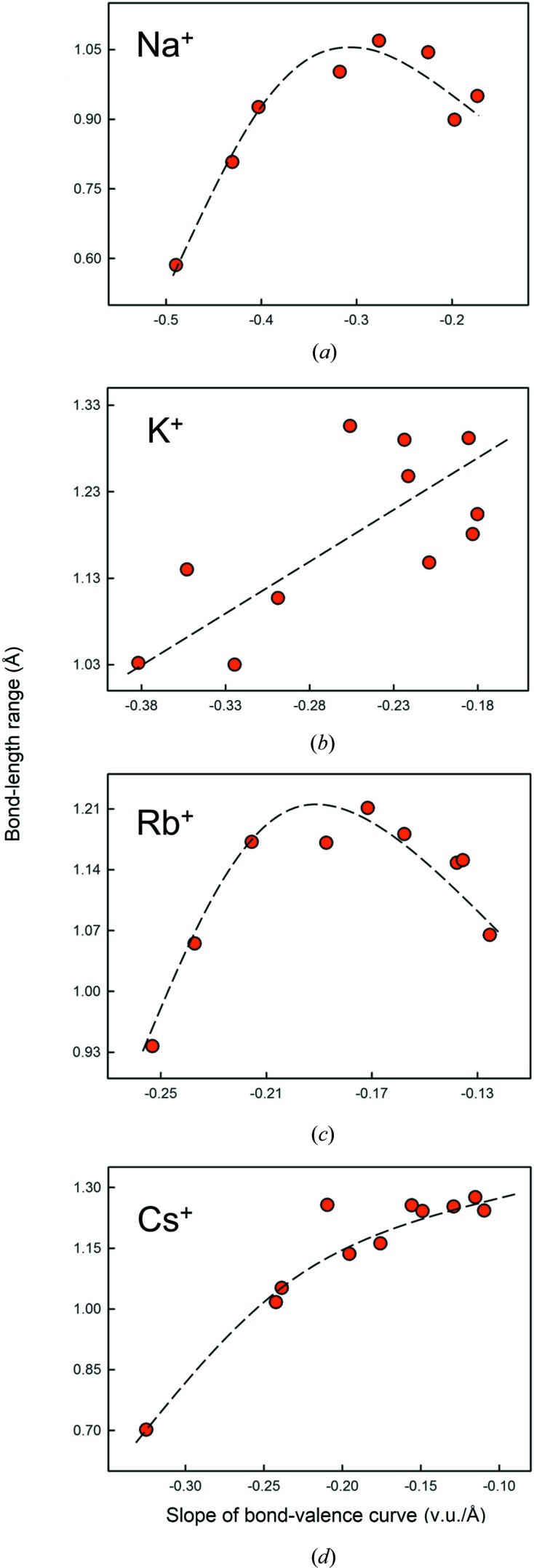
Variation in the observed bond-length ranges for the different coordination numbers of (*a*) Na^+^, (*b*) K^+^, (*c*) Rb^+^ and (*d*) Cs^+^ bonded to O^2−^ as a function of the slope of the bond-valence curve at the mean bond-length corresponding to those coordinations. The dashed lines are drawn as a guide to the eye (they are not least-squares fits to the data).

Secondly, Fig. 16 ▸ shows that the effect of the distortion theorem is greatly affected by the slope and curvature of the bond-valence–bond-length relation; if we use the same concept for points *a*, *b* and* c*, we see that the mean bond length changes very little, and much less relative to points *d*, *e* and *f*. Different ions have their mean bond length at different points (curvature) on this graph, and thus are affected differently by the distortion theorem. The best way to visualize this concept is by making use of a universal curve as described in Brown & Shannon (1973 ▸), where the relation for an isoelectronic series of ions can be described by the same bond-valence curve (with a slight decrease in fit compared with ion-based curves). In Fig. 17 ▸ we show the bond-valence curve for the Na isoelectronic series (*R*_o_ = 1.630, *B* = 0.438), and identify the ideal mean bond-valence and associated mean bond length for the most common coordination number of each ion of the series. We see that ions of lower charge, and with generally higher coordination numbers, occur on part of the bond-valence curve that is much more susceptible to higher bond-length distortion (*i.e.* with lower bond valences). Hence, the alkali metals are more strongly affected by distortion than the alkaline-earth metals, and so on. Figs. 18 ▸(*a*)–(*d*) show the variation in the range of bond lengths for different coordination numbers as a function of the slope of the bond-valence curve at the mean bond length corresponding to those coordination numbers for Na^+^, K^+^, Rb^+^ and Cs^+^. As predicted above, there is a positive correlation between range and slope for each cation; the trends are well developed for Na^+^, Rb^+^ and Cs^+^, despite a decrease at the highest coordination number for Na^+^ and Rb^+^ possibly due to fewer data, but is perturbed by considerable scatter for K^+^. As noted above, the distortion will also be correlated with the curvature (as well as the slope) of the bond-valence curve, but the slope and curvature are highly correlated and their effect is well represented by just the slope of the curve.

#### 10.1.2. Grand mean bond length   

**Figure 19:**
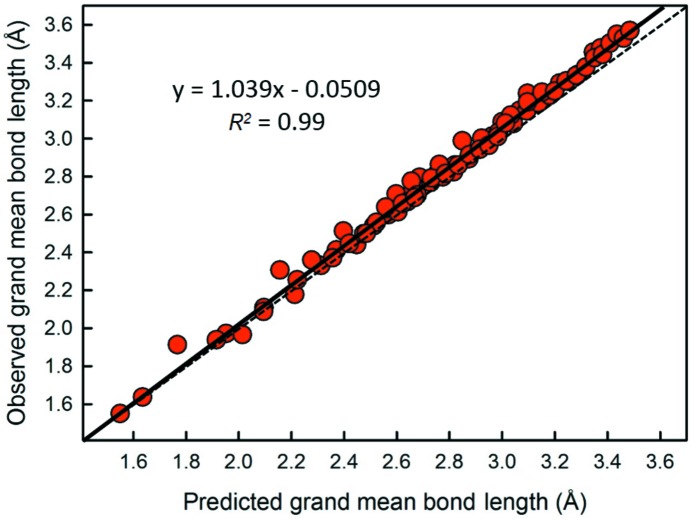
Observed *versus* predicted values of the grand mean bond length for all observed ion configurations of the alkali-metal and alkaline-earth-metal ions. The observed values of mean bond length are usually larger than the values predicted by the bond-valence curve for equidistant bonds. The dashed line is for *y* = *x*.

Using the bond-valence parameters of Gagné & Hawthorne (2015 ▸), we calculated the predicted grand mean bond length for all configurations of the alkali-metal and alkaline-earth-metal ions by converting the mean bond-valence (*i.e.* the Pauling bond strength) of the coordination polyhedron into a mean bond length, where all bonds of the idealized polyhedron have the same length. When comparing the observed grand mean bond length (Tables 4 ▸ and 5 ▸) to the calculated values, we obtain an overall difference of 2.5% for the alkali metals (1.2% when weighted by the number of coordination polyhedra), and 1.0% (0.8% weighted) for the alkaline-earth metals. As shown in Fig. 19 ▸, the predicted values closely follow the observed values, but are slightly larger and the difference increases with increasing mean bond length, in accordance with the idea that distortion of coordination polyhedra causes an increase in mean bond length, and that this effect should increase with increasing coordination number.

For any ion configuration we may calculate the maximum amount of distortion that is compatible with its observed bond-length distribution, using the bond-valence model and the minimum and maximum bond length observed for that configuration. We have done this for all configurations of the alkali-metal and alkaline-earth-metal ions, by using the minimum and maximum bond length observed for each configuration and distributing the other bond lengths in a way that maximizes distortion while satisfying the valence-sum rule. This procedure resulted in a mean potential distortion of 3.9% for the 55 alkali-metal configurations (4.6% when weighted by the number of coordination polyhedra), compared with 2.5% (1.2% weighted) for the observed values, and 2.8% (3.4% weighted) for the 29 alkaline-earth-metal configurations, compared with 1.0% (0.8% weighted) for the observed values. These calculations show that for most ion configurations, the observed ranges of bond lengths are significantly larger than is compatible with the observed distortions. Thus, in addition to distortion, other factors must also affect mean bond lengths.

#### 10.1.3. Mean bond length as a function of distortion   

**Figure 20:**
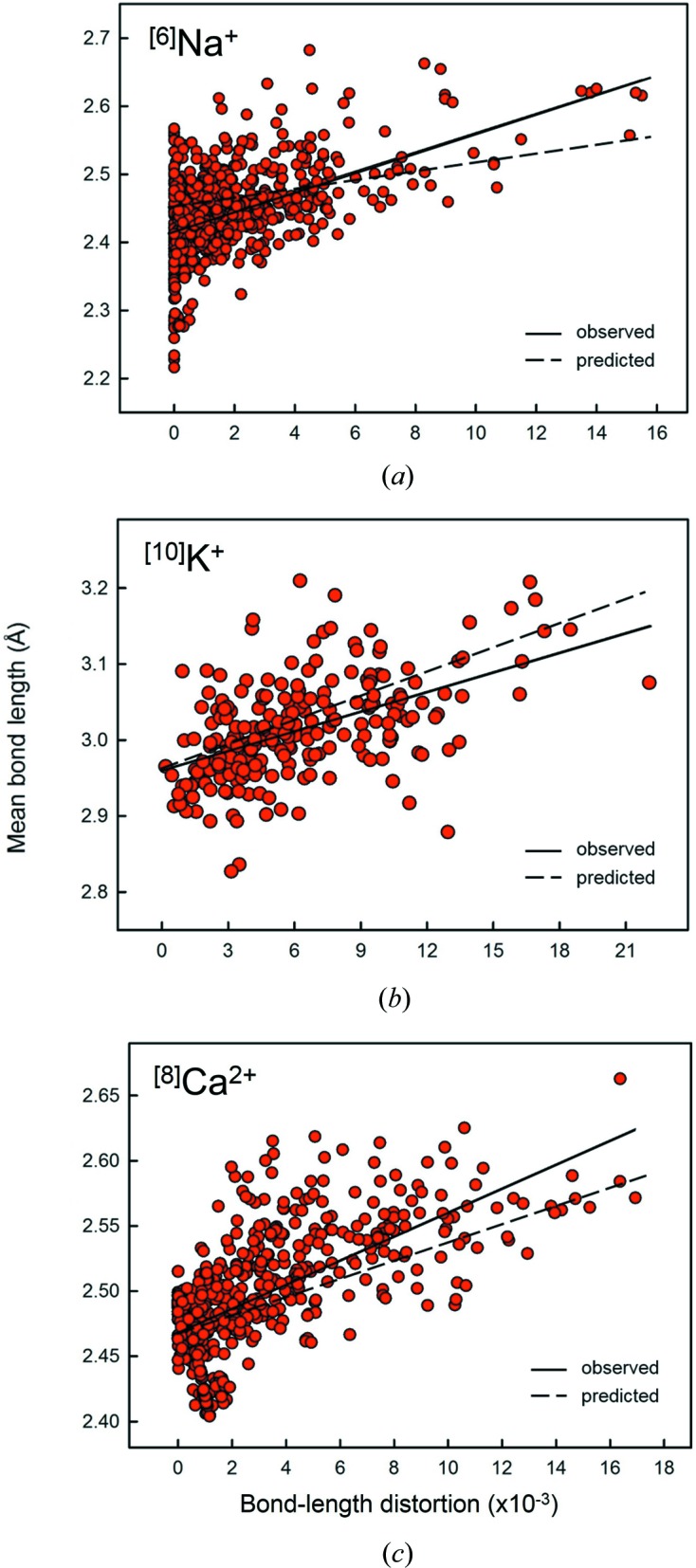
The effect of bond-length distortion on mean bond length for (*a*) ^[6]^Na^+^, (*b*) ^[10]^K^+^ and (*c*) ^[8]^Ca^2+^. The positive correlation indicates that distortion of the coordination polyhedron has a considerable effect on the mean bond length of the constituent polyhedron. The dashed line indicates the predicted effect of distortion on mean bond length for that ion configuration according to the bond-valence curve of the ion.

Fig. 20 ▸(*a*) shows the mean bond length for ^[6]^Na^+^—O^2−^ as a function of distortion (*n* = 920). There is a positive correlation between mean bond length and distortion, Δ, for reasons discussed above; the inclined solid line shows the result of a linear regression (*R* ^2^ = 0.263). The calculated curve for the effect that distortion has on mean bond length for ^[6]^Na^+^—O^2−^ is shown by the dashed line on Fig. 20 ▸(*a*). The difference between observed and predicted curve is partly due to the data near Δ = 0 (discussed below) which has a very large scatter. Additionally, we note that highly distorted configurations generally have poor agreement with the valence-sum rule, which leads to variability in the data above and below the predicted curve. However, highly distorted polyhedra for ^[6]^Na^+^ (with higher mean bond lengths) tend to have low bond-valence sums (∼ 0.8–0.9 v.u.), indicating that the mean bond lengths of these polyhedra are larger than predicted from the valence-sum rule and the form of the bond-valence curves.

A very prominent feature of Fig. 20 ▸(*a*) is the fact that the widest range of mean bond lengths occurs at zero distortion; 〈^[6]^Na^+^—O^2−^〉 varies from 2.216 to 2.567 Å at Δ = 0 alone, compared with 2.276 to 2.682 Å for all other data with Δ ≠ 0, and the range of observed mean bond lengths decreases as Δ increases. The total range in mean bond length is 0.466 Å; this may be compared with the predicted range that may be assigned to the effect of distortion (dashed line), 0.11 Å, as well as the observed range (solid line), 0.21 Å (Fig. 20 ▸ *a*).

We give the analogous bond-length distortion plots for the 55 configurations of the alkali-metal ions and 29 configurations of the alkaline-earth-metal ions in Figs. S5 and S6, respectively. We note that this concentration of data at Δ = 0 is evident for Li^+^ in [4]- and [6]-coordination, Na^+^ in [4]- and [6]- coordination and K^+^ in [6]-coordination, and occurs more subtly for Na^+^ in [5]- and [8]-coordination as well as K^+^ in [8]- [9] and [12]-coordination. For the alkaline-earth metals, this concentration of data at Δ = 0 occurs for Be^2+^ [4]-coordinated, Mg^2+^ [4]- and [6]-coordinated, Ca^2+^ [6]- and [12]-coordinated, Sr^2+^ [6]-coordinated and Ba^2+^ [12]-coordinated, and more subtly for Ca^2+^ [7]- and [8]-coordinated, Sr^2+^ [8]-coordinated and Ba^2+^ [8] and [10]-coordinated. In Fig. 20 ▸(*b*), we show the distortion plot of ^[10]^K^+^, which in contrast to that for ^[6]^Na^+^ (Fig. 20 ▸ *a*) shows no preference for Δ = 0. It is therefore interesting to see that the observed mean bond-length values (solid line) are generally lower than what we predict over the whole range of distortion (dashed line) because of the absence of the large scatter and amount of data near Δ = 0 that greatly affects the slope of the observed curve for ion configurations with significant scatter at Δ = 0. Fig. 20 ▸(*c*) shows ^[8]^Ca^2+^ as an intermediate configuration with some concentration of data near Δ = 0. Some of these differences may be due to the distortion parameter that we use not representing different distributions of bond lengths within a polyhedron, as this does affect somewhat the behaviour of mean bond length as a function of distortion (Urusov, 2003 ▸, 2014 ▸). However, this issue does not affect the mean bond length at zero distortion. Thus it is apparent from the large amount of scatter in Fig. 20 ▸ (Figs. S5 and S6) that much of the variation in mean bond length shown in Figs. 14 ▸ and 15 ▸ (Figs. S3 and S4) is not due to distortion, and that one or more other factors must also affect mean bond length.

### 10.2. Atomic displacement   

We selected a sample of 56 coordination polyhedra from the parent distribution of ^[6]^Na^+^ (920 coordination polyhedra) to examine other possible factors that may correlate with mean bond length; these samples were selected so that they were representative of all values of distortion.

**Figure 21:**
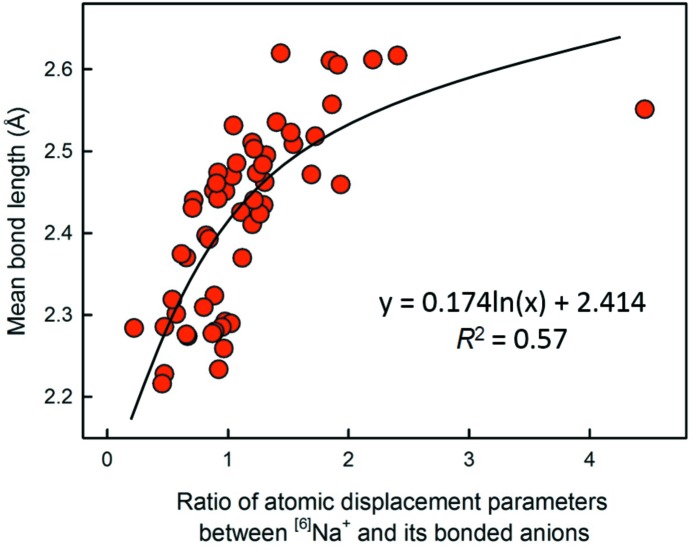
The correlation of atomic displacement with mean bond length. Values are for the ratio of the atomic displacement parameters between ^[6]^Na^+^ and its bonded anions (*n* = 56).

When examining the data for outliers that were possible erroneous data, we noticed very large relative variations in anisotropic displacement or equivalent isotropic displacement parameters in the atoms of the parent structures. Cursory examination indicated that the magnitudes of these mean-bond-length outliers often correlated with the atomic displacement of the constituent cations and/or anions, suggesting that the central cation responds to an overly large coordination by increasing its dynamic (or static) displacement, while anions respond to an overly small cation-coordination environment by increasing their own displacement. Shannon (1993 ▸) and Shannon & Rossman (1992 ▸) have commented on the effect of the former when considering the additivity of fictive dielectric constants of ions in crystals, denoting this behaviour as ‘rattling’. Inspection of many structures eventually showed that the ratio *U*_eq(Na)_/*U*_eq(bonded anions)_ was most highly correlated with variation in mean bond length. This is shown in Fig. 21 ▸; the observed correlation is logarithmic, with *R* ^2^ = 0.57. The fit of the correlation is of the same magnitude as that observed for distortion for the same sample of 56 coordination polyhedra (*R* ^2^ = 0.52). We note that Fig. 21 ▸ contains an apparent outlier with an atomic displacement ratio of 4.46, and that this single data point has considerable effect on whether the shape of the regression curve is logarithmic or linear. Removing this data point, a linear regression gives *R* ^2^ = 0.62, while the logarithmic fit becomes *R* ^2^ = 0.58. However, we found no justification for the removal of this data point upon examination of the structure.

### 10.3. The relation between mean bond length and atomic displacement   

The correlation of mean bond length with the atomic displacement parameter has not been discussed extensively in previous work on variation in mean bond lengths. In well ordered crystal structures, there is generally a positive correlation between atomic displacement parameters and coordination number. As shown above, for ^[6]^Na^+^ there is a positive correlation between atomic displacement parameters and mean bond length. Taken together, these two observations suggest that the atomic displacements increase as the strength of the constituent chemical bonds decreases, and that such variation in atomic displacement accompanies variation in bond lengths that occur due to other factors such as bond-length distortion.

For a specific cation with a specific coordination number, one expects the following sequence: (1) over a particular range of distances, the atomic displacement increases with increasing distance; (2) with further increase in distance, continuous displacement changes to discontinuous displacement, *i.e.* hopping of the central cation in an overly large coordination polyhedron; (3) static displacement of the cation away from the centre of the coordination polyhedron; (4) collapse of the anions forming the coordination polyhedron (perhaps *via* a ferroelastic phase transition), reducing the coordination number and changing the symmetry of the structure. When considering the factors affecting bond length, we need to recognize the relation between the type of displacement behaviour of the central cation and mean bond-length. In stage (1), there is an increase in mean bond length due to local differences in structures (together with a monotonic change in vibrational displacement). In stage (2), the question arises as to whether the observed mean distances are comparable with those of stage (1) as they are accompanied by large displacement parameters characteristic of atom hopping. In stage (3) the observed distances are not affected by atom hopping, but a change in coordination number of the central cation may be observed. When examining variation in mean bond length for a specific ion in a particular coordination, it is important to limit the data to structures at stage (1), as once hopping occurs, an additional component is added to the measured mean bond length that is not present at smaller distances.

## 11. Summary   

(1) We have examined the bond-length distributions for 55 configurations of alkali-metal ions and 29 configurations of alkaline-earth-metal ions, for 4859 coordination polyhedra and 38 594 bond distances (alkali metals) and for 3038 coordination polyhedra and 24 487 bond distances (alkaline-earth metals).

(2) Bond lengths generally show a positively skewed Gaussian distribution that originates from the variation in Born repulsion and Coulomb attraction as a function of interatomic distance.

(3) The skewness and kurtosis of these distributions generally decrease with increasing coordination number of the central cation, a result of decreasing Born repulsion with increasing coordination number.

(4) We confirm the following minimum coordination numbers: ^[3]^Li^+^, ^[3]^Na^+^, ^[4]^K^+^, ^[4]^Rb^+^, ^[6]^Cs^+^, ^[3]^Be^2+^, ^[4]^Mg^2+^, ^[6]^Ca^2+^, ^[6]^Sr^2+^ and ^[6]^Ba^2+^, but note that some reported examples are the result of extensive dynamic and/or positional short-range disorder and are not ordered arrangements.

(5) Some distributions of bond lengths are distinctly multi-modal (primarily bimodal), but for the alkali-metal and alkaline-earth-metal ions, this is often due to the occurrence of large numbers of structure refinements of a particular structure-type in which a particular cation is always present, *e.g.* for ^[8]^Ca^2+^, in which many refinements of garnet and vesuvianite structures lead to an over-representation of specific bond lengths.

(6) For alkali-metal and alkaline-earth-metal ions, there is a positive correlation between incident bond-valence sum at the central cation and coordination number, the values varying from 0.84 v.u. for ^[5]^K^+^ to 1.06 v.u. for ^[8]^Li^+^, and from 1.76 v.u. for ^[7]^Ba^2+^ to 2.10 v.u. for ^[12]^Sr^2+^.

(7) Unusually small or large coordination numbers are commonly associated with anomalous values of atomic displacement of the constituent cations and/or anions.

(8) For a sample of ^[6]^Na, the ratio *U*_eq(Na)_/*U*_eq(bonded anions)_ is partially correlated with 〈^[6]^Na—O〉 mean bond length (*R* ^2^ = 0.57), suggesting that the vibrational/displacement characteristics of the constituent ions are affected by mean bond length for a fixed coordination number.

(9) Mean bond lengths show a weak correlation with bond-length distortion from the mean value, but clearly also correlate with one or more other factors, *e.g.* atomic displacement. In particular, some coordination numbers show the widest variation in mean bond length for zero distortion, *e.g.* Li^+^ in [4]- and [6]-coordination, Na^+^ in [4]- and [6]-coordination and K^+^ in [6]-coordination, and for [4]-coordinated Be^2+^, [4]- and [6]-coordinated Mg^2+^, [6]- and [12]-coordinated Ca^2+^, [6]-coordinated Sr^2+^ and [12]-coordinated Ba^2+^.

(10) Bond-valence parameters for the four ions observed in coordinations higher than [12], K^+^, Rb^+^, Cs^+^ and Ba^2+^ (Gagné & Hawthorne, 2015 ▸) were calculated for a maximum coordination number of [12]. Both sets of parameters give exactly the same result for anion bond-valence sums. However, the bond-valence parameters calculated for a maximum coordination number of [12] show much poorer correlation with mean observed bond length and no correlation at all with ionization energy of the central cation, in contrast to the bond-valence parameters of Gagné & Hawthorne (2015 ▸).

## Supplementary Material

Supporting figures. DOI: 10.1107/S2052520616008507/yb5012sup1.pdf

Raw data file for Ba2+. DOI: 10.1107/S2052520616008507/yb5012sup2.txt

Raw data file for Be2+. DOI: 10.1107/S2052520616008507/yb5012sup3.txt

Raw data file for Ca2+. DOI: 10.1107/S2052520616008507/yb5012sup4.txt

Raw data file for Cs+. DOI: 10.1107/S2052520616008507/yb5012sup5.txt

Raw data file for K+. DOI: 10.1107/S2052520616008507/yb5012sup6.txt

Raw data file for Li+. DOI: 10.1107/S2052520616008507/yb5012sup7.txt

Raw data file for Mg2+. DOI: 10.1107/S2052520616008507/yb5012sup8.txt

Raw data file for Na+. DOI: 10.1107/S2052520616008507/yb5012sup9.txt

Raw data file for Rb+. DOI: 10.1107/S2052520616008507/yb5012sup10.txt

Raw data file for Sr2+. DOI: 10.1107/S2052520616008507/yb5012sup11.txt

## Figures and Tables

**Table 1:** Bond-length statistics for the five common alkali-metal ions

Ion	Coordination number	Number of bonds	Number of coordination polyhedra	Mean bond length (Å)	Standard deviation (Å)	Range (Å)	Maximum bond length (Å)	Minimum bond length (Å)	Skewness	Kurtosis
Li^+^	3	12	4	1.913	0.040	0.113	1.984	1.871	–	–
	4	1676	419	1.972	0.075	0.754	2.444	1.690	1.563	6.273
	5	455	91	2.108	0.161	0.858	2.673	1.815	1.265	1.362
	6	1038	173	2.178	0.137	0.866	2.692	1.826	1.293	1.996
	7	14	2	2.331	0.244	0.728	2.658	1.930	–	–
	8	8	1	2.513	0.000	0.000	2.513	2.513	–	–
	Total	3203	690							
Na^+^	3	21	7	2.307	0.045	0.153	2.367	2.214	–	–
	4	524	131	2.359	0.076	0.586	2.775	2.189	0.878	2.032
	5	710	142	2.413	0.108	0.808	2.968	2.160	1.455	3.522
	6	5520	920	2.441	0.112	0.926	3.055	2.129	1.317	3.246
	7	1351	193	2.541	0.180	1.002	3.180	2.178	0.723	−0.020
	8	1608	201	2.599	0.192	1.069	3.211	2.142	0.461	−0.197
	9	450	50	2.686	0.224	1.044	3.204	2.160	0.121	−0.879
	10	170	17	2.741	0.240	0.899	3.156	2.257	−0.063	−1.075
	12	252	21	2.795	0.182	0.950	3.272	2.322	0.053	−0.295
	Total	10606	1682							
K^+^	4	96	24	2.708	0.078	0.365	2.892	2.527	–	–
	5	295	59	2.796	0.162	1.032	3.537	2.505	1.404	3.349
	6	714	119	2.828	0.177	1.140	3.587	2.447	1.299	2.268
	7	469	67	2.861	0.179	1.030	3.554	2.524	1.269	1.474
	8	2272	284	2.894	0.172	1.107	3.644	2.537	1.195	1.642
	9	2772	308	2.955	0.214	1.306	3.797	2.491	1.079	0.917
	10	2430	243	3.013	0.246	1.248	3.773	2.525	0.839	0.084
	11	957	87	3.089	0.290	1.181	3.793	2.612	0.550	−0.763
	12	2328	194	3.095	0.264	1.204	3.790	2.586	0.578	−0.535
	13	455	35	3.149	0.298	1.290	3.808	2.518	0.364	−0.834
	14	336	24	3.239	0.296	1.292	3.771	2.479	0.057	−0.929
	15	525	35	3.182	0.265	1.148	3.748	2.600	0.257	−1.164
	Total	13649	1479							
Rb^+^	4	48	12	2.951	0.160	0.706	3.360	2.654	–	–
	5	15	3	2.864	0.078	0.236	2.976	2.740	–	–
	6	84	14	2.989	0.148	0.734	3.488	2.754	–	–
	7	140	20	3.002	0.186	0.937	3.628	2.691	1.283	1.554
	8	480	60	3.033	0.190	1.055	3.711	2.656	1.030	1.236
	9	675	75	3.079	0.204	1.172	3.839	2.667	0.980	1.192
	10	1080	108	3.146	0.244	1.171	3.890	2.719	0.858	0.081
	11	484	44	3.188	0.248	1.211	3.884	2.673	0.725	−0.242
	12	840	70	3.228	0.288	1.181	3.896	2.715	0.545	−0.663
	13	247	19	3.293	0.314	1.148	3.932	2.784	0.376	−1.163
	14	280	20	3.301	0.291	1.151	3.895	2.744	0.332	−0.938
	15	195	13	3.338	0.278	1.065	3.945	2.880	0.485	−0.934
	17	17	1	3.456	0.336	1.009	3.939	2.930	–	–
	18	90	5	3.478	0.239	0.695	3.771	3.076	–	–
	Total	4675	464							
Cs^+^	6	102	17	3.124	0.139	0.702	3.568	2.866	0.922	1.040
	7	70	10	3.193	0.166	0.858	3.806	2.948	–	–
	8	200	25	3.244	0.189	1.017	3.911	2.894	0.863	0.708
	9	432	48	3.251	0.181	1.052	3.882	2.830	0.656	0.186
	10	930	93	3.304	0.208	1.257	3.986	2.729	0.696	0.174
	11	748	68	3.333	0.226	1.136	4.023	2.887	0.630	−0.106
	12	1080	90	3.377	0.250	1.162	4.072	2.910	0.670	−0.437
	13	520	40	3.426	0.276	1.256	4.130	2.874	0.480	−0.571
	14	714	51	3.444	0.297	1.242	4.169	2.927	0.528	−0.764
	15	705	47	3.503	0.301	1.253	4.157	2.904	0.241	−0.953
	16	224	14	3.550	0.321	1.276	4.199	2.923	0.341	−0.870
	17	68	4	3.530	0.326	1.069	4.060	2.991	–	–
	18	648	36	3.570	0.277	1.243	4.195	2.952	−0.010	−0.807
	20	20	1	3.723	0.419	0.855	4.065	3.210	–	–
	Total	6461	544							

**Table 2:** Bond-length statistics for the five common alkaline-earth-metal ions

Ion	Coordination number	Number of bonds	Number of coordination polyhedra	Mean bond length (Å)	Standard deviation (Å)	Range (Å)	Maximum bond length (Å)	Minimum bond length (Å)	Skewness	Kurtosis
Be^2+^	3	24	8	1.550	0.018	0.081	1.587	1.506	–	–
	4	644	161	1.637	0.040	0.346	1.887	1.541	1.988	8.529
	Total	668	169							
Mg^2+^	4	48	12	1.939	0.020	0.068	1.977	1.909	–	–
	5	120	24	2.044	0.066	0.342	2.249	1.907	0.643	0.271
	6	2556	426	2.089	0.059	0.562	2.497	1.935	1.976	8.608
	8	56	7	2.255	0.122	0.568	2.582	2.014	–	–
	Total	2780	469							
Ca^2+^	6	1266	211	2.371	0.069	0.637	2.847	2.210	1.636	5.908
	7	2009	287	2.448	0.133	1.016	3.140	2.124	1.828	4.607
	8	4152	519	2.498	0.151	1.027	3.176	2.149	1.430	2.739
	9	1035	115	2.559	0.196	0.956	3.197	2.241	1.247	0.939
	10	160	16	2.632	0.215	0.902	3.122	2.220	0.478	−0.400
	11	77	7	2.614	0.177	0.686	2.965	2.279	–	–
	12	156	13	2.668	0.175	0.791	3.117	2.326	0.724	0.379
	Total	8855	1168							
Sr^2+^	6	78	13	2.477	0.050	0.244	2.591	2.347	–	–
	7	266	38	2.638	0.151	0.937	3.306	2.369	1.553	3.693
	8	904	113	2.656	0.163	1.019	3.368	2.349	1.711	3.740
	9	909	101	2.704	0.178	1.088	3.397	2.309	1.545	3.094
	10	500	50	2.769	0.213	1.033	3.399	2.366	0.962	0.456
	11	88	8	2.798	0.198	0.837	3.319	2.482	–	–
	12	636	53	2.825	0.181	0.953	3.358	2.405	0.744	0.613
	Total	3381	376							
Ba^2+^	6	126	21	2.689	0.094	0.400	2.866	2.466	−0.404	−0.665
	7	147	21	2.792	0.135	0.855	3.369	2.514	1.133	2.916
	8	704	88	2.816	0.129	0.846	3.376	2.530	0.962	1.273
	9	1278	142	2.860	0.154	1.074	3.554	2.480	1.000	1.330
	10	1930	193	2.915	0.155	1.014	3.498	2.484	0.783	1.072
	11	902	82	2.944	0.181	1.023	3.612	2.589	0.667	0.074
	12	3624	302	2.965	0.152	0.988	3.624	2.636	1.239	1.951
	13	78	6	3.010	0.206	0.922	3.463	2.541	–	–
	14	14	1	3.080	0.242	0.879	3.553	2.674	–	–
	Total	8813	856							

**Table 3:** Bond-valence parameters for large alkali and alkaline-earth metals calculated with and without a hard cut-off of 12 bonds

Ion	*R*_o_ (Å)	*B* (Å)	RMSD (v.u.)
K^+^	2.047	0.398	0.164
K^+^ (CN ≤ 12)	1.985	0.425	0.157
Rb^+^	1.993	0.478	0.150
Rb^+^ (CN ≤ 12)	1.780	0.577	0.148
Cs^+^	2.305	0.411	0.138
Cs^+^ (CN ≤ 12)	1.966	0.561	0.138
Ba^2+^	2.223	0.406	0.217
Ba^2+^ (CN ≤ 12)	2.208	0.417	0.215

**Table 4:** Mean bond-length statistics for the five common alkali-metal ions

Ion	Coordination number	Number of coordination polyhedra	Grand mean bond-length (Å)	Standard deviation (Å)	Mean bond-length range (Å)	Maximum mean bond-length (Å)	Minimum mean bond-length (Å)	Skewness	Kurtosis
Li^+^	3	4	1.913	0.032	0.070	1.958	1.888	–	–
	4	419	1.972	0.039	0.287	2.162	1.875	0.803	2.171
	5	91	2.108	0.050	0.214	2.226	2.012	–	–
	6	173	2.178	0.052	0.311	2.360	2.048	0.886	1.192
	7	2	2.331	0.008	0.012	2.337	2.326	–	–
	8	1	2.513	–	–	2.513	2.513	–	–
Na^+^	3	7	2.307	0.045	0.112	2.360	2.248	–	–
	4	131	2.359	0.049	0.285	2.537	2.252	0.784	0.937
	5	142	2.413	0.049	0.269	2.561	2.292	0.209	−0.034
	6	920	2.441	0.056	0.466	2.682	2.216	0.295	2.367
	7	193	2.541	0.056	0.313	2.718	2.404	0.423	0.021
	8	201	2.599	0.071	0.458	2.880	2.422	0.574	1.160
	9	50	2.686	0.065	0.382	2.936	2.553	–	–
	10	17	2.741	0.054	0.183	2.830	2.647	–	–
	12	21	2.795	0.074	0.254	2.940	2.686	–	–
K^+^	4	24	2.708	0.056	0.234	2.852	2.618	–	–
	5	59	2.796	0.078	0.438	3.084	2.646	–	–
	6	119	2.828	0.103	0.652	3.099	2.447	0.022	1.007
	7	67	2.861	0.064	0.295	3.009	2.715	–	–
	8	284	2.894	0.053	0.299	3.081	2.781	0.619	0.462
	9	308	2.955	0.061	0.503	3.174	2.671	−0.003	2.167
	10	243	3.013	0.065	0.382	3.209	2.827	0.384	0.493
	11	87	3.089	0.066	0.306	3.242	2.936	–	–
	12	194	3.095	0.092	0.543	3.337	2.794	−0.708	1.232
	13	35	3.149	0.094	0.327	3.293	2.966	–	–
	14	24	3.239	0.068	0.288	3.374	3.085	–	–
	15	35	3.182	0.064	0.246	3.332	3.086	–	–
Rb^+^	4	12	2.776	0.634	2.252	3.018	0.765	–	–
	5	3	2.864	0.040	0.078	2.899	2.821	–	–
	6	14	2.989	0.084	0.286	3.122	2.836	–	–
	7	20	3.002	0.083	0.371	3.213	2.842	–	–
	8	60	3.033	0.058	0.260	3.182	2.922	–	–
	9	75	3.079	0.064	0.321	3.287	2.966	–	–
	10	108	3.142	0.064	0.302	3.279	2.977	−0.262	−0.405
	11	44	3.188	0.054	0.226	3.296	3.070	–	–
	12	70	3.228	0.084	0.373	3.410	3.037	–	–
	13	19	3.293	0.042	0.138	3.350	3.212	–	–
	14	20	3.301	0.061	0.235	3.400	3.164	–	–
	15	13	3.338	0.110	0.337	3.545	3.208	–	–
	17	1	3.456	–	0.000	3.456	3.456	–	–
	18	5	3.478	0.078	0.217	3.582	3.364	–	–
Cs^+^	6	17	3.124	0.032	0.113	3.175	3.062	–	–
	7	10	3.193	0.061	0.206	3.312	3.106	–	–
	8	25	3.244	0.064	0.216	3.359	3.143	–	–
	9	48	3.251	0.054	0.232	3.366	3.134	–	–
	10	93	3.304	0.056	0.348	3.455	3.107	–	–
	11	68	3.333	0.054	0.261	3.479	3.218	–	–
	12	90	3.377	0.072	0.335	3.542	3.207	–	–
	13	40	3.426	0.063	0.267	3.552	3.285	–	–
	14	51	3.444	0.064	0.229	3.539	3.310	–	–
	15	47	3.503	0.066	0.289	3.661	3.372	–	–
	16	14	3.550	0.068	0.230	3.672	3.442	–	–
	17	4	3.530	0.029	0.062	3.549	3.487	–	–
	18	36	3.570	0.062	0.229	3.715	3.487	–	–
	20	1	3.723	–	–	3.723	3.723	–	–

**Table 5:** Mean bond-length statistics for the five common alkaline-earth-metal ions

Ion	Coordination number	Number of coordination polyhedra	Grand mean bond length (Å)	Standard deviation (Å)	Mean bond-length range (Å)	Maximum mean bond length (Å)	Minimum mean bond length (Å)	Skewness	Kurtosis
Be^2+^	3	8	1.550	0.012	0.031	1.566	1.535	–	–
	4	161	1.637	0.017	0.114	1.719	1.605	1.744	6.011
Mg^2+^	4	12	1.939	0.017	0.054	1.966	1.912	–	–
	5	24	1.966	0.091	0.309	2.087	1.777	–	–
	6	426	2.089	0.024	0.185	2.223	2.038	1.179	3.051
	8	7	2.255	0.035	0.080	2.284	2.203	–	–
Ca^2+^	6	211	2.371	0.034	0.217	2.471	2.254	−0.261	0.236
	7	287	2.447	0.038	0.225	2.591	2.366	0.735	0.761
	8	519	2.498	0.048	0.259	2.663	2.404	0.216	−0.238
	9	115	2.559	0.039	0.215	2.694	2.479	0.761	0.927
	10	16	2.632	0.048	0.137	2.686	2.549	–	–
	11	7	2.614	0.060	0.139	2.690	2.551	–	–
	12	13	2.668	0.073	0.228	2.777	2.549	–	–
Sr^2+^	6	13	2.477	0.034	0.118	2.542	2.424	–	–
	7	38	2.639	0.054	0.188	2.738	2.549	–	–
	8	113	2.658	0.061	0.310	2.871	2.561	1.298	1.633
	9	101	2.703	0.051	0.266	2.871	2.604	1.119	1.523
	10	50	2.769	0.070	0.436	3.053	2.617	–	–
	11	8	2.798	0.027	0.075	2.830	2.755	–	–
	12	53	2.825	0.055	0.213	2.930	2.716	–	–
Ba^2+^	6	21	2.689	0.054	0.197	2.794	2.597	–	–
	7	21	2.792	0.033	0.121	2.855	2.733	–	–
	8	88	2.816	0.049	0.254	2.952	2.698	–	–
	9	142	2.860	0.055	0.307	3.036	2.729	0.111	0.302
	10	193	2.915	0.053	0.295	3.091	2.796	0.282	−0.022
	11	82	2.944	0.039	0.187	3.035	2.849	–	–
	12	302	2.965	0.058	0.289	3.133	2.845	0.467	−0.299
	13	6	3.010	0.051	0.137	3.070	2.934	–	–
	14	1	3.080	–	–	3.080	3.080	–	–

## Footnotes

1 A kernel-density estimate shows the same distribution as a histogram. A histogram assigns an exact value to a piece of data, whereas a kernel-density estimate associates a variance with each piece of data and produces a much simpler plot. We use kernel-density estimates in this paper where we want to show several distributions on one figure, as several overlapping histograms are difficult to compare, whereas overlapping kernel-density estimates are visually more simple and much more easily compared.

2 There are notable exceptions to this criterion. For example, in the structure of RbLiO (Sabrowsky & Vogt, 1987 ▸), Rb^+^ is surrounded by six O^2−^ ions with distances 2.897, 2.927 ×2, 3.247 ×2 and 3.488 Å, but the shortest Rb—Li distance is 2.916 Å, shorter than five of the six shortest Rb^+^—O^2−^ distances. Another example occurs in RbNaO (Sabrowsky, Thimm & Vogt-Mertens, 1985 ▸); Rb^+^ is surrounded by six O^2−^ ions with distances 2.905, 3.066 ×4 and 3.589 Å, but the shortest Rb—Na distance is 3.089 Å ×4, very similar to the four intermediate length Rb^+^—O^2−^ distances. Similar behaviour is shown by KNaO (Sabrowsky & Schroeer, 1982 ▸). However, this behaviour appears to be confined to mixed alkali-metal oxides.
