# Supplementary material for: Bond-length distributions for ions bonded to oxygen: alkali and alkaline-earth metals
Source: Acta Crystallogr B Struct Sci Cryst Eng Mater. 2016 Aug 1;72(Pt 4):602–25. doi: 10.1107/S2052520616008507 (PMC4971548; doi:10.1107/S2052520616008507)
Supplement: Supplementary file 1 [file b-72-00602-sup1.pdf]

STRUCTURAL SCIENCE
CRYSTAL ENGINEERING
MATERIALS

Volume 72 (2016)

Supporting information for article:

Bond-length distributions for ions bonded to oxygen: Alkali and alkaline-earth metals

Olivier Charles Gagné and Frank Christopher Hawthorne

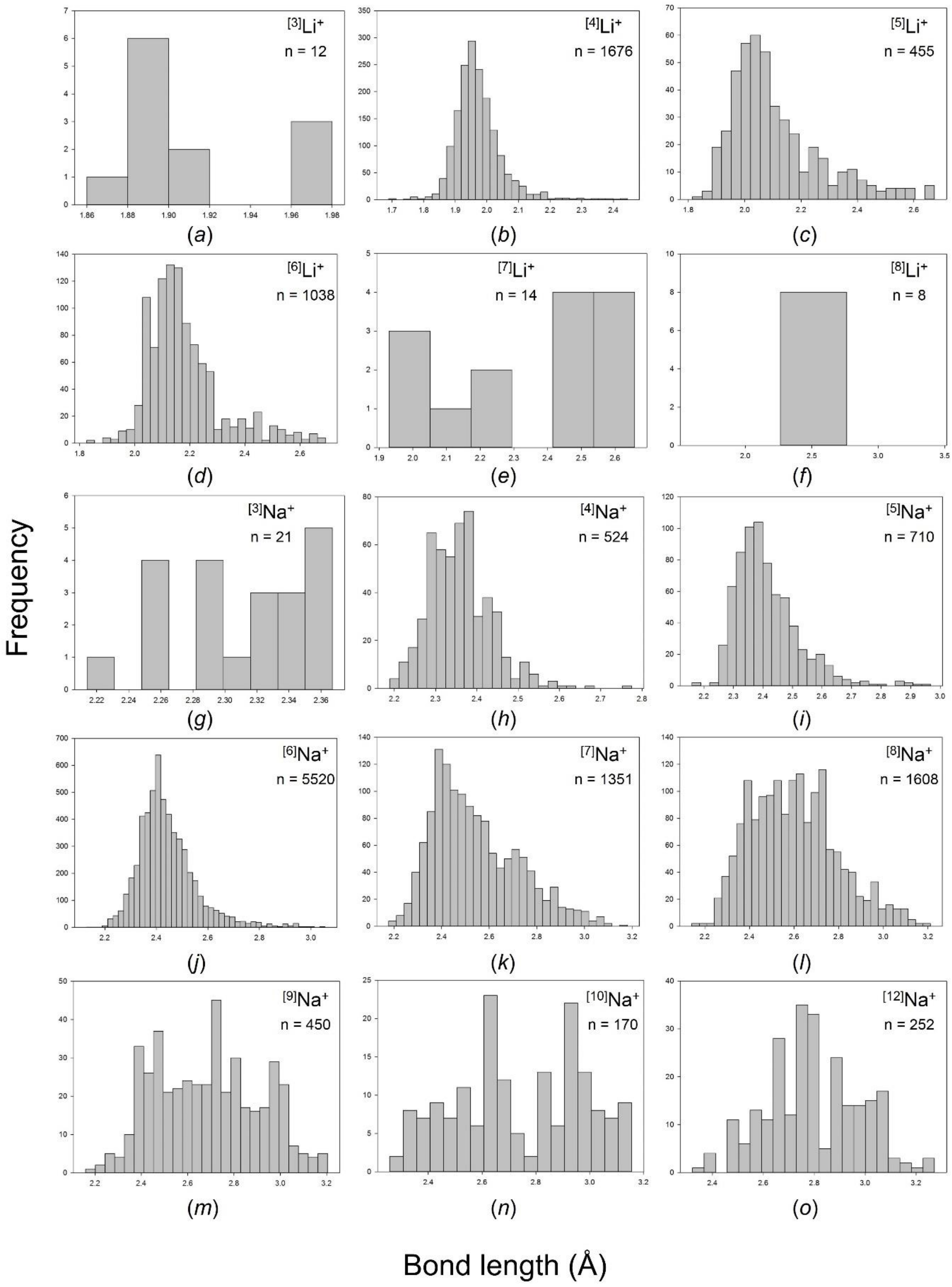
[3]Li+
n = 12
(a)
[4]Li+
n = 1676
(b)
[5]Li+
n = 455
(c)
[6]Li+
n = 1038
(d)
[7]Li+
n = 14
(e)
[8]Li+
n = 8
(f)
[3]Na+
n = 21
(g)
[4]Na+
n = 524
(h)
[5]Na+
n = 710
(i)
[6]Na+
n = 5520
(j)
[7]Na+
n = 1351
(k)
[8]Na+
n = 1608
(l)
[9]Na+
n = 450
(m)
[10]Na+
n = 170
(n)
[12]Na+
n = 252
(o)
Frequency
Bond length (Å)

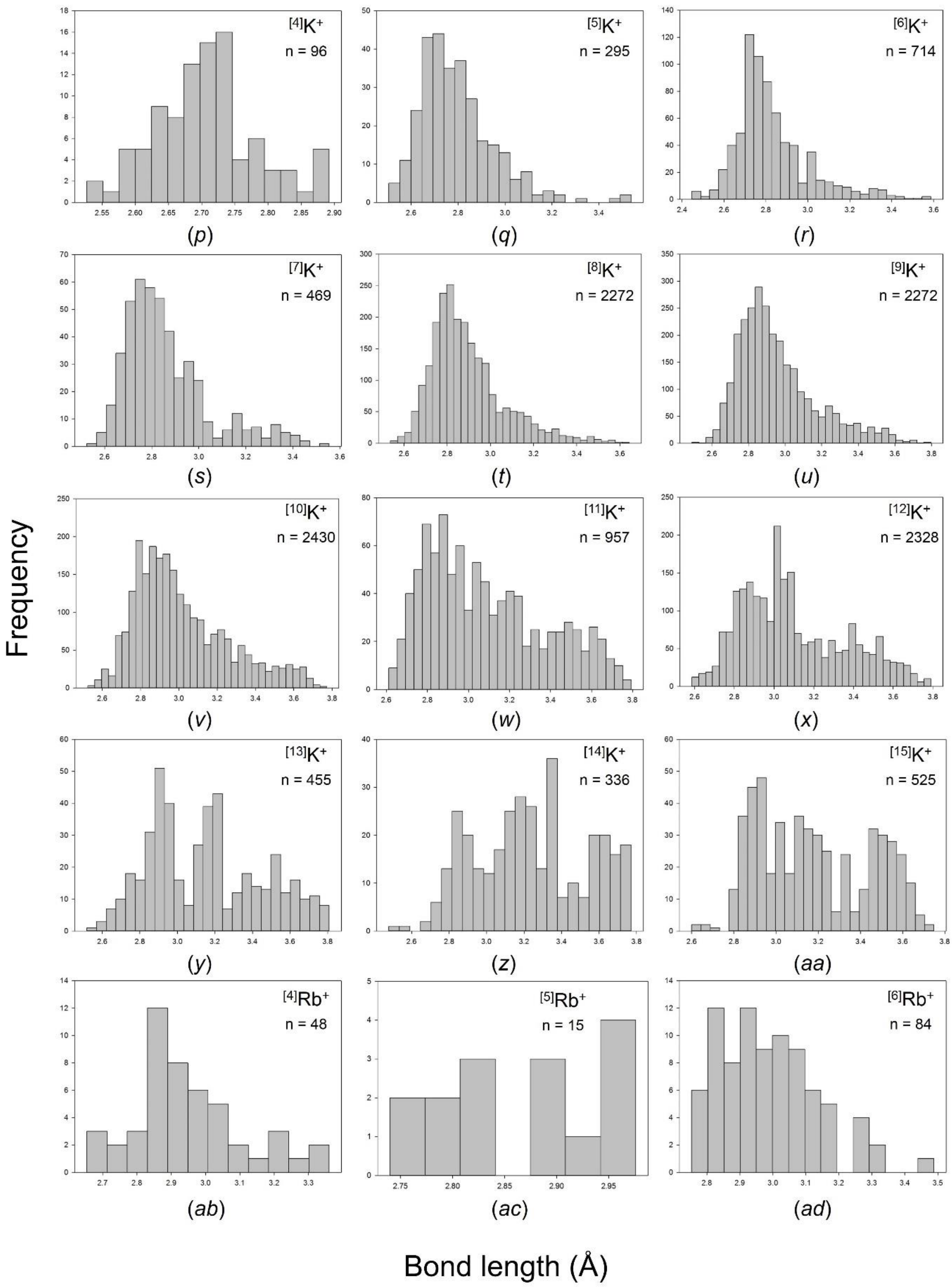

Frequency
[4]K+
n = 96
(p)
[5]K+
n = 295
(q)
[6]K+
n = 714
(r)
[7]K+
n = 469
(s)
[8]K+
n = 2272
(t)
[9]K+
n = 2272
(u)
[10]K+
n = 2430
(v)
[11]K+
n = 957
(w)
[12]K+
n = 2328
(x)
[13]K+
n = 455
(y)
[14]K+
n = 336
(z)
[15]K+
n = 525
(aa)
[4]Rb+
n = 48
(ab)
[5]Rb+
n = 15
(ac)
[6]Rb+
n = 84
(ad)
Bond length (Å)

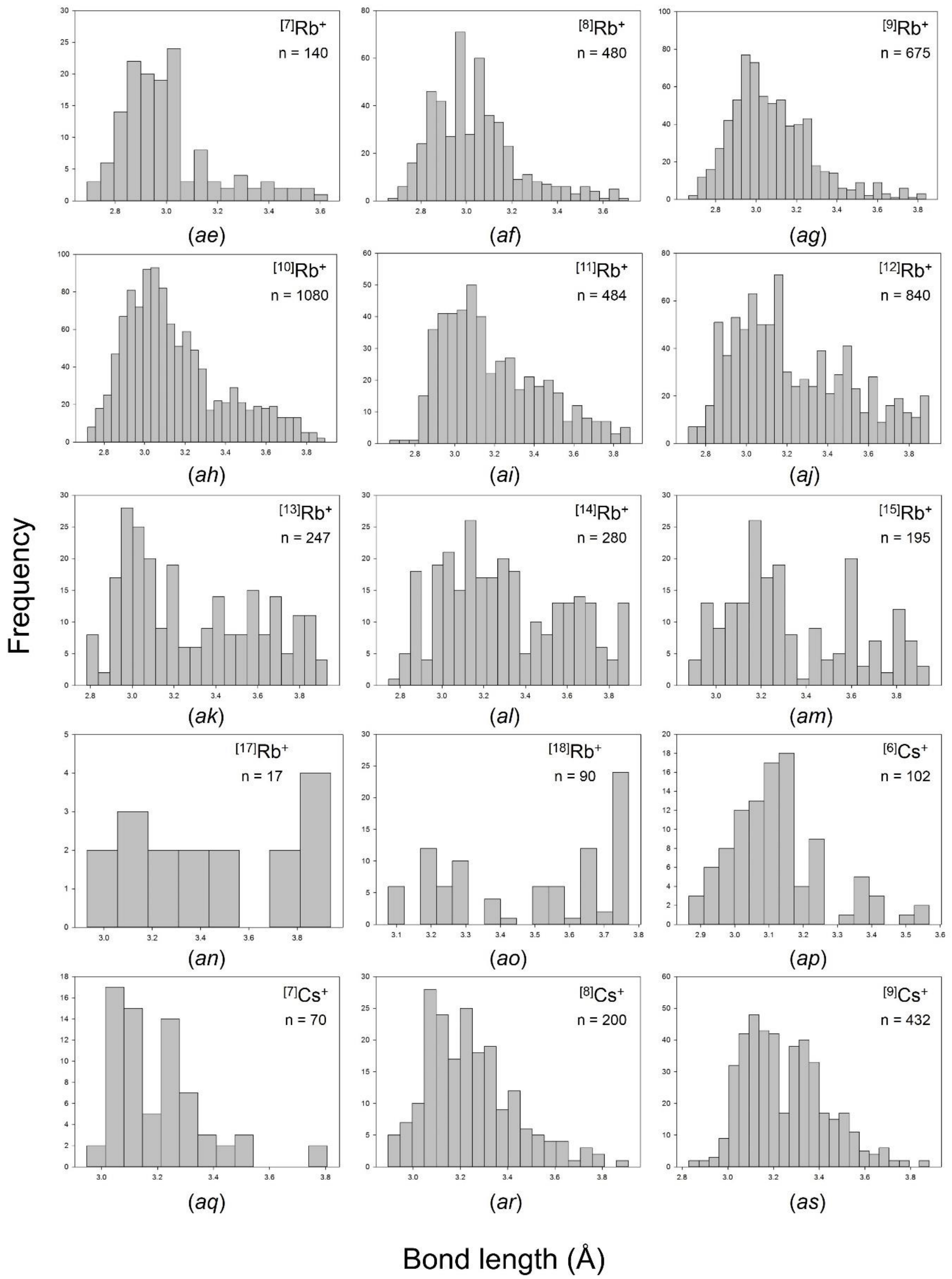
[7]Rb+
n = 140
(ae)
[8]Rb+
n = 480
(af)
[9]Rb+
n = 675
(ag)
[10]Rb+
n = 1080
(ah)
[11]Rb+
n = 484
(ai)
[12]Rb+
n = 840
(aj)
[13]Rb+
n = 247
(ak)
[14]Rb+
n = 280
(al)
[15]Rb+
n = 195
(am)
[17]Rb+
n = 17
(an)
[18]Rb+
n = 90
(ao)
[6]Cs+
n = 102
(ap)
[7]Cs+
n = 70
(aq)
[8]Cs+
n = 200
(ar)
[9]Cs+
n = 432
(as)
Frequency
Bond length (Å)

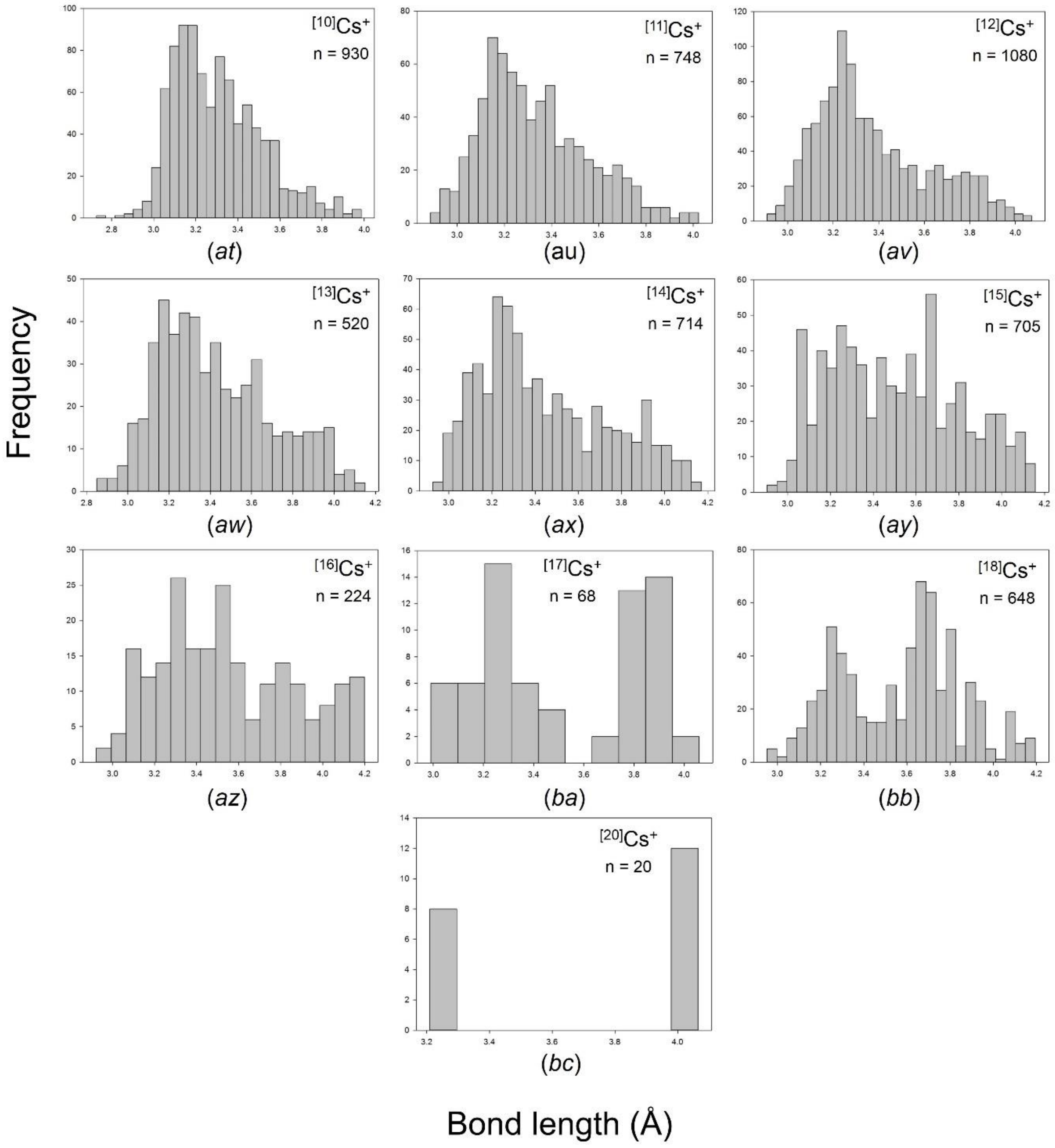

Figure S1 Bond-length distributions for all configurations of the alkali-metal ions bonded to O^{2-}: (*a*) $^{[3]}Li^+$, (*b*) $^{[4]}Li^+$, (*c*) $^{[5]}Li^+$, (*d*) $^{[6]}Li^+$, (*e*) $^{[7]}Li^+$, (*f*) $^{[8]}Li^+$, (*g*) $^{[3]}Na^+$, (*h*) $^{[4]}Na^+$, (*i*) $^{[5]}Na^+$, (*j*) $^{[6]}Na^+$, (*k*) $^{[7]}Na^+$, (*l*) $^{[8]}Na^+$, (*m*) $^{[9]}Na^+$, (*n*) $^{[10]}Na^+$, (*o*) $^{[12]}Na^+$, (*p*) $^{[4]}K^+$, (*q*) $^{[5]}K^+$, (*r*) $^{[6]}K^+$, (*s*) $^{[7]}K^+$, (*t*) $^{[8]}K^+$, (*u*) $^{[9]}K^+$, (*v*) $^{[10]}K^+$, (*w*) $^{[11]}K^+$, (*x*) $^{[12]}K^+$, (*y*) $^{[13]}K^+$, (*z*) $^{[14]}K^+$, (*aa*) $^{[15]}K^+$, (*ab*) $^{[4]}Rb^+$, (*ac*) $^{[5]}Rb^+$, (*ad*) $^{[6]}Rb^+$, (*ae*) $^{[7]}Rb^+$, (*af*) $^{[8]}Rb^+$, (*ag*) $^{[9]}Rb^+$, (*ah*) $^{[10]}Rb^+$, (*ai*) $^{[11]}Rb^+$, (*aj*) $^{[12]}Rb^+$, (*ak*) $^{[13]}Rb^+$, (*al*) $^{[14]}Rb^+$, (*am*) $^{[15]}Rb^+$, (*an*) $^{[17]}Rb^+$, (*ao*) $^{[18]}Rb^+$, (*ap*) $^{[6]}Cs^+$, (*aq*) $^{[7]}Cs^+$, (*ar*) $^{[8]}Cs^+$, (*as*) $^{[9]}Cs^+$, (*at*) $^{[10]}Cs^+$, (*au*) $^{[11]}Cs^+$, (*av*) $^{[12]}Cs^+$, (*aw*) $^{[13]}Cs^+$, (*ax*) $^{[14]}Cs^+$, (*ay*) $^{[15]}Cs^+$, (*az*) $^{[16]}Cs^+$, (*ba*) $^{[17]}Cs^+$, (*bb*) $^{[18]}Cs^+$, (*bc*) $^{[20]}Cs^+$.

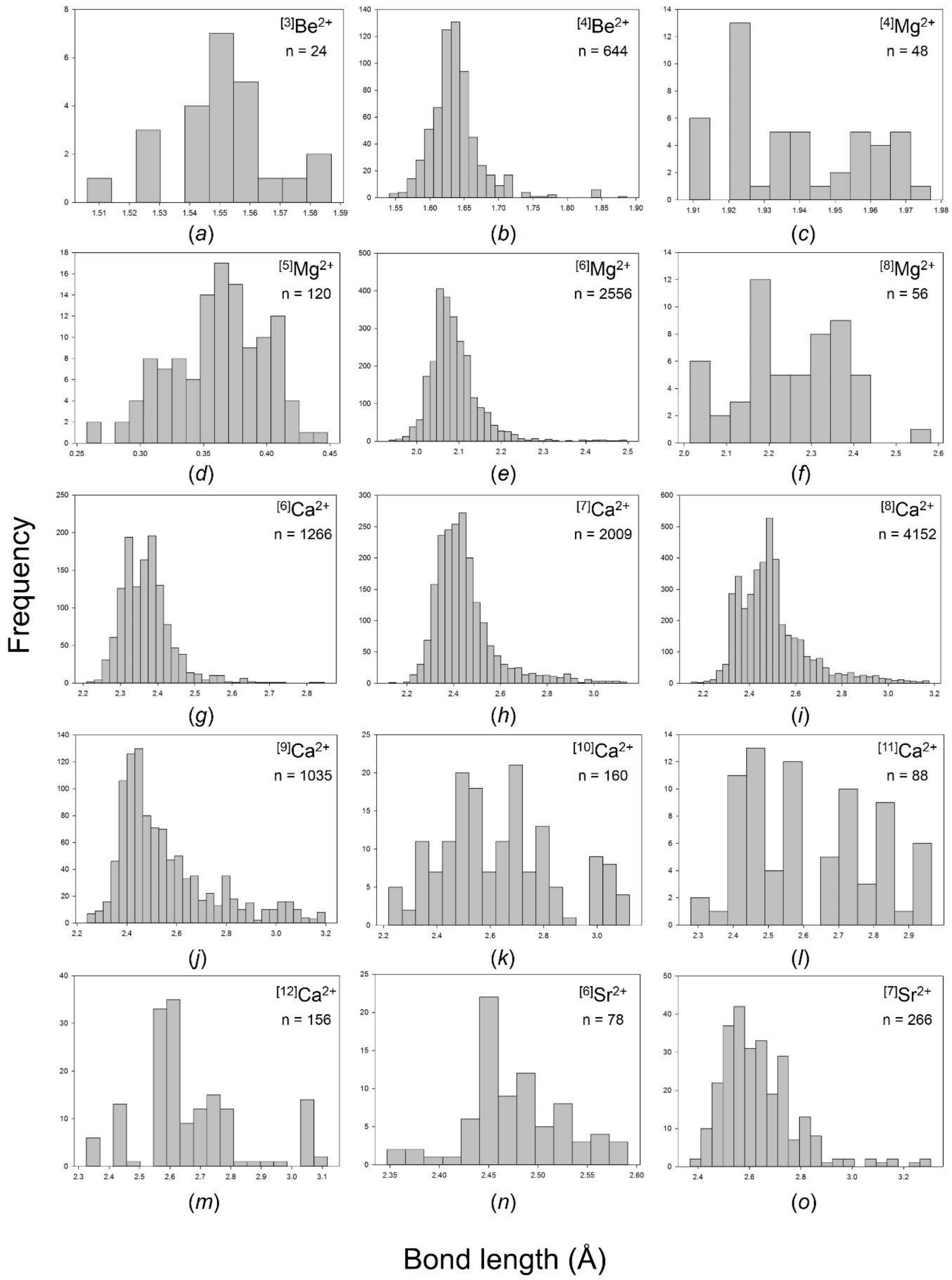
[3]Be2+
n = 24
(a)
[4]Be2+
n = 644
(b)
[4]Mg2+
n = 48
(c)
[5]Mg2+
n = 120
(d)
[6]Mg2+
n = 2556
(e)
[8]Mg2+
n = 56
(f)
[6]Ca2+
n = 1266
(g)
[7]Ca2+
n = 2009
(h)
[8]Ca2+
n = 4152
(i)
[9]Ca2+
n = 1035
(j)
[10]Ca2+
n = 160
(k)
[11]Ca2+
n = 88
(l)
[12]Ca2+
n = 156
(m)
[6]Sr2+
n = 78
(n)
[7]Sr2+
n = 266
(o)
Frequency
Bond length (Å)

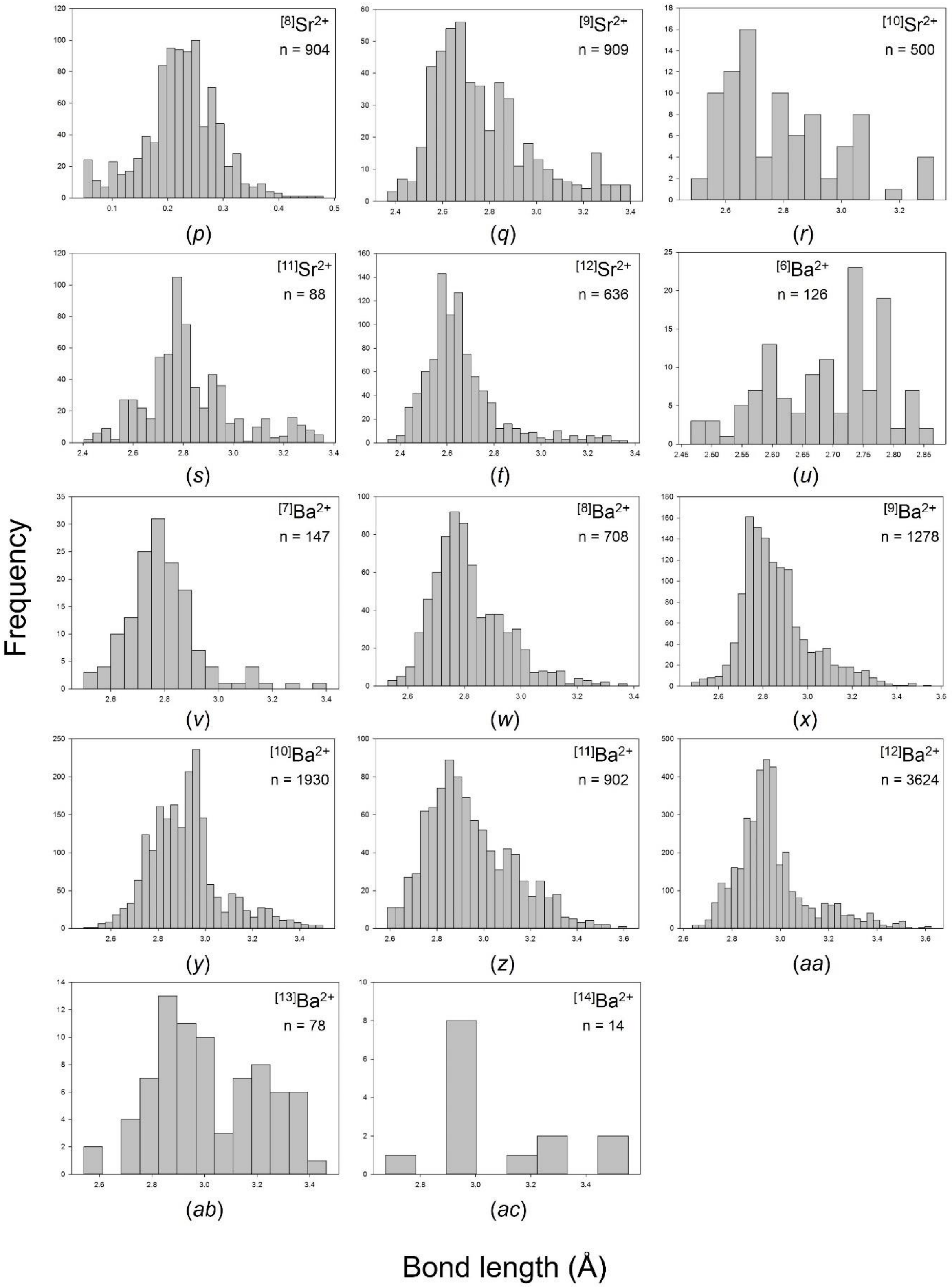

Figure S2 Bond-length distributions for all configurations of the alkaline-earth-metal ions bonded to O^{2-}:(*a*) [3]Be^{2+}, (*b*) [4]Be^{2+}, (*c*) [4]Mg^{2+}, (*d*) [5]Mg^{2+}, (*e*) [6]Mg^{2+}, (*f*) [8]Mg^{2+}, (*g*) [6]Ca^{2+}, (*h*) [7]Ca^{2+}, (*i*) [8]Ca^{2+}, (*j*) [9]Ca^{2+}, (*k*) [10]Ca^{2+}, (*l*) [11]Ca^{2+}, (*m*) [12]Ca^{2+}, (*n*) [6]Sr^{2+}, (*o*) [7]Sr^{2+}, (*p*) [8]Sr^{2+}, (*q*) [9]Sr^{2+}, (*r*)

$^{[10]}Sr^{2+}$, (*s*) $^{[11]}Sr^{2+}$, (*t*) $^{[12]}Sr^{2+}$, (*u*) $^{[6]}Ba^{2+}$, (*v*) $^{[7]}Ba^{2+}$, (*w*) $^{[8]}Ba^{2+}$, (*x*) $^{[9]}Ba^{2+}$, (*y*) $^{[10]}Ba^{2+}$, (*z*) $^{[11]}Ba^{2+}$, (*aa*) $^{[12]}Ba^{2+}$, (*ab*) $^{[13]}Ba^{2+}$, (*ac*) $^{[14]}Ba^{2+}$.

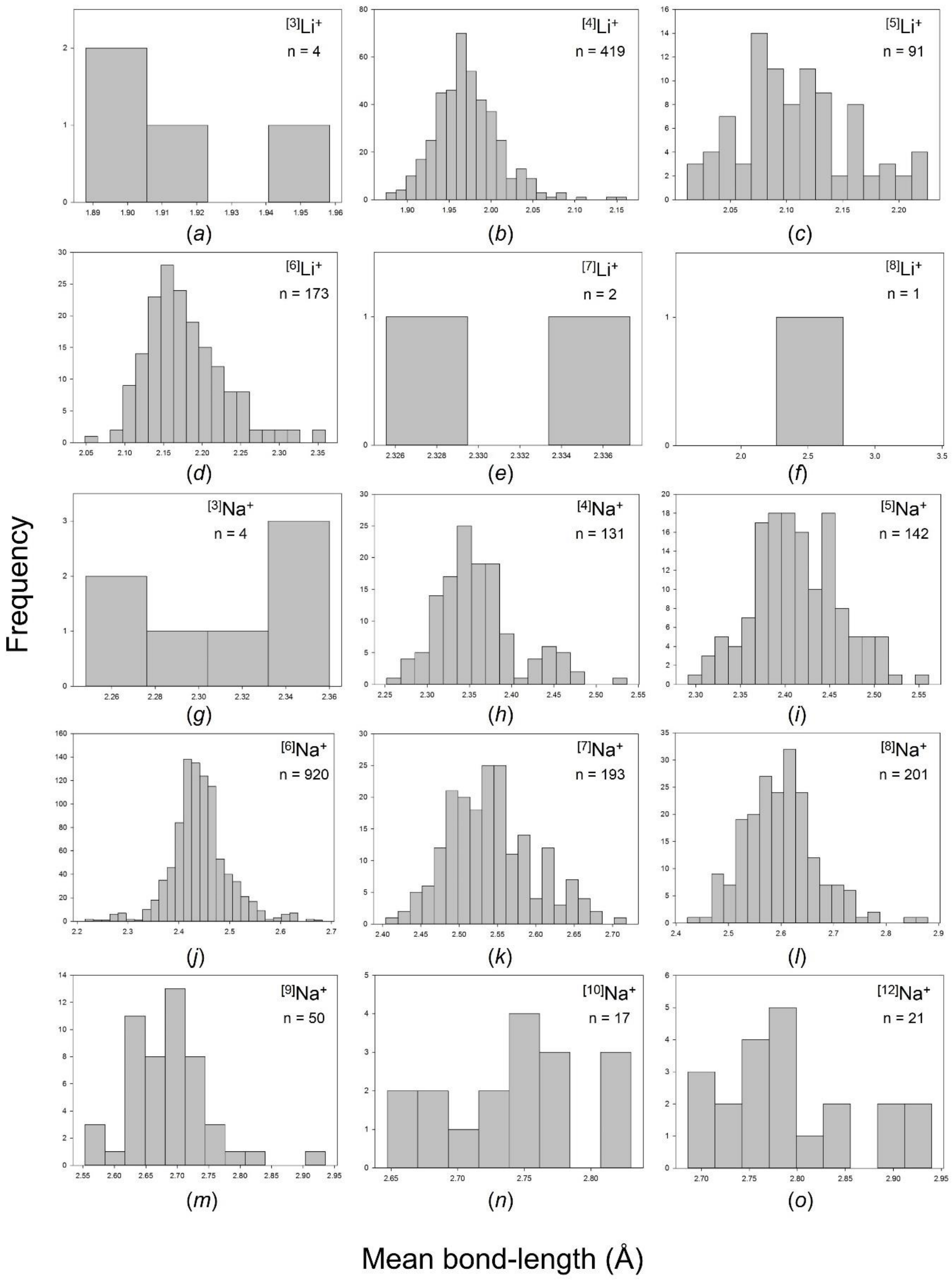
Frequency
Mean bond-length (Å)
[3]Li+
n = 4
(a)
[4]Li+
n = 419
(b)
[5]Li+
n = 91
(c)
[6]Li+
n = 173
(d)
[7]Li+
n = 2
(e)
[8]Li+
n = 1
(f)
[3]Na+
n = 4
(g)
[4]Na+
n = 131
(h)
[5]Na+
n = 142
(i)
[6]Na+
n = 920
(j)
[7]Na+
n = 193
(k)
[8]Na+
n = 201
(l)
[9]Na+
n = 50
(m)
[10]Na+
n = 17
(n)
[12]Na+
n = 21
(o)

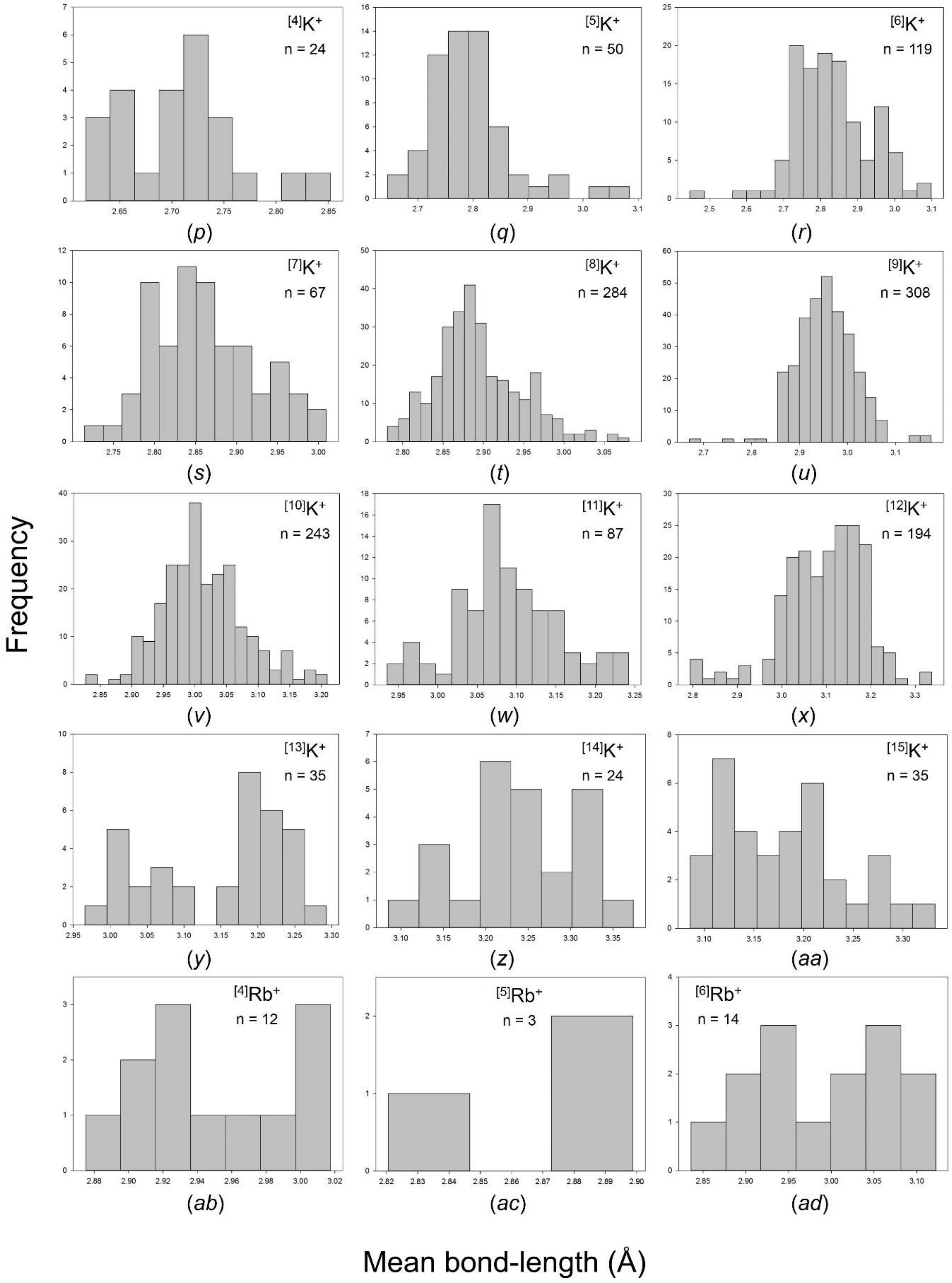
[4]K+
n = 24
(p)
[5]K+
n = 50
(q)
[6]K+
n = 119
(r)
[7]K+
n = 67
(s)
[8]K+
n = 284
(t)
[9]K+
n = 308
(u)
[10]K+
n = 243
(v)
[11]K+
n = 87
(w)
[12]K+
n = 194
(x)
[13]K+
n = 35
(y)
[14]K+
n = 24
(z)
[15]K+
n = 35
(aa)
[4]Rb+
n = 12
(ab)
[5]Rb+
n = 3
(ac)
[6]Rb+
n = 14
(ad)
Frequency
Mean bond-length (Å)

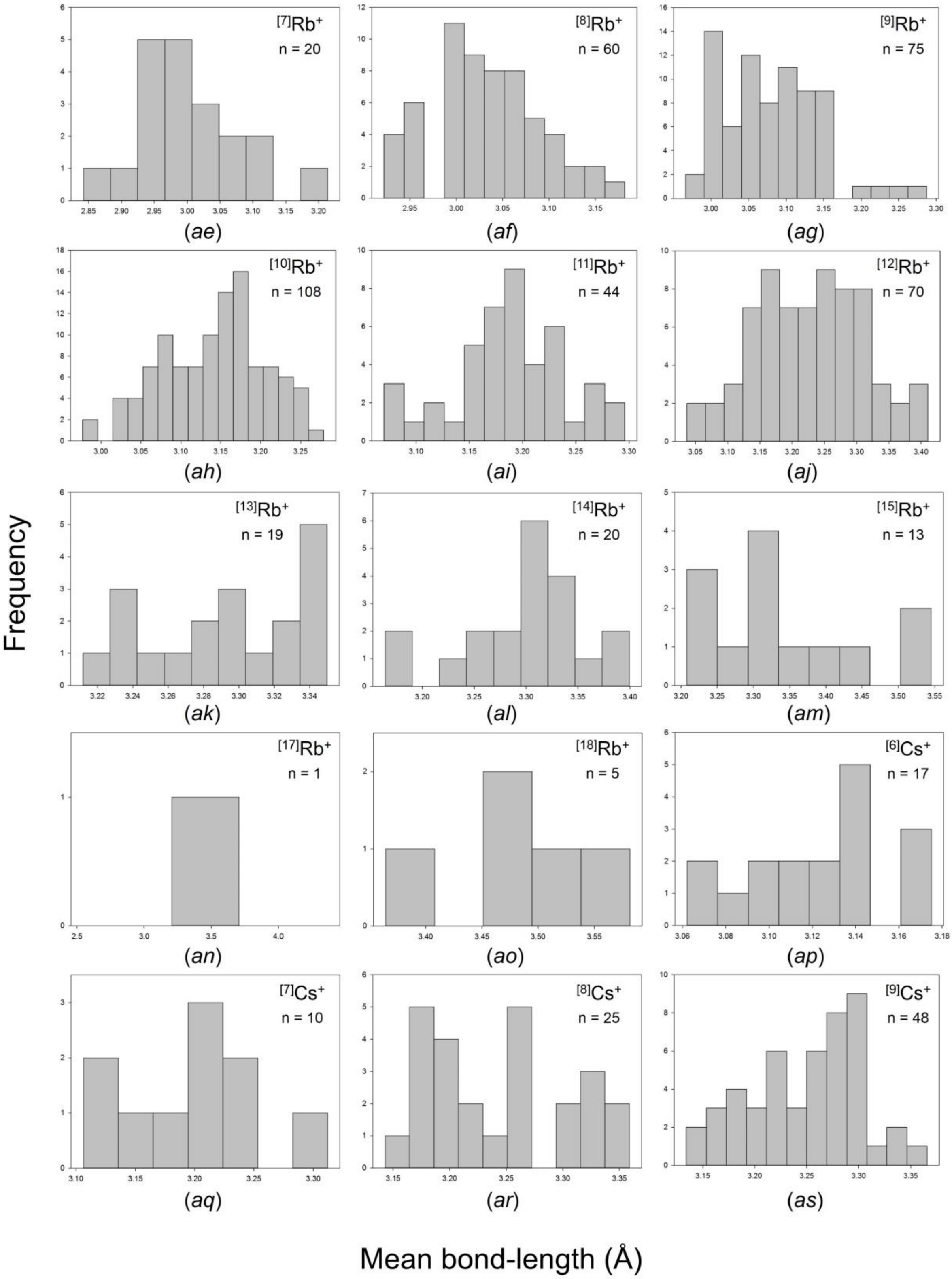

[7]Rb+
n = 20
(ae)
[8]Rb+
n = 60
(af)
[9]Rb+
n = 75
(ag)
[10]Rb+
n = 108
(ah)
[11]Rb+
n = 44
(ai)
[12]Rb+
n = 70
(aj)
[13]Rb+
n = 19
(ak)
[14]Rb+
n = 20
(al)
[15]Rb+
n = 13
(am)
[17]Rb+
n = 1
(an)
[18]Rb+
n = 5
(ao)
[6]Cs+
n = 17
(ap)
[7]Cs+
n = 10
(aq)
[8]Cs+
n = 25
(ar)
[9]Cs+
n = 48
(as)
Frequency
Mean bond-length (Å)

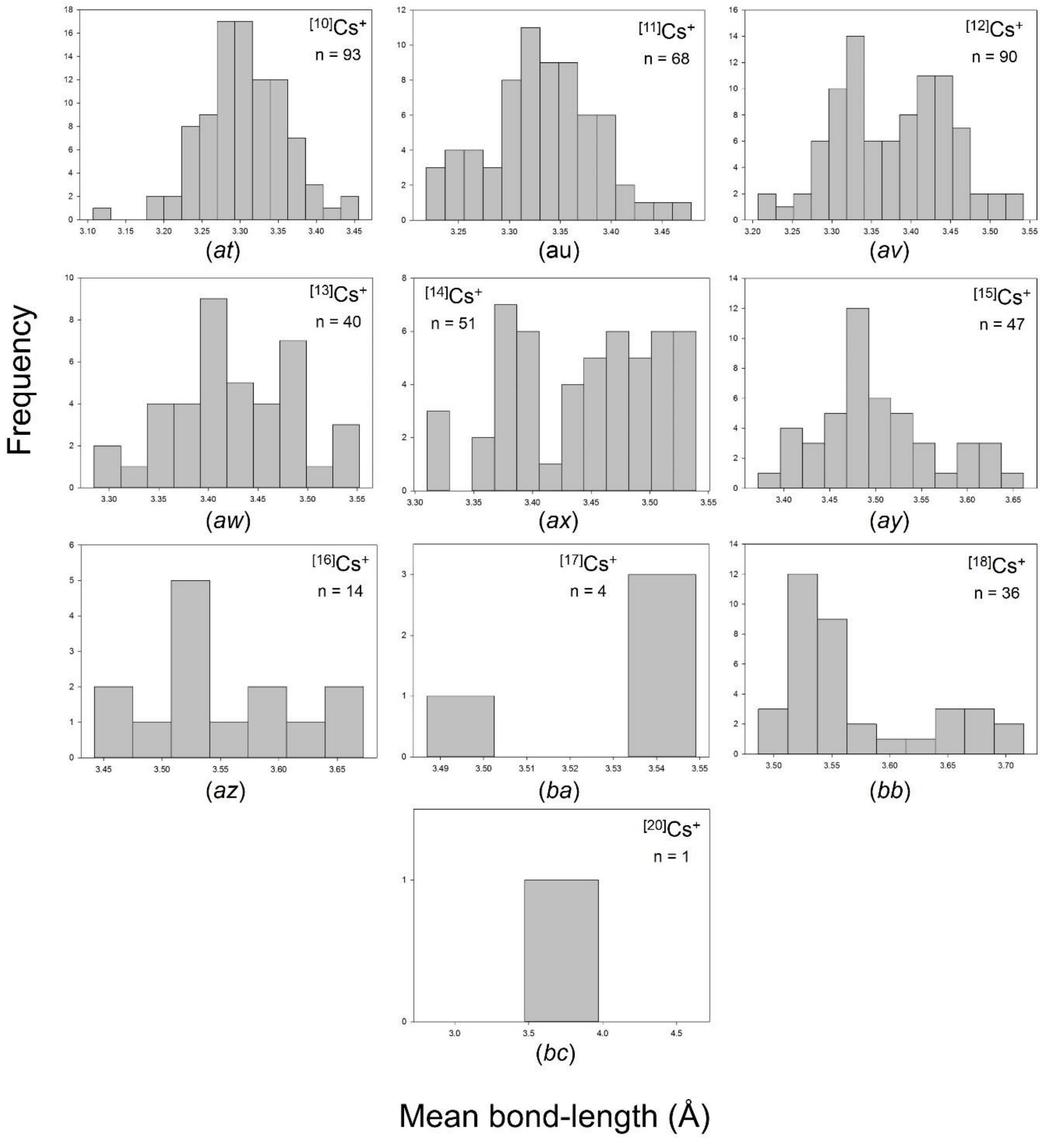

Figure S3 Mean bond-length distributions for all configurations of the alkali-metal ions bonded to O^{2-}:(*a*) $^{[3]}Li^+$, (*b*) $^{[4]}Li^+$, (*c*) $^{[5]}Li^+$, (*d*) $^{[6]}Li^+$, (*e*) $^{[7]}Li^+$, (*f*) $^{[8]}Li^+$, (*g*) $^{[3]}Na^+$, (*h*) $^{[4]}Na^+$, (*i*) $^{[5]}Na^+$, (*j*) $^{[6]}Na^+$, (*k*) $^{[7]}Na^+$, (*l*) $^{[8]}Na^+$, (*m*) $^{[9]}Na^+$, (*n*) $^{[10]}Na^+$, (*o*) $^{[12]}Na^+$, (*p*) $^{[4]}K^+$, (*q*) $^{[5]}K^+$, (*r*) $^{[6]}K^+$, (*s*) $^{[7]}K^+$, (*t*) $^{[8]}K^+$, (*u*) $^{[9]}K^+$, (*v*) $^{[10]}K^+$, (*w*) $^{[11]}K^+$, (*x*) $^{[12]}K^+$, (*y*) $^{[13]}K^+$, (*z*) $^{[14]}K^+$, (*aa*) $^{[15]}K^+$, (*ab*) $^{[4]}Rb^+$, (*ac*) $^{[5]}Rb^+$, (*ad*) $^{[6]}Rb^+$, (*ae*) $^{[7]}Rb^+$, (*af*) $^{[8]}Rb^+$, (*ag*) $^{[9]}Rb^+$, (*ah*) $^{[10]}Rb^+$, (*ai*) $^{[11]}Rb^+$, (*aj*) $^{[12]}Rb^+$, (*ak*) $^{[13]}Rb^+$, (*al*) $^{[14]}Rb^+$, (*am*) $^{[15]}Rb^+$, (*an*) $^{[17]}Rb^+$, (*ao*) $^{[18]}Rb^+$, (*ap*) $^{[6]}Cs^+$, (*aq*) $^{[7]}Cs^+$, (*ar*) $^{[8]}Cs^+$, (*as*) $^{[9]}Cs^+$, (*at*) $^{[10]}Cs^+$, (*au*) $^{[11]}Cs^+$, (*av*) $^{[12]}Cs^+$, (*aw*) $^{[13]}Cs^+$, (*ax*) $^{[14]}Cs^+$, (*ay*) $^{[15]}Cs^+$, (*az*) $^{[16]}Cs^+$, (*ba*) $^{[17]}Cs^+$, (*bb*) $^{[18]}Cs^+$, (*bc*) $^{[20]}Cs^+$.

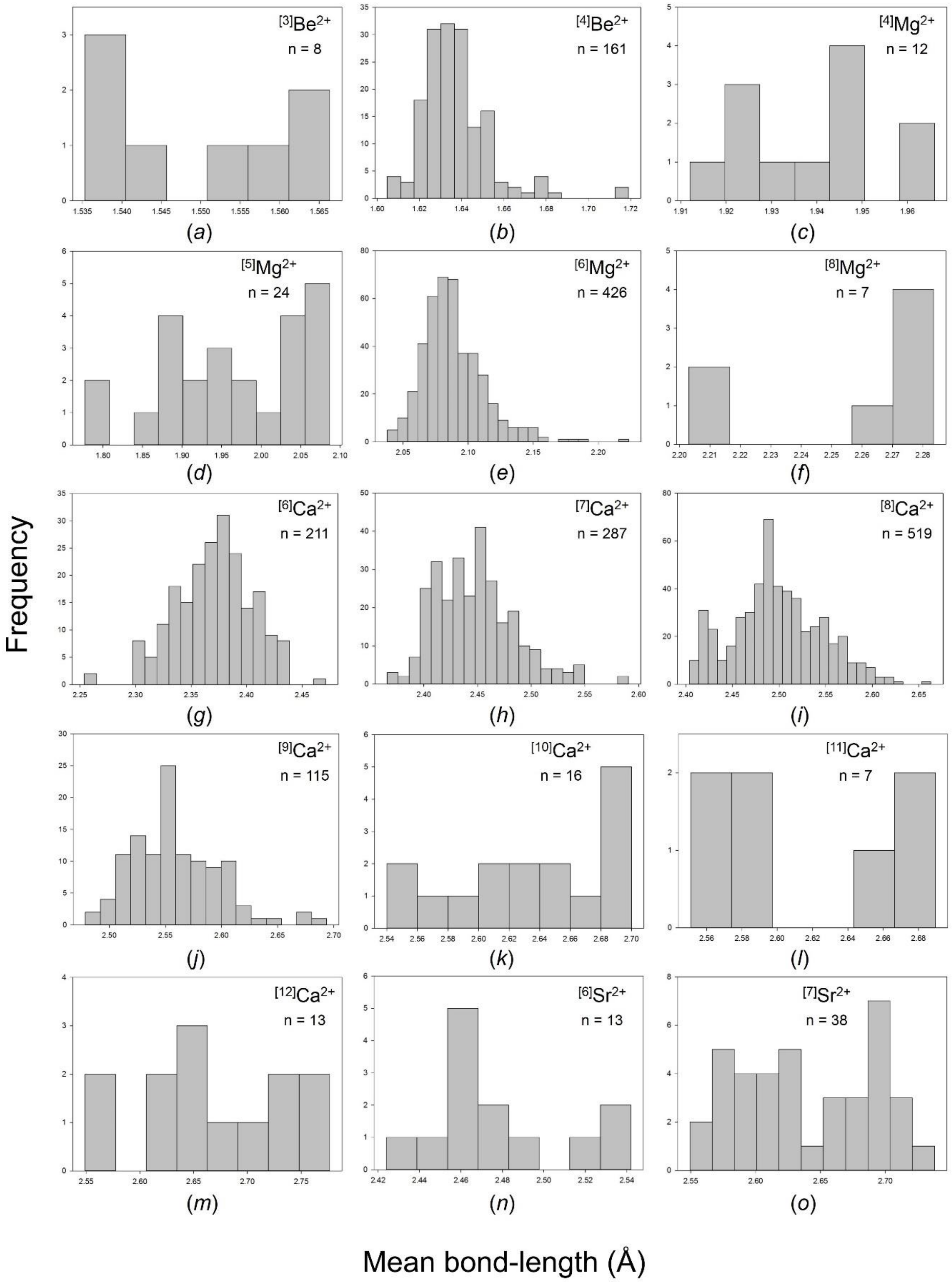

[3]Be2+
n = 8
(a)
[4]Be2+
n = 161
(b)
[4]Mg2+
n = 12
(c)
[5]Mg2+
n = 24
(d)
[6]Mg2+
n = 426
(e)
[8]Mg2+
n = 7
(f)
[6]Ca2+
n = 211
(g)
[7]Ca2+
n = 287
(h)
[8]Ca2+
n = 519
(i)
[9]Ca2+
n = 115
(j)
[10]Ca2+
n = 16
(k)
[11]Ca2+
n = 7
(l)
[12]Ca2+
n = 13
(m)
[6]Sr2+
n = 13
(n)
[7]Sr2+
n = 38
(o)
Frequency
Mean bond-length (Å)

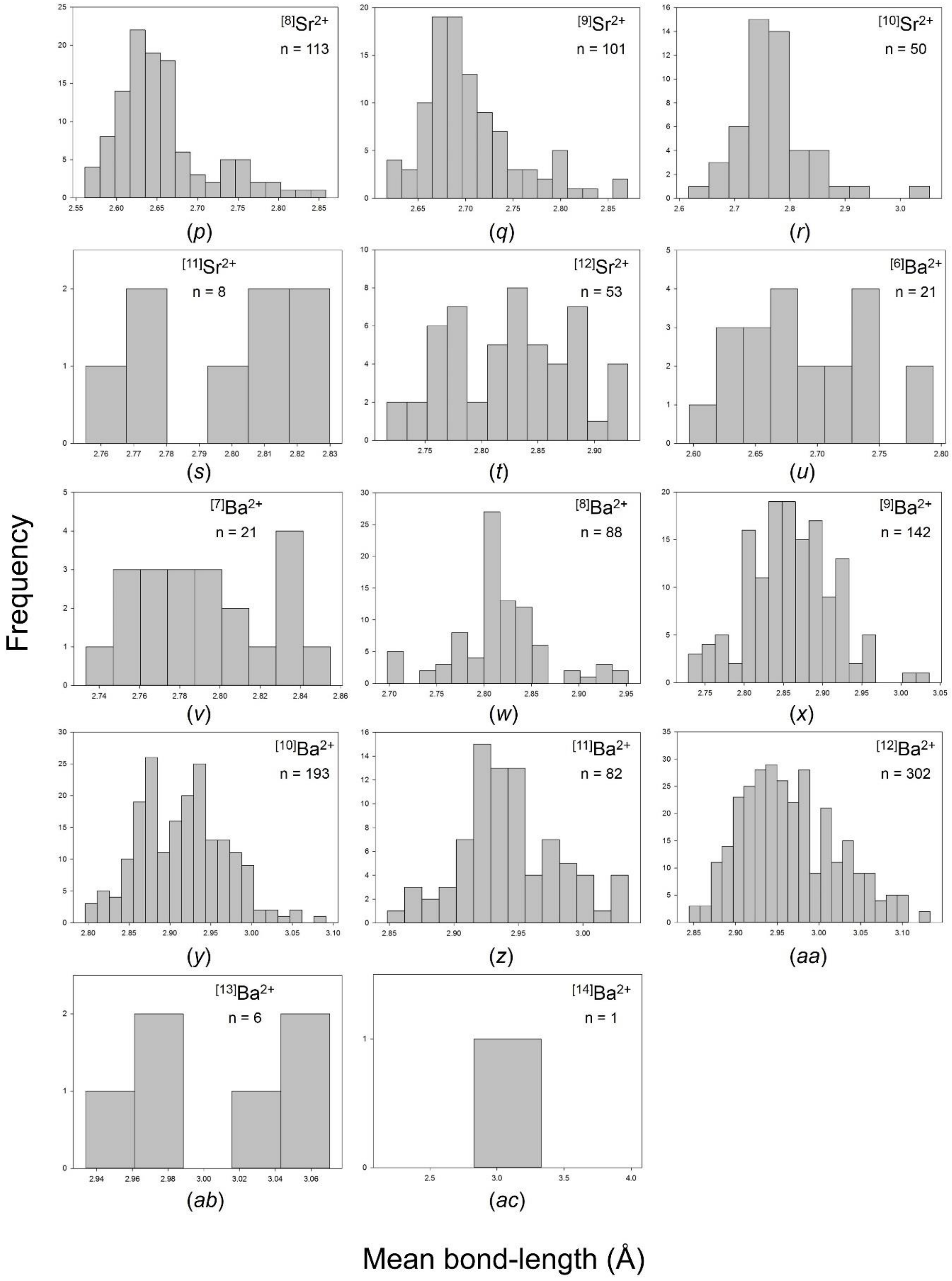
[8]Sr2+
n = 113
(p)
[9]Sr2+
n = 101
(q)
[10]Sr2+
n = 50
(r)
[11]Sr2+
n = 8
(s)
[12]Sr2+
n = 53
(t)
[6]Ba2+
n = 21
(u)
[7]Ba2+
n = 21
(v)
[8]Ba2+
n = 88
(w)
[9]Ba2+
n = 142
(x)
[10]Ba2+
n = 193
(y)
[11]Ba2+
n = 82
(z)
[12]Ba2+
n = 302
(aa)
[13]Ba2+
n = 6
(ab)
[14]Ba2+
n = 1
(ac)
Frequency
Mean bond-length (Å)

Figure S4 Mean bond-length distributions for all configurations of the alkaline-earth-metal ions bonded to O^{2-}:(*a*) $^{[3]}Be^{2+}$, (*b*) $^{[4]}Be^{2+}$, (*c*) $^{[4]}Mg^{2+}$, (*d*) $^{[5]}Mg^{2+}$, (*e*) $^{[6]}Mg^{2+}$, (*f*) $^{[8]}Mg^{2+}$, (*g*) $^{[6]}Ca^{2+}$, (*h*) $^{[7]}Ca^{2+}$, (*i*) $^{[8]}Ca^{2+}$, (*j*) $^{[9]}Ca^{2+}$, (*k*) $^{[10]}Ca^{2+}$, (*l*) $^{[11]}Ca^{2+}$, (*m*) $^{[12]}Ca^{2+}$, (*n*) $^{[6]}Sr^{2+}$, (*o*) $^{[7]}Sr^{2+}$, (*p*) $^{[8]}Sr^{2+}$, (*q*) $^{[9]}Sr^{2+}$, (*r*) $^{[10]}Sr^{2+}$, (*s*) $^{[11]}Sr^{2+}$, (*t*) $^{[12]}Sr^{2+}$, (*u*) $^{[6]}Ba^{2+}$, (*v*) $^{[7]}Ba^{2+}$, (*w*) $^{[8]}Ba^{2+}$, (*x*) $^{[9]}Ba^{2+}$, (*y*) $^{[10]}Ba^{2+}$, (*z*) $^{[11]}Ba^{2+}$, (*aa*) $^{[12]}Ba^{2+}$, (*ab*) $^{[13]}Ba^{2+}$, (*ac*) $^{[14]}Ba^{2+}$.

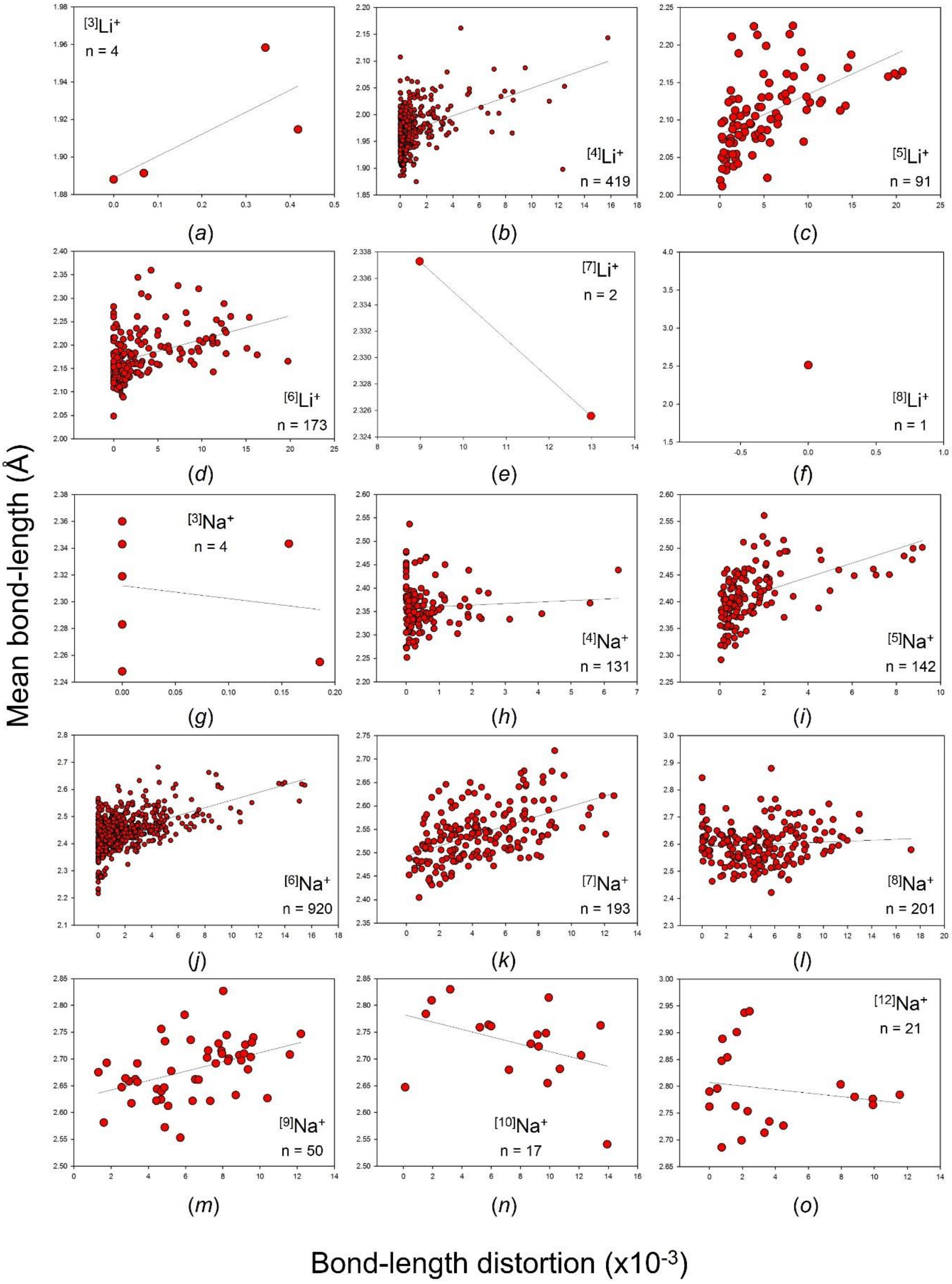

[3]Li+
n = 4
(a)
[4]Li+
n = 419
(b)
[5]Li+
n = 91
(c)
[6]Li+
n = 173
(d)
[7]Li+
n = 2
(e)
[8]Li+
n = 1
(f)
[3]Na+
n = 4
(g)
[4]Na+
n = 131
(h)
[5]Na+
n = 142
(i)
[6]Na+
n = 920
(j)
[7]Na+
n = 193
(k)
[8]Na+
n = 201
(l)
[9]Na+
n = 50
(m)
[10]Na+
n = 17
(n)
[12]Na+
n = 21
(o)
Mean bond-length (Å)
Bond-length distortion (x10-3)

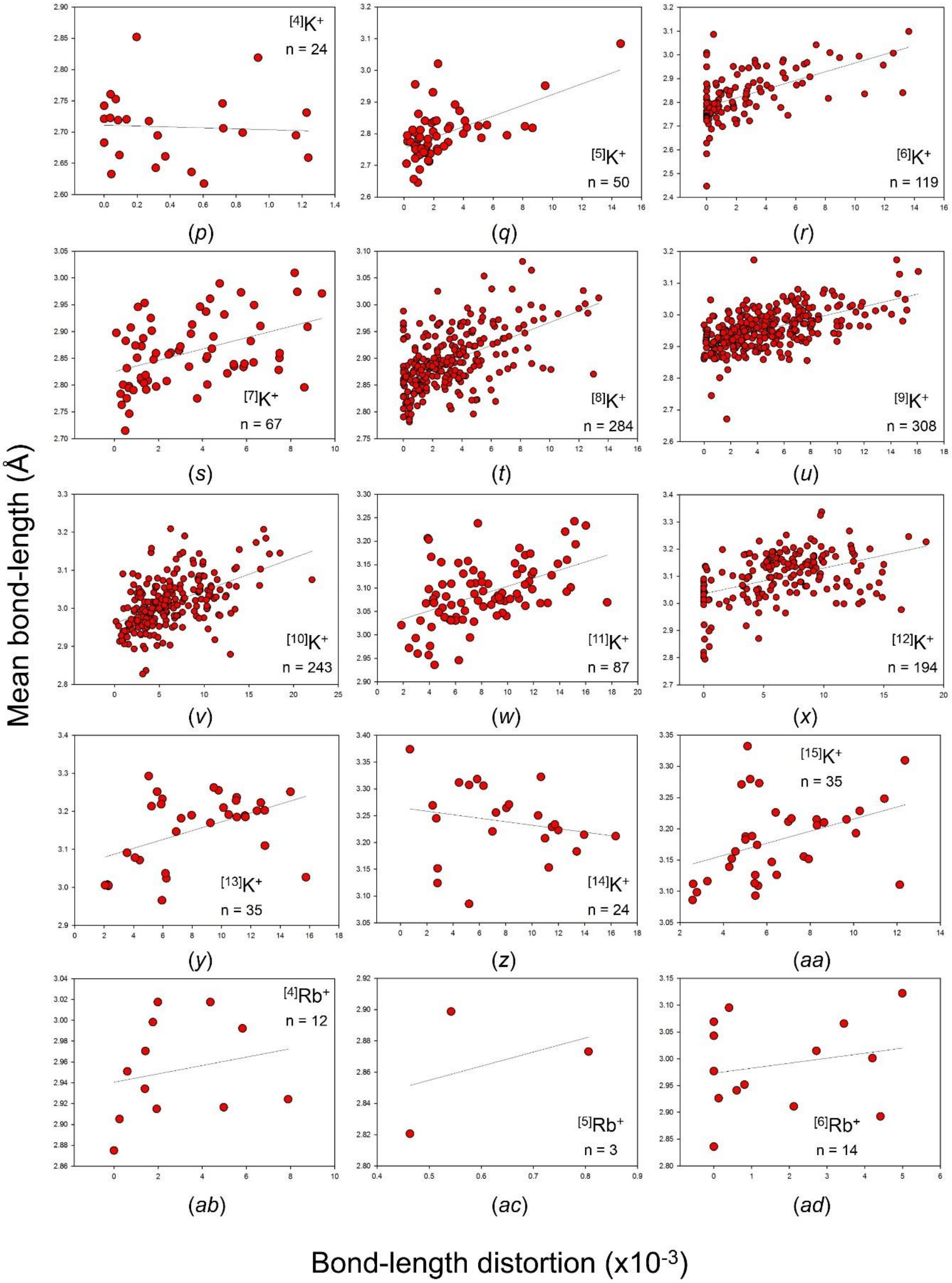
[4]K+
n = 24
[5]K+
n = 50
[6]K+
n = 119
(p)
(q)
(r)
[7]K+
n = 67
[8]K+
n = 284
[9]K+
n = 308
(s)
(t)
(u)
[10]K+
n = 243
[11]K+
n = 87
[12]K+
n = 194
(v)
(w)
(x)
[13]K+
n = 35
[14]K+
n = 24
[15]K+
n = 35
(y)
(z)
(aa)
[4]Rb+
n = 12
[5]Rb+
n = 3
[6]Rb+
n = 14
(ab)
(ac)
(ad)
Mean bond-length (Å)
Bond-length distortion (x10-3)

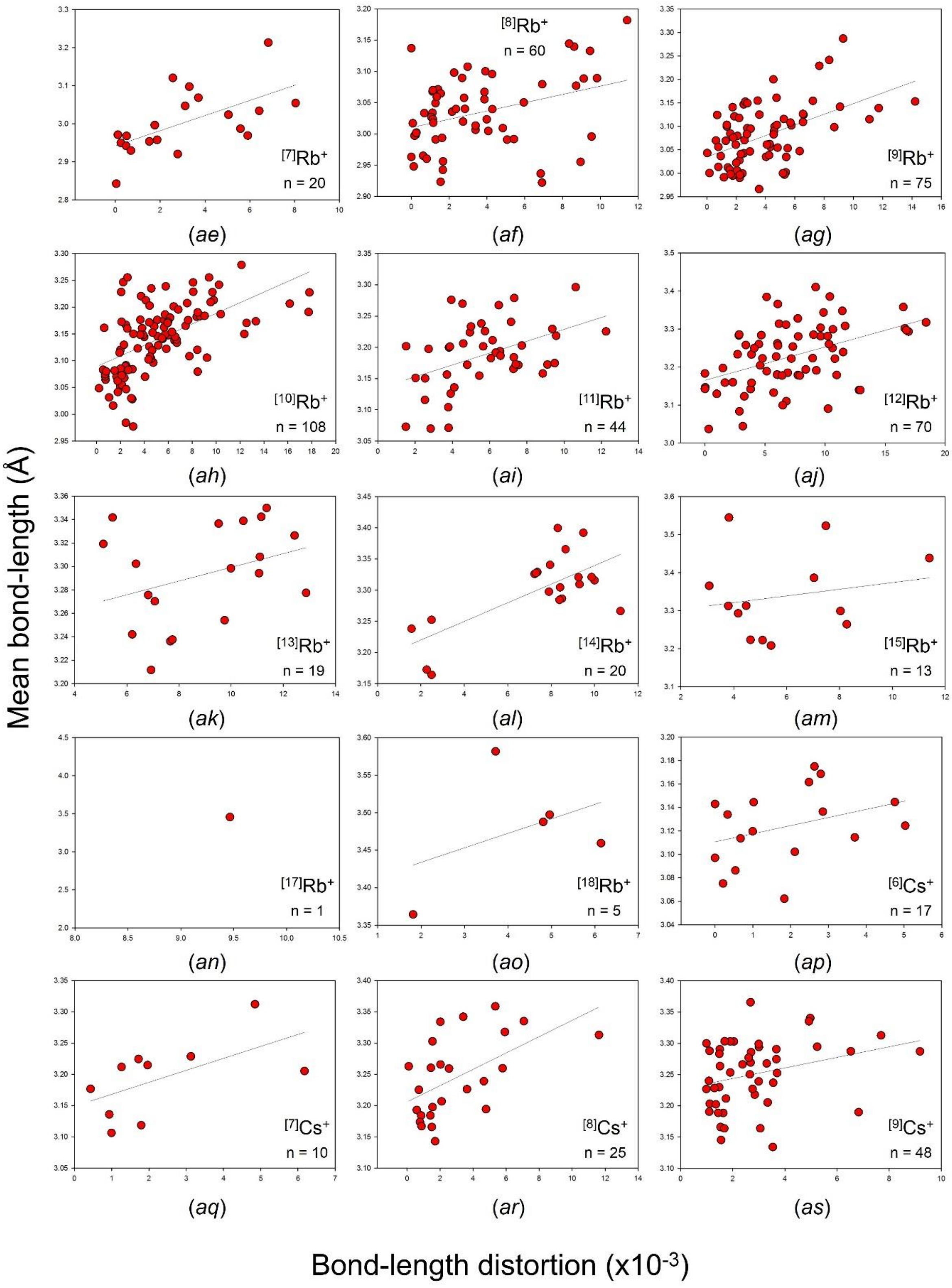
[7]Rb+
n = 20
(ae)
[8]Rb+
n = 60
(af)
[9]Rb+
n = 75
(ag)
[10]Rb+
n = 108
(ah)
[11]Rb+
n = 44
(ai)
[12]Rb+
n = 70
(aj)
[13]Rb+
n = 19
(ak)
[14]Rb+
n = 20
(al)
[15]Rb+
n = 13
(am)
[17]Rb+
n = 1
(an)
[18]Rb+
n = 5
(ao)
[6]Cs+
n = 17
(ap)
[7]Cs+
n = 10
(aq)
[8]Cs+
n = 25
(ar)
[9]Cs+
n = 48
(as)
Mean bond-length (Å)
Bond-length distortion (x10-3)

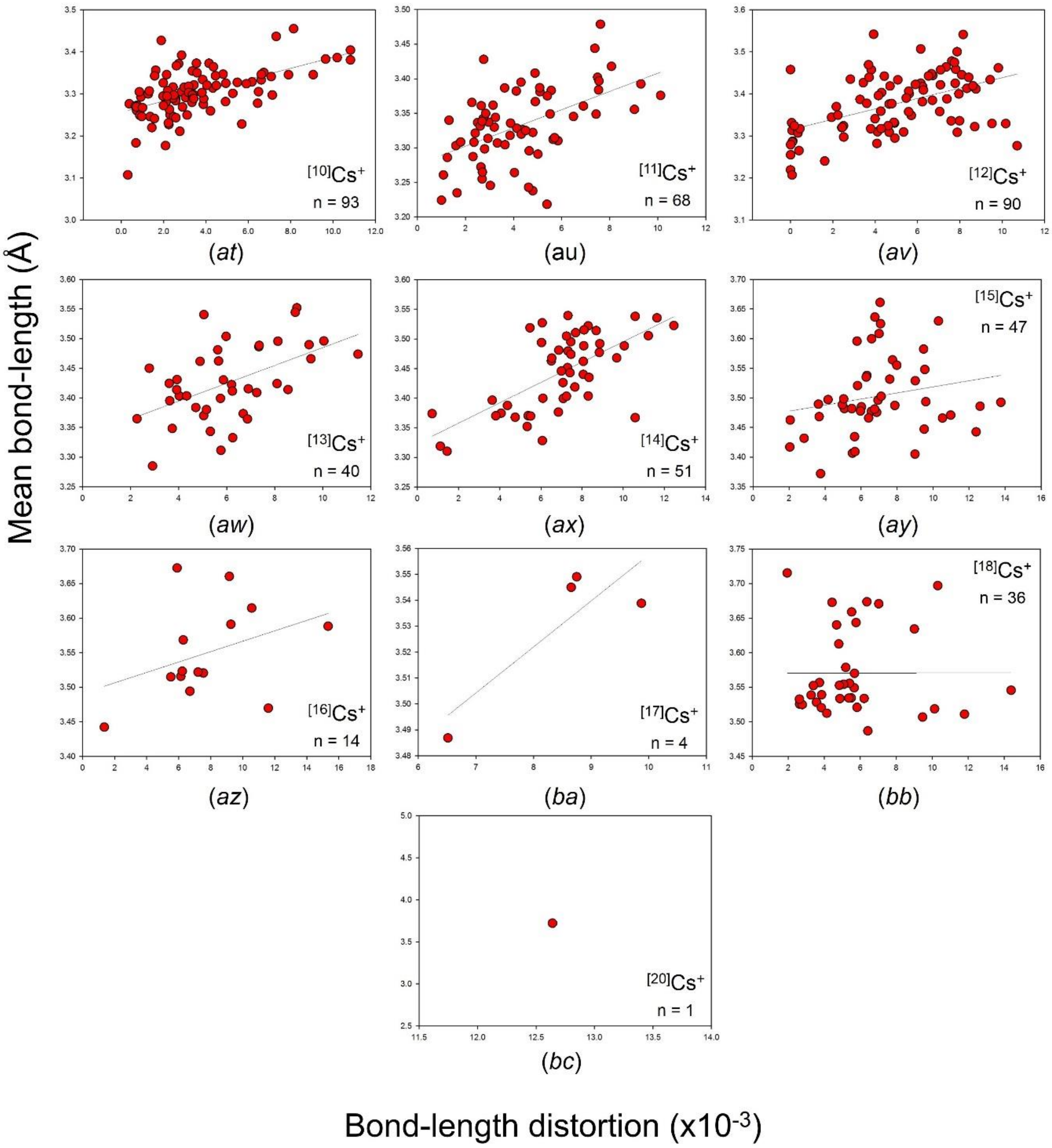

Figure S5 The effect of bond-length distortion on mean bond-length for all configurations of the alkaline-earth-metal ions bonded to O^{2-}:(*a*) $^{[3]}Li^+$, (*b*) $^{[4]}Li^+$, (*c*) $^{[5]}Li^+$, (*d*) $^{[6]}Li^+$, (*e*) $^{[7]}Li^+$, (*f*) $^{[8]}Li^+$, (*g*) $^{[3]}Na^+$, (*h*) $^{[4]}Na^+$, (*i*) $^{[5]}Na^+$, (*j*) $^{[6]}Na^+$, (*k*) $^{[7]}Na^+$, (*l*) $^{[8]}Na^+$, (*m*) $^{[9]}Na^+$, (*n*) $^{[10]}Na^+$, (*o*) $^{[12]}Na^+$, (*p*) $^{[4]}K^+$, (*q*) $^{[5]}K^+$, (*r*) $^{[6]}K^+$, (*s*) $^{[7]}K^+$, (*t*) $^{[8]}K^+$, (*u*) $^{[9]}K^+$, (*v*) $^{[10]}K^+$, (*w*) $^{[11]}K^+$, (*x*) $^{[12]}K^+$, (*y*) $^{[13]}K^+$, (*z*) $^{[14]}K^+$, (*aa*) $^{[15]}K^+$, (*ab*) $^{[4]}Rb^+$, (*ac*) $^{[5]}Rb^+$, (*ad*) $^{[6]}Rb^+$, (*ae*) $^{[7]}Rb^+$, (*af*) $^{[8]}Rb^+$, (*ag*) $^{[9]}Rb^+$, (*ah*) $^{[10]}Rb^+$, (*ai*) $^{[11]}Rb^+$, (*aj*) $^{[12]}Rb^+$, (*ak*) $^{[13]}Rb^+$, (*al*) $^{[14]}Rb^+$, (*am*) $^{[15]}Rb^+$, (*an*) $^{[17]}Rb^+$, (*ao*) $^{[18]}Rb^+$, (*ap*) $^{[6]}Cs^+$, (*aq*) $^{[7]}Cs^+$, (*ar*) $^{[8]}Cs^+$, (*as*) $^{[9]}Cs^+$, (*at*) $^{[10]}Cs^+$, (*au*) $^{[11]}Cs^+$, (*av*) $^{[12]}Cs^+$, (*aw*) $^{[13]}Cs^+$, (*ax*) $^{[14]}Cs^+$, (*ay*) $^{[15]}Cs^+$, (*az*) $^{[16]}Cs^+$, (*ba*) $^{[17]}Cs^+$, (*bb*) $^{[18]}Cs^+$, (*bc*) $^{[20]}Cs^+$.

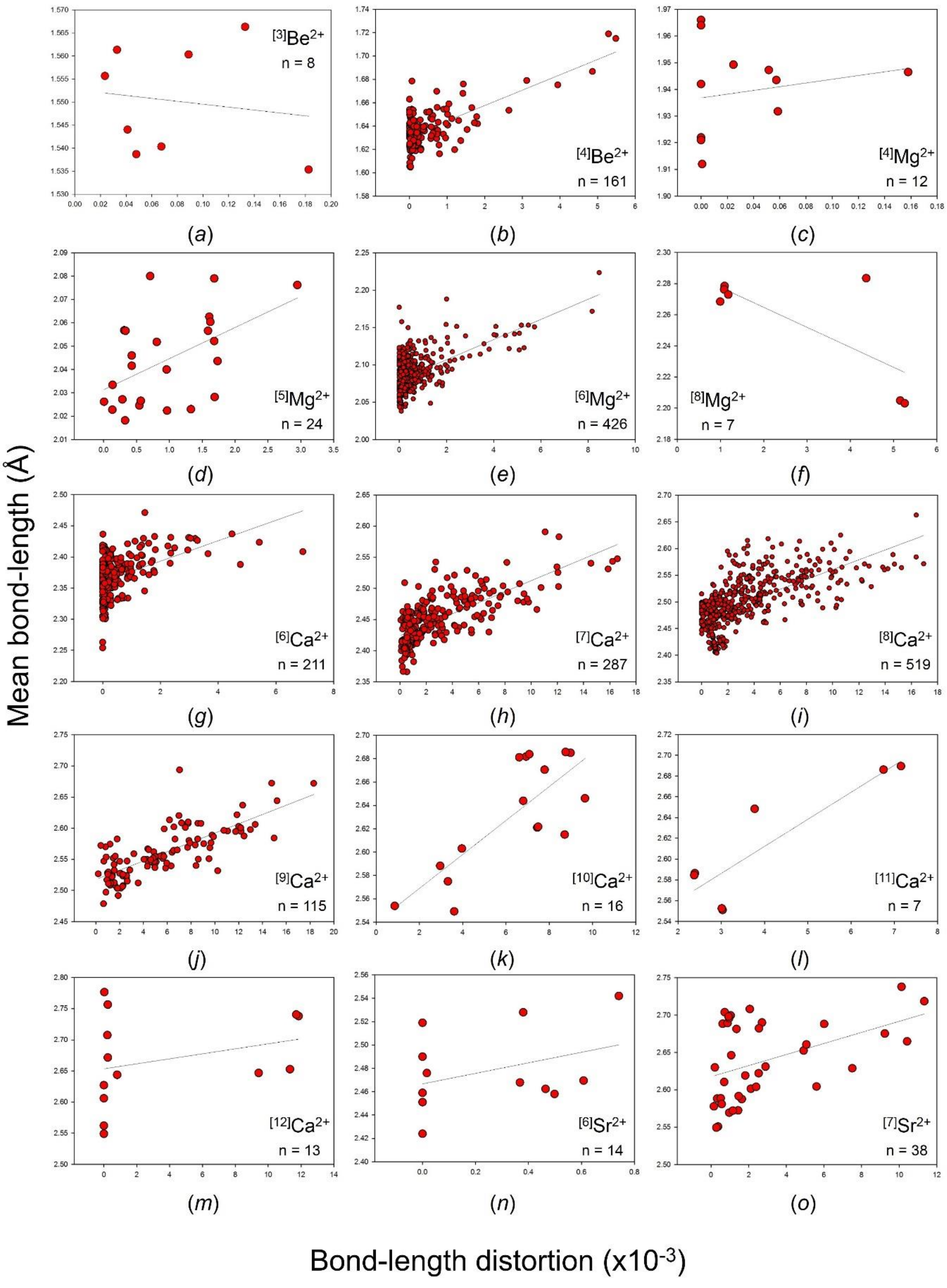

Mean bond-length (Å)
Bond-length distortion (x10-3)
[3]Be2+
n = 8
(a)
[4]Be2+
n = 161
(b)
[4]Mg2+
n = 12
(c)
[5]Mg2+
n = 24
(d)
[6]Mg2+
n = 426
(e)
[8]Mg2+
n = 7
(f)
[6]Ca2+
n = 211
(g)
[7]Ca2+
n = 287
(h)
[8]Ca2+
n = 519
(i)
[9]Ca2+
n = 115
(j)
[10]Ca2+
n = 16
(k)
[11]Ca2+
n = 7
(l)
[12]Ca2+
n = 13
(m)
[6]Sr2+
n = 14
(n)
[7]Sr2+
n = 38
(o)

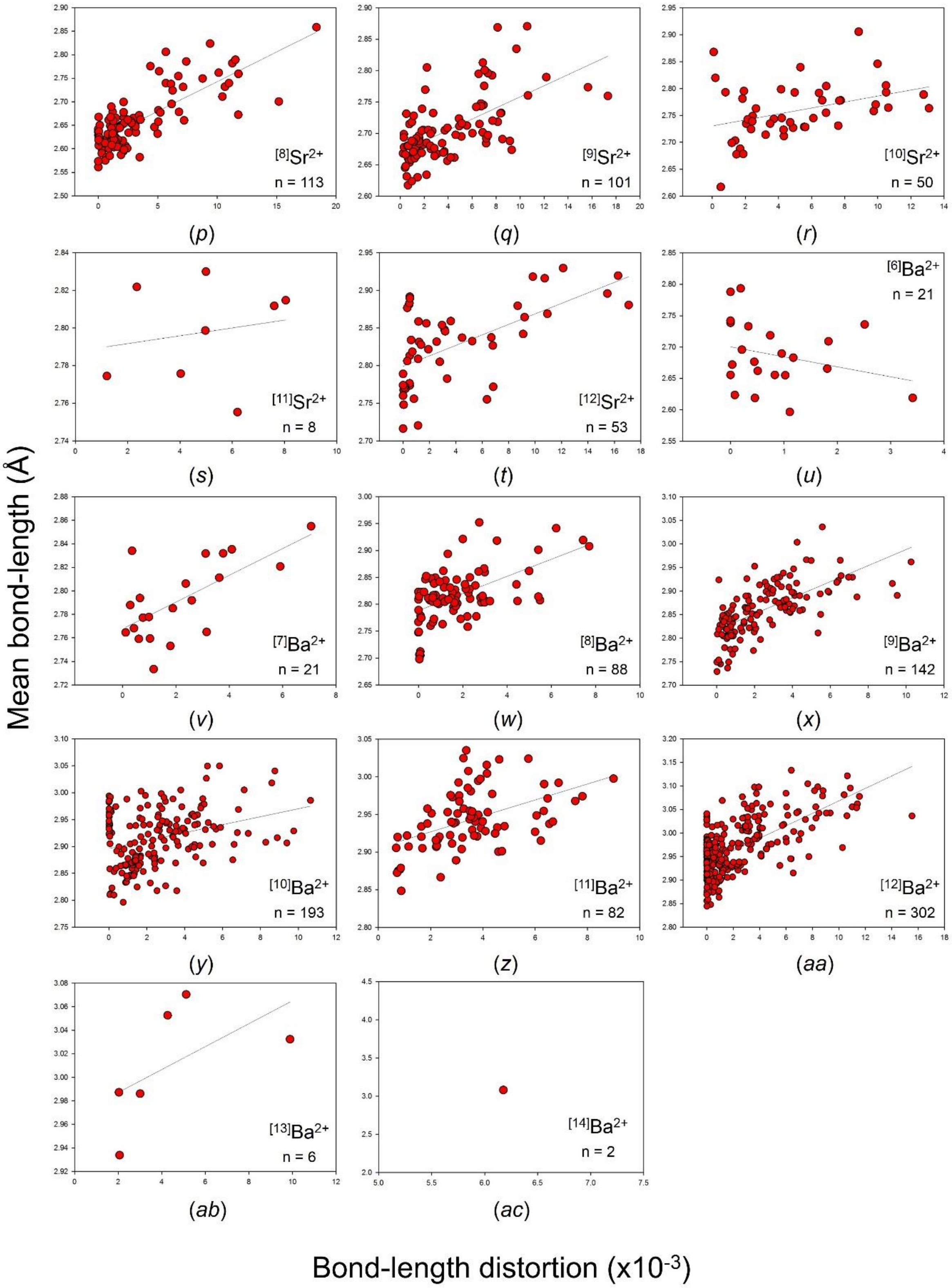

[8]Sr2+
n = 113
(p)
[9]Sr2+
n = 101
(q)
[10]Sr2+
n = 50
(r)
[11]Sr2+
n = 8
(s)
[12]Sr2+
n = 53
(t)
[6]Ba2+
n = 21
(u)
[7]Ba2+
n = 21
(v)
[8]Ba2+
n = 88
(w)
[9]Ba2+
n = 142
(x)
[10]Ba2+
n = 193
(y)
[11]Ba2+
n = 82
(z)
[12]Ba2+
n = 302
(aa)
[13]Ba2+
n = 6
(ab)
[14]Ba2+
n = 2
(ac)
Mean bond-length (Å)
Bond-length distortion (x10-3)

Figure S6 The effect of bond-length distortion on mean bond-length for all configurations of the alkaline-earth-metal ions bonded to O^{2-}: (*a*) $^{[3]}Be^{2+}$, (*b*) $^{[4]}Be^{2+}$, (*c*) $^{[4]}Mg^{2+}$, (*d*) $^{[5]}Mg^{2+}$, (*e*) $^{[6]}Mg^{2+}$, (*f*) $^{[8]}Mg^{2+}$, (*g*) $^{[6]}Ca^{2+}$, (*h*) $^{[7]}Ca^{2+}$, (*i*) $^{[8]}Ca^{2+}$, (*j*) $^{[9]}Ca^{2+}$, (*k*) $^{[10]}Ca^{2+}$, (*l*) $^{[11]}Ca^{2+}$, (*m*) $^{[12]}Ca^{2+}$, (*n*) $^{[6]}Sr^{2+}$, (*o*) $^{[7]}Sr^{2+}$, (*p*) $^{[8]}Sr^{2+}$, (*q*) $^{[9]}Sr^{2+}$, (*r*) $^{[10]}Sr^{2+}$, (*s*) $^{[11]}Sr^{2+}$, (*t*) $^{[12]}Sr^{2+}$, (*u*) $^{[6]}Ba^{2+}$, (*v*) $^{[7]}Ba^{2+}$, (*w*) $^{[8]}Ba^{2+}$, (*x*) $^{[9]}Ba^{2+}$, (*y*) $^{[10]}Ba^{2+}$, (*z*) $^{[11]}Ba^{2+}$, (*aa*) $^{[12]}Ba^{2+}$, (*ab*) $^{[13]}Ba^{2+}$, (*ac*) $^{[14]}Ba^{2+}$.
